# Oxalate Oxidase for In Situ H_2_O_2_‐Generation in Unspecific Peroxygenase‐Catalysed Drug Oxyfunctionalisations**

**DOI:** 10.1002/anie.202207831

**Published:** 2022-08-25

**Authors:** Elvira Romero, Magnus J. Johansson, Jared Cartwright, Gideon Grogan, Martin A. Hayes

**Affiliations:** ^1^ Compound Synthesis and Management Discovery Sciences BioPharmaceuticals R&D AstraZeneca Pepparedsleden 1 43183 Mölndal Sweden; ^2^ Medicinal Chemistry Research and Early Development Cardiovascular, Renal and Metabolism (CVRM) BioPharmaceuticals R&D AstraZeneca Pepparedsleden 1 43183 Mölndal Sweden; ^3^ Department of Organic Chemistry Stockholm University Svante Arrhenius väg 16 C 10691 Stockholm Sweden; ^4^ Department of Biology University of York Heslington York YO10 5DD UK; ^5^ Department of Chemistry University of York Heslington York YO10 5DD UK

**Keywords:** Biocatalysis, Drug Late-Stage Functionalisation, H_2_O_2_-Generation, High-Throughput Screening, Oxidoreductases

## Abstract

H_2_O_2_‐driven enzymes are of great interest for industrial biotransformations. Herein, we show for the first time that oxalate oxidase (OXO) is an efficient in situ source of H_2_O_2_ for one of these biocatalysts, which is known as unspecific peroxygenase (UPO). OXO is reasonably robust, produces only CO_2_ as a by‐product and uses oxalate as a cheap sacrificial electron donor. UPO has significant potential as an industrial catalyst for selective C−H oxyfunctionalisations, as we confirm herein by testing a diverse drug panel using miniaturised high‐throughput assays and mass spectrometry. 33 out of 64 drugs were converted in 5 μL‐scale reactions by the UPO with OXO (conversion >70 % for 11 drugs). Furthermore, oxidation of the drug tolmetin was achieved on a 50 mg scale (TON_UPO_ 25 664) with 84 % yield, which was further improved via enzyme immobilization. This one‐pot approach ensures adequate H_2_O_2_ levels, enabling rapid access to industrially relevant molecules that are difficult to obtain by other routes.

## Graphical abstract

High‐throughput μL‐scale screenings revealed optimal conditions for in situ H_2_O_2_‐generation using oxalate oxidase in bioconversions catalysed by unspecific peroxygenase. This enzymatic tandem exhibits extraordinary potential for selective C−H oxyfunctionalisation reactions of complex drug scaffolds.

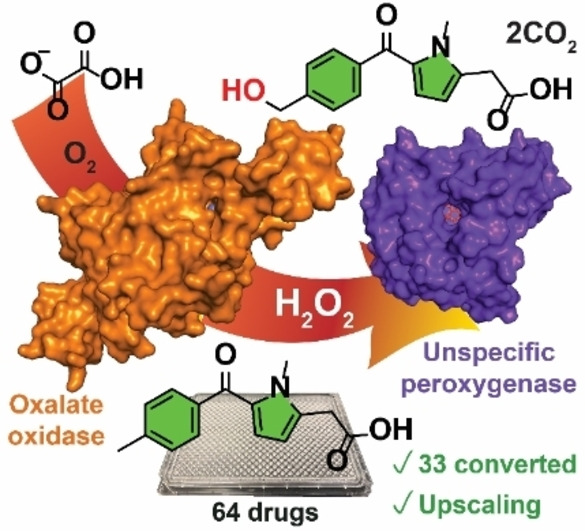

Enzymes are gaining increasing importance in industrial synthetic chemistry.^[1]^ They often exhibit excellent selectivity, high catalytic efficiency under mild‐reaction conditions and deliver reduced amounts of by‐products, in contrast to traditional chemical catalysts. However, the full potential of enzyme‐catalysed synthesis in industry is yet to be exploited. Among oxidoreductases, hydrogen peroxide (H_2_O_2_)‐driven enzymes exhibit various advantages in industrial processes, compared to other enzymes which require expensive cofactors such as NADPH.^[2]^ H_2_O_2_ is a powerful oxidizing agent that is cheap, relatively safe and only produces water as a by‐product. Oxidoreductases that use H_2_O_2_ as an electron acceptor include unspecific peroxygenases (UPOs, EC 1.11.2.1). These enzymes, which incorporate one oxygen atom from H_2_O_2_ into the reaction product, have attracted much attention over the past decade. UPOs catalyse C−H oxyfunctionalisations of a wide variety of industrially relevant molecules, offering advantages over their transition metal catalyst counterparts in terms of selectivity and sustainability.^[3]^ UPOs and cytochromes P450 (P450, EC 1.14.14.1) catalyse similar reactions, but UPOs are more robust and do not require expensive cofactors or redox partners.^[4]^

Implementation of UPOs in industrial synthesis would be significantly facilitated by more effective approaches to supply adequate levels of H_2_O_2_ in the reactions.^[2]^ Excessive levels of H_2_O_2_ lead to irreversible inactivation of the UPO by heme degradation (Scheme 1). To overcome this, H_2_O_2_ can be slowly added to reactions using a syringe pump or in aliquots. However, the palladium‐catalysed anthraquinone process, which is generally used for large‐scale manufacture of H_2_O_2_, is not environmentally benign.^[5]^ Thus, a more sustainable alternative is in situ H_2_O_2_‐production.

**Scheme 1:**
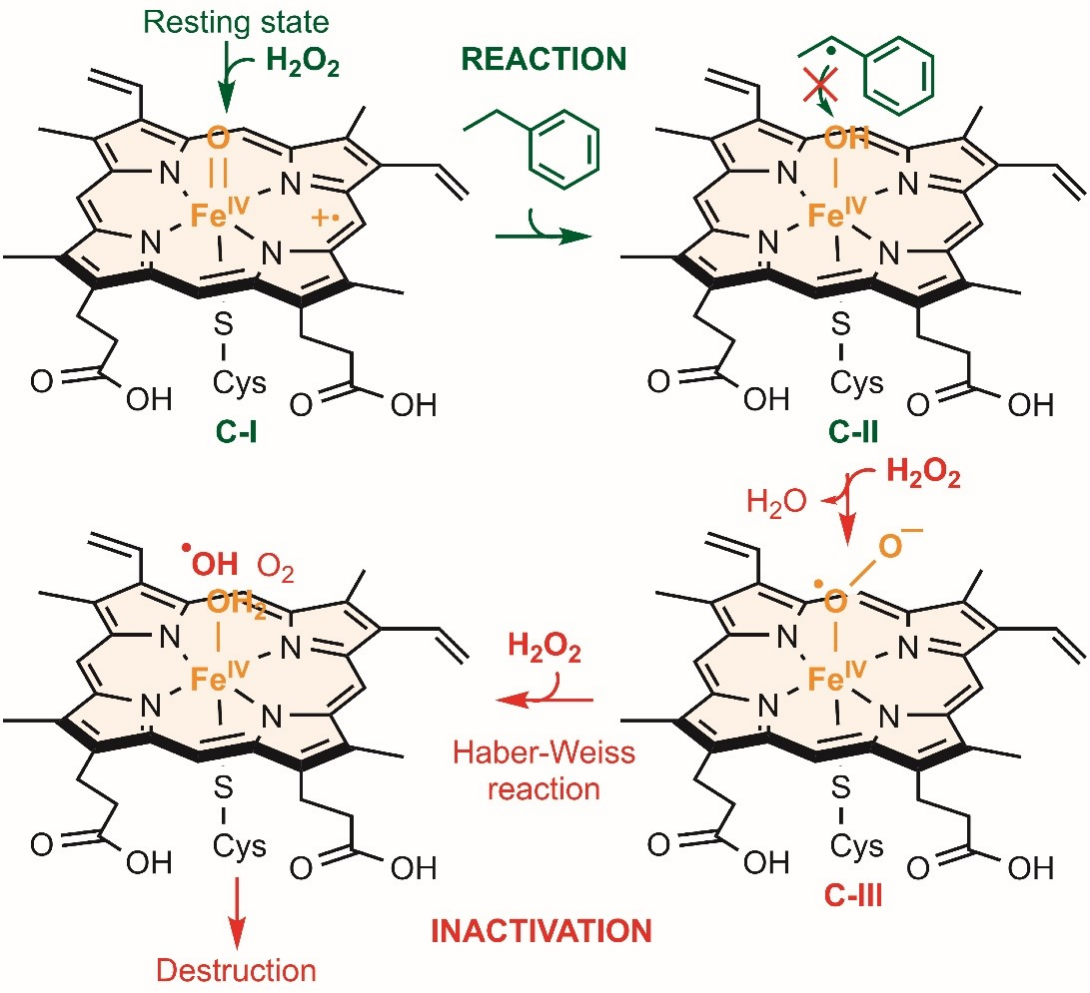
H_2_O_2_‐driven PaDa‐I inactivation. **C‐I** abstracts one electron and one proton from either an organic substrate or H_2_O_2_. **C‐II** reacts with H_2_O_2_ to form **C‐III** instead of reacting with an organic substrate radical if there is excess of H_2_O_2_. Next, reaction between **C‐III** and H_2_O_2_ yields hydroxyl radicals which hydroxylate the heme.^[9]^ Contrarily, an organic substrate radical is hydroxylated by **C‐II** and the UPO resting state is formed if the H_2_O_2_ levels are adequate.

Numerous in situ H_2_O_2_‐generation systems have been investigated to mitigate UPO inactivation that involve chemical, electrochemical, photochemical, photoelectrochemical, photoenzymatic, mechanical or enzymatic strategies.[^4^, ^6^] The most frequent drawbacks for non‐enzyme based technologies are production of hydroxyl radicals, substrate overoxidation, low turnover number, complex reactors/procedures, high energy input, undesired by‐products or photocatalyst photobleaching (Table S1). Oxidase‐based H_2_O_2_‐generation systems have therefore attracted a great deal of attention because they are user‐friendly, cost‐effective and sustainable (Scheme 2). Among these, the implementation of formate oxidase (FOx, EC 1.2.3.1) is especially interesting since it only produces CO_2_ as a by‐product,^[7]^ but a more robust FOx with a lower *K*_m_ is desired.^[7a]^ Inspired by the studies on FOx, we show here for the first time that oxalate oxidase (OXO, EC 1.2.3.4)^[8]^ is an efficient alternative as an H_2_O_2_‐source for the C−H oxyfunctionalisation reactions catalysed by UPOs. Currently, OXO is used to determine plasma and urine oxalate levels,^[10]^ to produce transgenic crops with an increased oxalate tolerance^[11]^ and in biofuel cells.^[12]^ Using high‐throughput liquid handling and mass spectroscopy, we have determined the influence of various parameters on UPO and OXO reactions at μL‐scale and then identified the products for diverse pharmaceutically relevant substrates using UPLC‐QTOF/MS^E^ data. In addition, we successfully scaled up one of these reactions. These studies demonstrate that OXO serves as an efficient H_2_O_2_‐generation system adding high value to the rapidly‐increasing toolbox for industrial biotechnology.

**Scheme 2:**
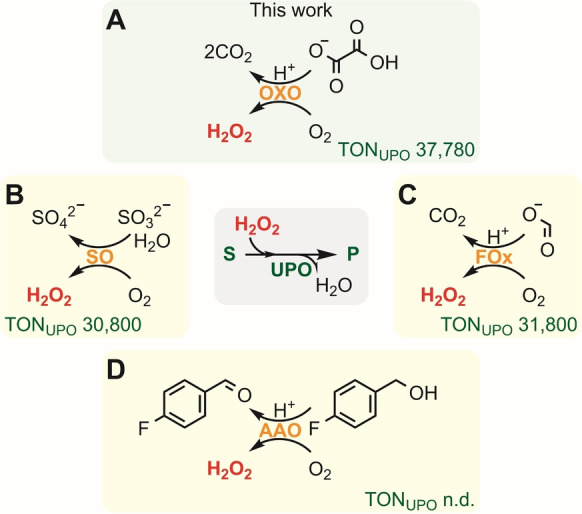
In situ oxidase‐based H_2_O_2_‐generation systems. For UPO‐catalysed conversion of ethylbenzene (**S**) into (*R*)‐1‐phenylethanol (**P**), **TON_UPO_** values (μmol product/μmol UPO) are indicated. Reaction of OXO (A), sulfite oxidase (**SO**, EC 1.8.3.1)^[13]^ (B), FOx (C) and aryl‐alcohol oxidase (**AAO**, EC 1.1.3.7)^[14]^ (D) are shown. n.d., not determined.

Previous studies showed that *Hordeum vulgare* (barley) OXO (HvOXO) is very resistant to heat and proteases and can be expressed in yeast.^[8b]^ This prompted us to select HvOXO for our H_2_O_2_‐generation system. In the present study, HvOXO was produced by fermentation of *Komagataella phaffii* (*Pichia pastoris*) and purified using Ni‐sepharose resin (Figure S2). To assess the efficiency of the HvOXO‐oxalate system for H_2_O_2_‐generation, the previously studied *Agrocybe aegerita* UPO (*Aae*UPO) variant, known as PaDa‐I,^[15]^ was used in all C−H oxyfunctionalisations. Expression and purification of PaDa‐I were performed following similar protocols to those previously described (Figure S2).^[16]^

After producing both enzymes, we compared their stability and pH optima to assess their compatibility. First, their melting temperature (*T*_m_) was determined by the ThermoFluor method (Figure 1 and S3). HvOXO *T*_m_ values were higher than 50 °C at all assayed pHs and its long‐term stability was also satisfactory (Figure S4). Higher thermostability was similarly observed for PaDa‐I at pH 4.0 and 5.0, though PaDa‐I was more rapidly inactivated at pH 3.0 (Figure S4). Next, we studied the influence of pH on the kinetic parameters for HvOXO. *k*_cat_ and *K*_m(oxalate)_ values increased 3‐ and 244‐fold as the pH was raised from 3.0 to 5.0, respectively (Table 1, Figure S5–6). These studies also showed that HvOXO exhibits strong substrate inhibition at pH 3.0, but not at pH 4.0 so this was the preferred pH for HvOXO reactions with up to 15 mM oxalate. However, pH 5.0 is the best option when a higher oxalate concentration is required. Conveniently, the optimal pH of PaDa‐I for activity is usually slightly acidic, but varies depending on the substrate.^[15]^

**Figure 1:**
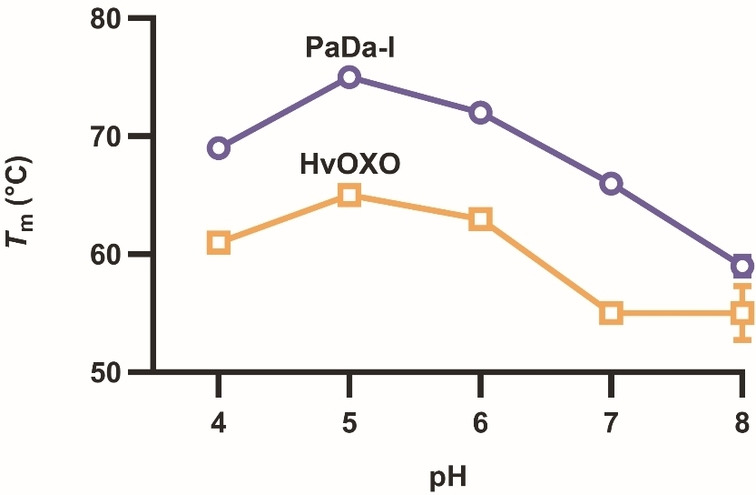
*T*_m_ values of PaDa‐I (○) and HvOXO (□).

**Table 1:** Steady‐state kinetic parameters of HvOXO using oxalate.^[a]^

pH	*k*_cat_ [s^−1^]	*K*_m_ [mM]	*k*_cat_/*K*_m_ [mM^−1^ s ^−1^]
3	0.57±0.03	0.09±0.02	6.33±1.45
4	1.52±0.05	0.73±0.09	2.08±0.27
5	1.87±0.04	22.00±1.67	0.09±0.01

[a] Reactions contained 0.025 μM HvOXO, 0.125 μM PaDa‐I, 0.03–200 mM oxalate, 0.07 mM MBTH and 1 mM DMAB in air‐saturated 100 mM buffer at 25 °C.

Reactions containing PaDa‐I, HvOXO and oxalate were first tested using ethylbenzene (**1**) as a model substrate, which gives the products (*R*)‐1‐phenylethanol (**2**) and acetophenone (**3**)^[17]^ (Figure 2). Enantioselectivity was not investigated for this reaction, since it did not change using diverse H_2_O_2_‐sources in previous studies (Table S1). In parallel, the drug tolmetin (**4**) was assayed as a PaDa‐I substrate to start investigating the potential of combining these enzymes for late‐stage functionalisation of bioactive compounds, since there is a considerable interest in using enzyme‐based C−H diversification strategies to increase the efficiency of drug discovery processes.^[18]^ Full conversion after 24 h was observed for the ethylbenzene, while almost 25 % conversion was achieved with tolmetin (**4**). The TON_UPO_ for ethylbenzene (37 780) slightly improved over other oxidase‐based H_2_O_2_‐generation systems (Scheme 2).

**Figure 2:**
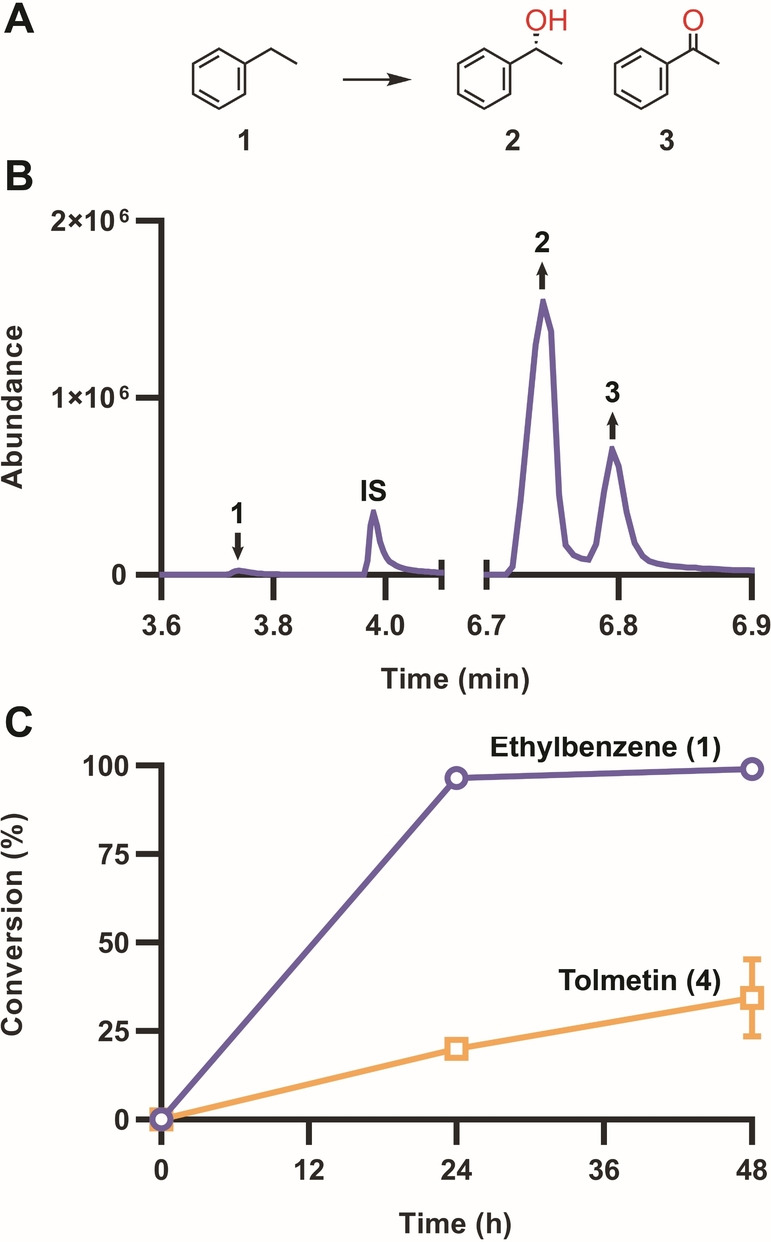
PaDa‐I‐catalysed conversion of ethylbenzene (A–C, ○) and tolmetin (C, □). Reactions (100 μL) contained 1 μM PaDa‐I, 1 μM HvOXO, 50 mM ethylbenzene or tolmetin, 200 mM oxalate, 200 mM buffer at pH 5.0. GC‐MS (ethylbenzene) and UPLC‐MS (tolmetin) analyses after 24 (B) and 48 h at 1000 rpm. Reactions with ethylbenzene and tolmetin contained 10 and 5 % acetonitrile, respectively. 100 % dioxygen gas was blown for 5 min in the empty 3 mL vials, placed on ice, before the addition of the reaction components. **IS**, internal standard. Figure 3 shows the structure of **4**.

These results encouraged us to scale up the tolmetin (**4**) conversion (from 1.6 mg/100 μL to 50 mg/7.5 mL). The resulting products **5**, **6** and **7** (Figure 3) were separated by preparative HPLC and identified by NMR (Figure S13–15, Section S1.13 and S2.2). In parallel, an identical reaction was performed with a cross‐linked enzyme aggregate containing both PaDa‐I and HvOXO (combi‐CLEA, Figure 3 and S16–17, Section S1.12–13) instead of using the soluble enzymes. 84 and 100 % isolated yield was achieved for the reaction containing the soluble enzymes (TON_UPO_ 25 664) and the combi‐CLEA (TON_UPO_ 30 699), respectively. In the case of the combi‐CLEA reaction, enzymes were active for a longer time since mainly product **7** was observed after the same incubation time (96 h), and the negligible amounts of other minor products were not isolated. In the case of the reaction with soluble enzymes, the isolated yield for **5**, **6** and **7** was 16, 24 and 44 %, respectively.

**Figure 3:**
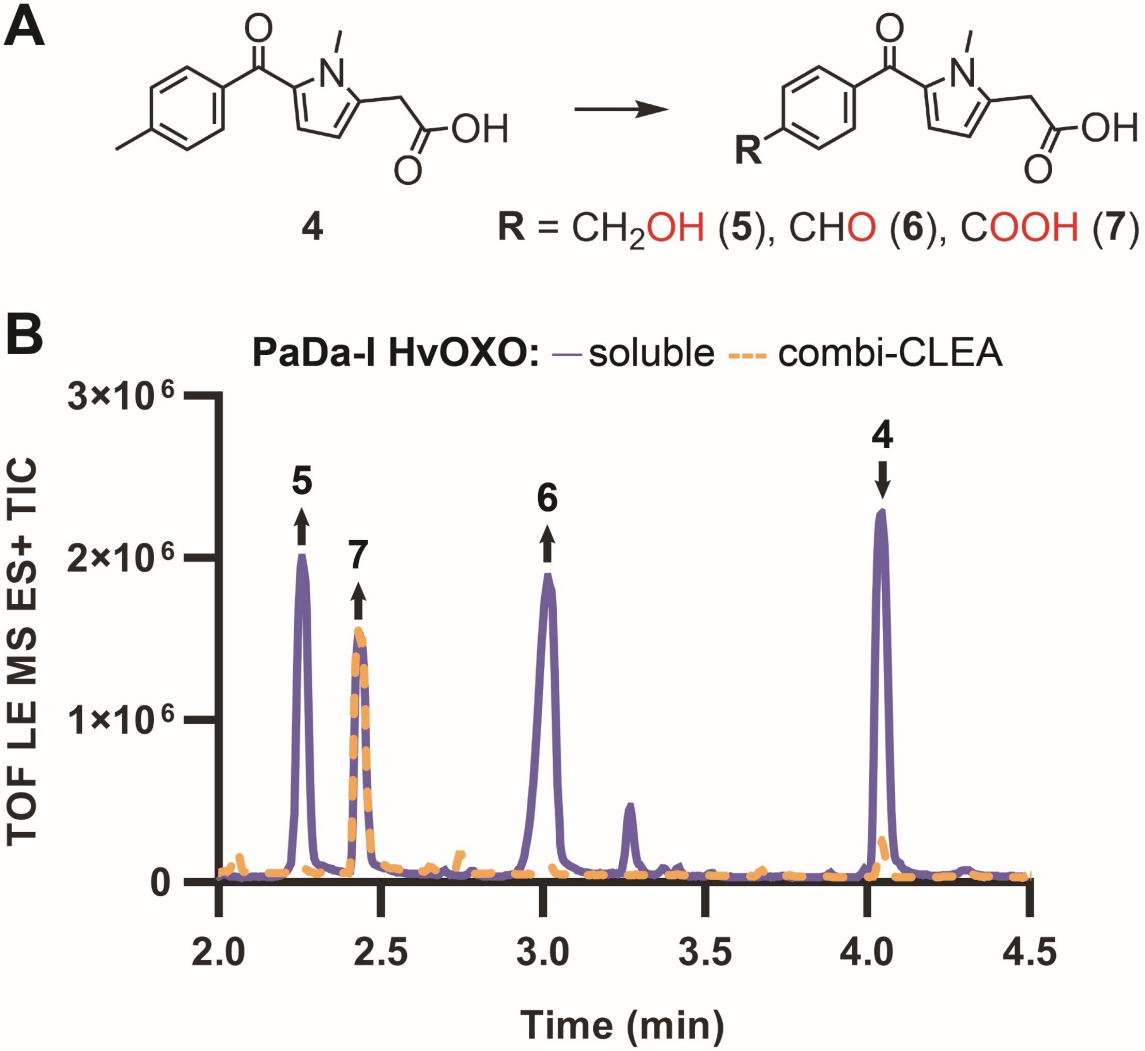
PaDa‐I‐catalysed conversion of 50 mg tolmetin (26 mM) with HvOXO. Reactions (7.5 mL, A) contained 0.8 μM PaDa‐I, 0.8 μM HvOXO, 168 mM oxalate, buffer at pH 5.0. UPLC‐QTOF/MS^E^ analyses after 96 h (B).

Subsequently, the drug tolmetin (**4**) was used as a model substrate to study the influence of various parameters on μL‐scale reactions containing PaDa‐I and HvOXO. First, increasing HvOXO concentrations were tested (Figure S7–8). At pH 3.0 and 4.0, an equimolar concentration of HvOXO and PaDa‐I was optimal. Contrarily, a 3‐fold higher HvOXO:PaDa‐I ratio was required at pH 5.0 to obtain the same conversion as that observed for the reactions containing 10 mM H_2_O_2_ instead of HvOXO and oxalate. This is due to the fact that HvOXO exhibits a high *K*_m(oxalate)_ at pH 5.0 (Table 1) and thus higher oxalate concentrations are recommended at this pH.

Next, we used an equimolar concentration of HvOXO and PaDa‐I at pH 4.0 to further study the efficiency of their partnership. Reactions containing low tolmetin and H_2_O_2_ concentrations (without HvOXO) led to full conversions after 2 h (Figure S9A). The same outcome was observed when H_2_O_2_ was replaced with HvOXO and oxalate, only with extended incubation times due to the slow release of H_2_O_2_. However, the HvOXO‐oxalate system is advantageous for reactions which contain higher tolmetin concentrations (Figure 4). A single addition of an equivalent H_2_O_2_ concentration (10 mM) resulted in around 2.6‐fold less product than when using HvOXO after 20.5 h. Only in the case of reactions with a single addition of H_2_O_2_, were conversions similar after 2 and 20.5 h due to PaDa‐I inactivation (Figure S9B).

**Figure 4:**
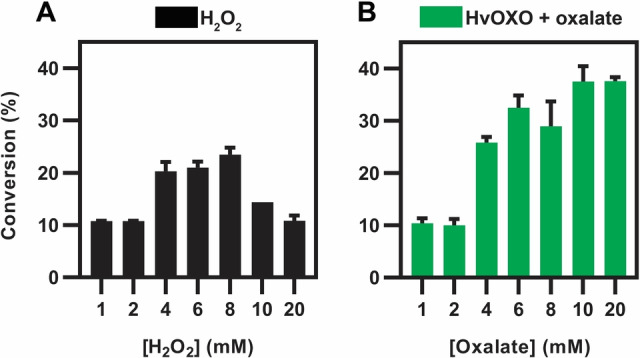
PaDa‐I‐catalysed conversion of 10 mM tolmetin (**4**) with H_2_O_2_ or HvOXO. Reactions (5 μL) contained 0.1 μM PaDa‐I, 0 (A) or 0.1 (B) μM HvOXO, 1–20 mM H_2_O_2_ (A) or oxalate (B), buffer at pH 4.0 and 24 % dimethyl sulfoxide. UPLC‐MS analyses after 20.5 h.

The influence of twelve co‐solvents on tolmetin conversion were also investigated, since they are often needed for substrate solubilization. In reactions with 10 % co‐solvent and HvOXO, conversions were higher than 75 % in the presence of glycerol, acetonitrile, acetone or tetrahydrofuran (Figure 5). In reactions with 25 % co‐solvent (Figure S10B), only glycerol was well‐tolerated by HvOXO. In contrast, reactions initiated by a single H_2_O_2_ addition were not influenced by 25 % acetonitrile, which was the best cosolvent for PaDa‐I. Tolmetin and other drugs exhibit low solubility in glycerol, while acetone is very volatile. Thus, the best option for reactions with both PaDa‐I and HvOXO is 10 % acetonitrile, and 10 % tetrahydrofuran is a good alternative. Similarly, we tested various temperatures (25–50 °C) for the reactions containing these enzymes, which indicated that a temperature of 25 °C is optimal (Figure S11).

**Figure 5:**
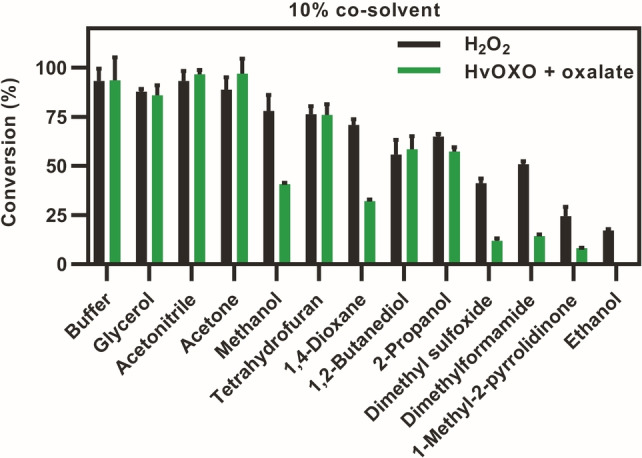
Influence of co‐solvents on PaDa‐I reactions with H_2_O_2_ or HvOXO. Reactions (5 μL) contained 0.1 μM PaDa‐I, 0 or 0.1 μM HvOXO, 0.5 mM tolmetin, 2 mM oxalate or H_2_O_2_, buffer at pH 4.0. UPLC‐MS analyses after 20.5 h.

Subsequently, acetonitrile was selected for a more difficult challenge with up to 100 mM tolmetin. Results using HvOXO with 10–100 mM oxalate were compared to those for the same reactions with 10–100 mM H_2_O_2_ (Figure 6 and S12). As expected, PaDa‐I was rapidly inactivated at high H_2_O_2_ concentrations. There was not a correlation between the H_2_O_2_ concentration and the amount of compound **6** and **7**, which indicated that the conversion of **5** and **6** are UPO‐catalysed instead of H_2_O_2_‐catalysed. In reactions containing 100 mM both tolmetin and oxalate, a TON_UPO_ of 71 377 was determined after 20.5 h. The outcome of these experiments clearly demonstrated the benefits of using HvOXO when high substrate loadings are required.

**Figure 6:**
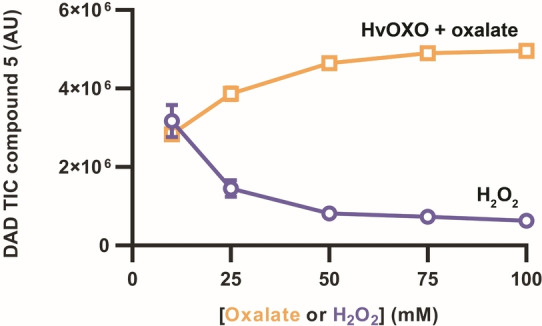
PaDa‐I‐catalysed conversion of 100 mM tolmetin (**4**) with H_2_O_2_ or HvOXO. Reactions (5 μL) contained 0.1 μM PaDa‐I, 0 or 0.1 μM HvOXO, buffer at pH 4.0 and 11 % acetonitrile. UPLC‐MS analyses after 20.5 h. Peak areas for compound **5** are plot. Those for compound **6** are shown in Figure S12.

After performing the reaction optimization, we used the best conditions to demonstrate the ability of PaDa‐I to catalyse late‐stage oxidations of a diverse drug panel using the HvOXO‐oxalate system in 5 μL reactions. 33 out of 64 drugs were used as a substrate by PaDa‐I, with a conversion higher than 70 % for 11 drugs (Figure S18–19). The corresponding UPLC‐QTOF/MS^E^ data were used to tentatively identify major reaction products for various high conversion reactions (Figure 7 and Section S2.3). In the case of empagliflozin (**8**), oxidation on the tetrahydrofuran ring likely produced compound **9** which was followed by ring opening to form a carboxylic acid product (**10**). PaDa‐I presumably accomplished the aromatic mono‐hydroxylation of methotrimeprazine (**11**) and triflupromazine (**13**), while this enzyme found two oxidation sites on the indole ring of fluvastatin (**15**). Phenylbutazone (**17**) was likely hydroxylated by PaDa‐I on the butyl side chain (**18**). In the clozapine (**19**) reaction, compounds **20** and **21** were tentatively assigned as the major reaction products. Using 5‐benzyloxygramine (**22**) as a PaDa‐I substrate, formation of a product with an aliphatic *N*‐oxide (**23**) likely occurred. In the ketoconazole (**24**) reaction, one of the main products was *O*‐dealkylated ketoconazole (**25**). In the case of raloxifene (**26**), our results suggested that PaDa‐I peroxidative activity (Scheme S1) may yield substrate radicals which were subjected to non‐enzymatic formation of a covalent raloxifene homodimer (**27**). Nevertheless, dimerization site on raloxifene was not unequivocally identified. These results confirm the extraordinary ability of PaDa‐I to enable various types of oxygenation reactions and one‐electron oxidations of non‐native substrates.^[19]^

**Figure 7:**
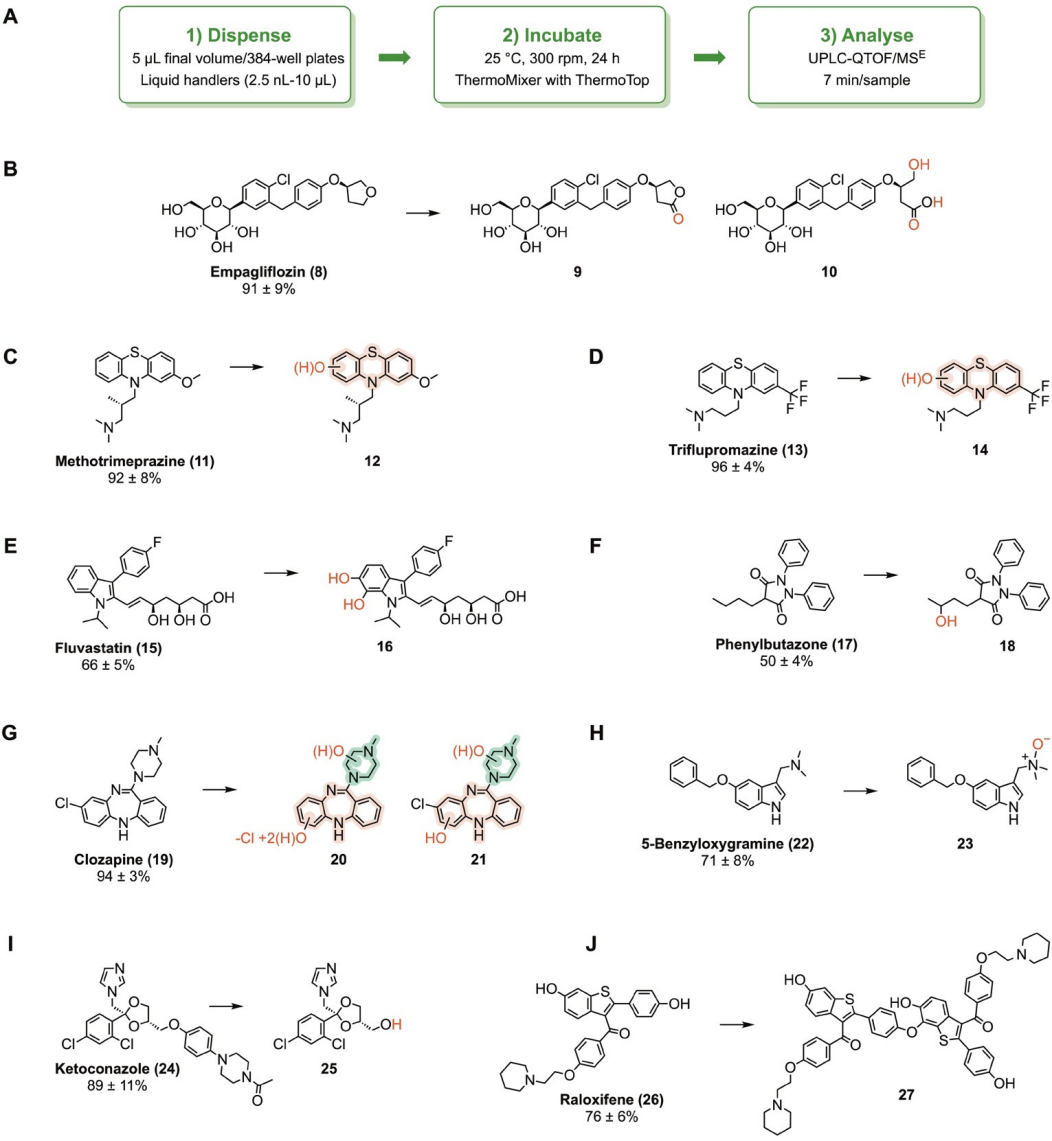
Examples of drug substrates and products tentatively identified in PaDa‐I reactions using UPLC‐QTOF/MS^E^ data (Section S2.3) and available literature. All reactions (5 μL) contained 0.8 μM PaDa‐I, 0.8 μM HvOXO, 0.5 mM drug, 10 mM oxalate, buffer at pH 4.0, 2.5 % either acetonitrile or tetrahydrofuran (Table S2). Conversions (%) after 24 h are indicated. A) Workflow. B–D and G–I) Reaction products with a satisfactory assignment are depicted. E) Functionalization sites on the indole ring of fluvastatin (**15**) were not unequivocally identified. 5‐Hydroxy‐ and 6‐hydroxy‐fluvastatin are main products in human liver microsomes.^[22]^ F) It was not unequivocally determined which carbon was hydroxylated by PaDa‐I on the phenylbutazone (**17**) butyl side chain. We are depicting a major phenylbutazone derivative resulting from human metabolism.^[23]^ J) Dimerization site on raloxifene (**26**) was not unequivocally identified. Herein, a raloxifene dimer (**27**) produced by CYP3 A4 is shown as an example. In this case, a 1‐electron oxidation of the raloxifene 4‐hydroxyphenyl moiety took place to form an oxygen‐centered radical. Another raloxifene molecule was oxidized on the benzo[*b*]thiophen‐6‐ol moiety to form an oxygen‐centered radical which converted into the position 7 carbon‐centered radical after delocalization. Non‐enzymatic coupling of these radicals yielded the dimer.^[24]^

In summary, we have demonstrated here that HvOXO can play an important role as an H_2_O_2_‐source in UPOs‐catalysed oxyfunctionalisations using an inexpensive sacrificial electron donor. Furthermore, this work showed for the first time that it is possible to do high‐throughput screening using 5 μL‐scale UPO reactions by converting 33 drugs. One example reaction was successfully scaled up to 50 mg and featured a synthetically useful oxidation of tolyl methyl to carboxylic acid. Libraries of drug analogues resulting from miniaturised assays are useful assets for bioactivity screening and in the synthesis of human drug metabolites. For example, different non‐purified μL‐scale UPO reactions may be subjected to biophysical methods such as surface plasmon resonance (SPR)^[20]^ in order to rapidly discover compounds displaying potent binding affinities to target proteins. These state‐of‐the‐art approaches fulfil “the need for speed”^[21]^ which is demanded by pharmaceutical companies, while contributing to the urgent transition to a sustainable economy by reducing wastes and consumption of reactants and organic solvents.

## Conflict of interest

The authors declare no conflict of interest.

## Supporting information

As a service to our authors and readers, this journal provides supporting information supplied by the authors. Such materials are peer reviewed and may be re‐organized for online delivery, but are not copy‐edited or typeset. Technical support issues arising from supporting information (other than missing files) should be addressed to the authors.

Supporting Information

## Data Availability

The data that support the findings of this study are available in the supplementary material of this article.
