# Supplementary material for: Oxalate Oxidase for In Situ H2O2‐Generation in Unspecific Peroxygenase‐Catalysed Drug Oxyfunctionalisations
Source: Angew Chem Int Ed Engl. 2022 Aug 25;61(39):e202207831. doi: 10.1002/anie.202207831 (PMC9805127; doi:10.1002/anie.202207831)
Supplement: Supplementary file 1 — Supporting Information [file ANIE-61-0-s001.pdf]

Angewandte Chemie
Eine Zeitschrift der Gesellschaft Deutscher Chemiker

Supporting Information

Oxalate Oxidase for In Situ H_2O_2-Generation in Unspecific Peroxygenase-Catalysed Drug Oxyfunctionalisations

*E. Romero, M. J. Johansson, J. Cartwright, G. Grogan, M. A. Hayes**

Table of Contents

S1. Experimental Procedures

S1.1. Preparation of DNA constructs
S1.2. Yeast fermentation
S1.3. Enzyme purification
S1.4. Determination of enzyme concentration
S1.5. Steady-state kinetics
S1.6. Melting temperature
S1.7. Long-term stability
S1.8. General indications for the reactions in microplates
S1.9. Work-up and ultrahigh performance liquid chromatography-mass spectrometry (UPLC-MS) for drug-containing reactions
S1.10. Work-up and gas chromatography mass-spectrometry (GC-MS) for ethylbenzene-containing reactions
S1.11. Co-solvent stock preparation for testing their influence on tolmetin conversions
S1.12. Preparation of cross-linked enzyme aggregates which contain two enzymes (combi-CLEA)
S1.13. Preparation of tolmetin-containing reactions on a 50-mg scale and work-up for product isolation and identification
S1.14. Work-up and ultra-performance liquid chromatography coupled with electrospray ionization quadrupole time-of-flight mass spectrometry operating in MS^E mode (UPLC-QTOF/MS^E) for drug-containing reactions

S2. Results and Discussion

S2.1. Figures

Figure S1. Standard curves obtained by GC-MS
Figure S2. Molecular weight (**A**) and absorption spectrum (**B**) of purified PaDa-I and HvOXO
Figure S3. Melting temperature curves of PaDa-I (**A**) and HvOXO (**B**) at pH 3.0-8.0
Figure S4. Long-term stability of PaDa-I (**A**) and HvOXO (**B**) at pH 3.0 (△), 4.0 (◘) and 5.0 (◉)
Figure S5. Oxalate oxidation rates in reactions catalysed by HvOXO at various pH values
Figure S6. Crystal structure of recombinant HvOXO monomer in complex with the substrate analogue glycolate (PDB ID: 2ETE)
Figure S7. Influence of HvOXO:PaDa-I ratio on tolmetin (**4**) conversion at pH 3.0 (△), 4.0 (◘) and 5.0 (◉)
Figure S8. Peak areas corresponding to compound **5** (◘) and **6** (◉) which result from the reaction of PaDa-I with tolmetin (**4**) in the absence or the presence of an increasing HvOXO concentration at pH 3.0 (**A**), 4.0 (**B**) and 5.0 (**C**)
Figure S9. PaDa-I-catalysed conversion of 0.5 (**A**) and 10 (**B**) mM tolmetin with H_2O_2 or HvOXO
Figure S10. Influence of co-solvents on PaDa-I-catalysed tolmetin conversion with either H_2O_2 or HvOXO
Figure S11. Influence of temperature (**A**-**D**) and mixing frequency (**C**-**D**) on the conversion of tolmetin catalysed by PaDa-I in the presence (**A**-**D**, ◘, ■) and the absence (**A**-**B**, ◉) of HvOXO
Figure S12. PaDa-I-catalysed conversion of 100 mM tolmetin with H_2O_2 or HvOXO
Figure S13. HPLC chromatograms of the reaction containing 50 mg tolmetin and soluble PaDa-I and HvOXO
Figure S14. UPLC-QTOF analyses of the products purified from the reaction containing 50 mg tolmetin and soluble PaDa-I and HvOXO
Figure S15. UPLC-QTOF analyses of the fractions 1-2 which were obtained during the HPLC analyses shown in **Fig. S13D**
Figure S16. HPLC chromatograms of the reaction containing 50 mg tolmetin and combi-CLEA PaDa-I and HvOXO
Figure S17. UPLC-QTOF analyses of the products purified from the reaction containing 50 mg tolmetin and combi-CLEA PaDa-I and HvOXO
Figure S18. Drugs converted by PaDa-I
Figure S19. Drugs not converted by PaDa-I

S2.2. NMR spectral data

S2.2.1. Tolmetin (**4**) [2-(1-methyl-5-(4-methylbenzoyl)-1*H*-pyrrol-2-yl)acetic acid]
S2.2.2. 2-(5-(4-(hydroxymethyl)benzoyl)-1-methyl-1*H*-pyrrol-2-yl)acetic acid (**5**)
S2.2.3. 2-(5-(4-formylbenzoyl)-1-methyl-1*H*-pyrrol-2-yl)acetic acid (**6**)
S2.2.4. 4-(5-(carboxymethyl)-1-methyl-1*H*-pyrrole-2-carbonyl)benzoic acid (**7**)

S2.3. UPLC-QTOF data obtained from the 64-panel drug high-throughput screening (Fig. S18)

S2.3.1. Empagliflozin (**8**)
S2.3.2. Methotrimeprazine (**11**)
S2.3.3. Triflupromazine (**13**)

S2.3.4. Fluvastatin (**15**)
S2.3.5. Phenylbutazone (**17**)
S2.3.6. Clozapine (**19**)
S2.3.8. Ketoconazole (**24**)
S2.3.9. Raloxifene (**26**)

S2.4. Scheme

Scheme S1. Catalytic cycle of UPOs

S2.5. Tables

Table S1. Comparation of H_2O_2-generation systems for ethylbenzene hydroxylation catalysed by AaeUPO
Table S2. Drug stocks prepared for 5 µL-scale reactions (**Fig. S18-19**)

S3. References

S4. Author Contributions

S1. Experimental Procedures

S1.1. Preparation of DNA constructs

Electra reagents kit (ATUM, including type IIS restriction enzyme SapI) was used for cloning in either pD902 or pD912 vectors (ATUM). *Agrocybe aegerita* unspecific peroxygenase (AaeUPO) gene (GenBank: FM872458.1) containing nine mutations (PaDa-I)[1] was kindly provided by Prof. Gideon Grogan (York University, UK). This gene was provided in the pPICZα B vector (Invitrogen), which was replaced with the pD902 vector for our work (after performing one silence mutation in the gene to remove SapI restriction site by QuikChange). The resulting construct contains the methanol-inducible AOX1 promotor and the secretion signal of PaDa-I AaeUPO. *Hordeum vulgare* (barley) oxalate oxidase (HvOXO) gene (GenBank ID: L15737.1) was optimized for *Komagataella phaffii* (*Pichia pastoris*) expression and synthesized as an Invitrogen GeneArt Strings DNA fragment (ThermoFisher Scientific). The signal sequence to direct secretion of HvOXO to the cell wall was not included in the DNA synthesized fragment. The mature sequence of HvOXO (GeneBank ID: AAA32959.1) was followed by the recognition site (ENLYFQG) for tobacco etch virus protease (TEV) and a 6xHis-tag. The double-stranded DNA fragment was cloned in the pD912 vector. This vector contains the AOX1 promotor and the secretion signal of *Saccharomyces cerevisiae* mating factor α-1. The optimized sequence of HvOXO-TEV-His-tag, which is not available in the databases, is shown below:

> <u>HvOXO-TEV-His-tag</u>
TCTGATCCAGATCCTCTGCAAGATTTCTGTGTCGCTGATTTGGACGGTAAGGCCGTTTCTGTTAACGGTCACACTTGTAAGCCAA
TGTCTGAAGCTGGTGACGACTTCCTGTTCTCCTCAAAGTTGACTAAGGCTGGTAACACCTCCACTCCAAACGGTTCTGCTGTTAC
TGAATTGGACGTTGCCGAATGGCCTGGAACTAACACTTTGGGTGTTTCCATGAACAGAGTCGACTTTGCTCCAGGTGGTACTAAT
CCACCACACATTCATCCAAGAGCTACCGAGATCGGTATGGTCATGAAGGGTGAGTTGTTGGTCGGTATCTTGGGTTCTTTGGACT
CCGGTAACAAGCTGTACTCCAGAGTTGTTAGAGCCGGTGAGACTTTCGTTATCCCAAGAGGTTTGATGCACTTCCAGTTCAACGT
TGGTAAGACCGAGGCCTACATGGTTGTGTCCTTCAACTCTCAAAACCCCGGTATCGTTTTCGTCCCATTGACTTTGTTTGGTTCTG
ACCCACCAATTCCTACTCCAGTTTTGACCAAGGCTTTGAGAGTCGAGGCTGGTGTTGTTGAATTGCTGAAGTCTAAGTTCGCTGG
TGGTTCCGAGAACTTGTACTTTCAAGGTCATCACCACCACCATCACTAA

S1.2. Yeast fermentation

PaDa-I AaeUPO and HvOXO were expressed using *K. phaffii* as a host (PPS-9010 ATUM) in a 5 L fermentor (B. Braun Biotech International). Plasmid transformation and screening for expression were performed following standard procedures described in ATUM website. The presence of the expression cassette in the yeast genome was confirmed by colony PCR using the Phire Plant Direct PCR master mix (Thermo Scientific) followed by DNA sequencing. To obtain a preculture, a few single colonies were used to inoculate 250 mL of buffered yeast extract peptone dextrose medium (YPD with 200 mM potassium phosphate buffer pH 6.0). The incubation (in a 2.5 L baffled flask) was performed for around 65 h at 30 ºC and 150 rpm (Infors HT Multitron Standard incubator shaker). Before the inoculation with 200 mL preculture, the fermentor contained 3 L basal salts medium (BSM, 1.7 mM $CaSO_4 \cdot 2H_2O$, 26.2 mM K_2SO_4, 15.1 mM $MgSO_4 \cdot 7H_2O$, 68.1 mM $(NH_4)_2SO_4$, 12 g/L sodium hexametaphosphate, 4.5% glycerol (v/v) and 4 mL/L PMT1 solution; PMT1 solution: 24.0 mM $CuSO_4 \cdot 5H_2O$, 0.5 mM NaI, 17.7 mM $MnSO_4 \cdot H_2O$, 0.8 mM $Na_2MoO_4 \cdot 2H_2O$, 0.3 mM H_3BO_3, 2.1 mM $CoCl_2 \cdot 6H_2O$, 146.7 mM $ZnCl_2$, 233.8 mM $FeSO_4 \cdot 7H_2O$, 0.8 mM biotin and 51.0 mM H_2SO_4). Initial fermentation was carried out at 30 ºC, 30% pO_2 (cascade mode, varying stirring and air flow) and pH 5.0 (controlled using 30% ammonia in water). After glycerol depletion

(~20 h), a glycerol feed (5-20% flow, 1.6 mm inner diameter tubing) was performed until the cell density reached approximately 200 g/L. Next, temperature and glycerol feed were gradually decreased to 20 ºC and 0% (~1 h), 12 mL of a 234 mM $FeSO_4 \cdot 7H_2O$ solution were added to the fermentor and then 43 h of methanol feed were performed (flow, 1.6 mm inner diameter tubing: 2% 6 h, 4% 12 h, 6% 2 h, 7% 2 h, 8% 2 h, 9% 19 h). 1-2 mL antifoam (Struktol J673A) were added each fermentation per day. After fermentation, cells were removed by centrifugation (45 min, 4 ºC, 12,000 x g) and the supernatant (~3 L) was concentrated (~0.250 L) using a tangential flow filtration system (Cogent M, Mettler Toledo, 10 kDa MWCO). Two tablets of protease inhibitor cocktail (cOmplete, EDTA-free, Roche) were added to the concentrated sample.

S1.3. Enzyme purification

Purification of PaDa-I involved both hydrophobic interaction and anion exchange chromatographies using an ÄKTA fast protein liquid chromatography (FPLC) system (Amersham Pharmacia Biotech). First, 40% ammonium sulfate saturation of the sample was achieved by slow addition of solid ammonium sulfate at 4 ºC under continuous stirring (59 g/250 mL). After centrifugation (1 h, 4 ºC, 72,500 x g), the supernatant was subjected to vacuum filtration (0.45 µm PES filter unit, VWR) and applied to three HiTrap Phenyl HP 5 mL connected in series (GE Healthcare). The column equilibration buffer was 20 mM sodium phosphate pH 7.0 containing 1.8 M ammonium sulfate. After loading the sample (5 mL/min), column was washed with five volumes of equilibration buffer and five volumes of the same buffer containing 0.9 M ammonium sulfate. Finally, PaDa-I elution was performed using the same buffer without ammonium sulfate. Next, the resulting PaDa-I sample was concentrated to 15 mL using centrifugal devices (10 kDa MWCO, Pall Macrosep Advance, 4,000 x g, 4 ºC), followed by buffer exchange using a HiPrep 26/10 desalting column (GE Healthcare) equilibrated with 20 mM TrisHCl pH 7.0. Finally, purification of PaDa-I was accomplished by using a RESOURCE Q column (6 mL, GE Healthcare). Mobile phases were 20 mM TrisHCl pH 7.0 and the same buffer with 2 M NaCl. Protein was eluted from the column using a linear salt gradient (0-500 mM NaCl, 70 min, 1 mL/min). Fractions having a Reinheitzahl (RZ) value (A418/A280) of 1.8-2.2 were pooled, concentrated using centrifugal devices and subjected to buffer exchange using a PD-10 desalting column (Amersham Biosciences) equilibrated with 50 mM potassium phosphate pH 7.0. Purified PaDa-I was flash frozen and stored at -80 ºC.

In the case of the His-tagged HvOXO, gravity-flow bench purification was carried out using Ni-sepharose 6 Fast Flow (GE Healthcare, 3 mL) and standard protocols. 20 mM sodium phosphate pH 7.4 with 0.5 M NaCl was used, which contained 0, 5, and 500 mM imidazole for equilibration, wash and elution, respectively. Before storing the purified samples at -80 ºC, buffer exchange of HvOXO was carried out using a PD-10 desalting column equilibrated with 100 mM citrate-phosphate buffer at pH 4.0.

S1.4. Determination of enzyme concentration

Purified enzyme concentration was determined based on the extinction coefficient at 280 nm: 134.0 $mM^{-1}cm^{-1}$ for PaDa-I and 76.4 $mM^{-1}cm^{-1}$ for HvOXO. To determine these values according to the Beer-Lambert law, various enzyme dilutions were prepared. Next, their absorbance at 280 nm was measured using a NanoDrop spectrophotometer (ND-1000), while the protein concentration was determined using the Bradford reagent (Sigma-Aldrich).

S1.5. Steady-state kinetics

Steady-state kinetic parameters for HvOXO were determined using potassium oxalate as a substrate, a Varian Cary 300 Bio UV-Visible spectrophotometer and the following PaDa-I-coupled assay. Reactions (100 µL) contained 0.025 µM HvOXO, 0.125 µM PaDa-I, 0.03-200 mM potassium oxalate, 0.07 mM 3-methyl-2-benzothiazolinone hydrazone (MBTH; 1.4 mM stock prepared in water) and 1 mM 3-(dimethylamino)benzoic acid (DMAB; 20 mM stock prepared in methanol) in air-saturated 100 mM citrate-phosphate pH 3.0, 4.0 or 5.0 at 25 ºC. PaDa-I catalyzes the oxidative coupling of MBTH and DMA to form an indamine dye, which exhibits an absorption maximum at 590 nm ($\mathcal{E}_{590}$ 53 $mM^{-1}cm^{-1}$).[2] 1 µmol of indamine dye is formed per 1 µmol of H_2O_2 which is produced by HvOXO as a result of the reaction of this enzyme with 1 µmol of potassium oxalate and dioxygen. Potassium oxalate stocks were prepared in 100 mM citrate-phosphate buffer (20 mM at pH 3.0 and 4.0 and 1 M at pH 5.0). pH of the potassium oxalate stocks was adjusted before adjusting the volume.

S1.6. Melting temperature

Melting temperature values of PaDa-I and HvOXO were determined using a LightCycler® 480 II System (Roche Applied Science) and 384-well PCR plates with qPCR adhesive seals (4titude FrameStar). 4 µL of the enzyme stock prepared in 50 mM potassium phosphate buffer pH 7.0 were mixed with 21 µL 100 mM citrate-phosphate at pH 3.0-8.0. Two 10 µL aliquots of each solution were pipetted into the 384-well plate. Next, 30 nL of 10 mM SYPRO Orange Protein Gel Stain (Sigma-Aldrich, in DMSO) were added using a HP D300 Digital Dispenser to each 10 µL sample. Final concentrations were 0.872 µM PaDa-I (31 µg/mL) or 2.388 µM HvOXO (55 µg/mL), 30 µM SYPRO Orange and 0.3% dimethyl sulfoxide. Fluorescence was measured during enzyme denaturation by increasing the temperature from 20 to 98 °C.[3] Light Cycler 480 SW 1.5.1 software was used to calculate the negative first derivatives (-dF/dt), which reveals melting temperatures as peaks.

S1.7. Long-term stability

Long-term stability was determined for PaDa-I and HvOXO at pH 3.0, 4.0 and 5.0 at 25 ºC. Enzyme stocks (0.5 µM, 100 µL in a 1.5 mL tube) were incubated in a ThermoMixer with ThermoTop (Eppendorf). Aliquots (5 µL) of these solutions were withdrawn after 24 and 48 h of incubation to determine residual enzyme activity using a Varian Cary 300 Bio UV-Visible spectrophotometer at 25 ºC. Residual peroxygenase activity of PaDa-I was determined by following the conversion of 5-nitro-1,3-benzodioxole (NBD, 10 mM stock prepared in acetonitrile) to 4-nitrocatechol (ε_{425} = 9700 M^{-1} cm^{-1}). Reactions (100 µL) contained 1 mM NBD, 2 mM H_2O_2 and 0.025 µM

PaDa-I in 100 mM potassium phosphate pH 7.0. Residual activity of HvOXO on 5 mM potassium oxalate was determined by following the oxidative coupling of MBTH and DMA as described in **Section S1.5** at pH 4.0.

S1.8. General indications for the reactions in microplates

All 5 µL reactions were performed in a 384-well low dead volume microplate (Echo qualified, Labcyte) covered with a sealing film (Axygen AxySeal, PCR-SP). Unless stated otherwise, all incubations were performed at 25 °C and 300 rpm in a ThermoMixer with ThermoTop (Eppendorf). 100 mM citrate-phosphate was used as a buffer. A small percentage of an organic solvent (v/v) was used as a cosolvent for substrate solubilization or for investigating enzyme stability as indicated for each experiment. Echo 655 (Labcyte) was used to dispense aqueous solutions or dimethyl sulfoxide. Mosquito HV (sptlabtech) was used to dispense other organic solvents. Reaction product analyses were performed by UPLC-MS or UPLC-QTOF/MS^E as described in **Section S1.9** and **S1.14**, respectively. Conversions are relative to the initial substrate concentration, unless stated otherwise, and based on two or three replicates.

S1.9. Work-up and ultrahigh performance liquid chromatography-mass spectrometry (UPLC-MS) for tolmetin-containing reactions

PaDa-I-catalysed 5 µL-scale conversions of tolmetin (final concentration of 0.5, 10 or 100 mM) were stopped by adding 1 µL 3 M HCl and 6 µL dimethyl sulfoxide using an Echo 655 liquid dispenser. After mixing for 5 min at 750 rpm (Heidolph Microtiter Plate Shaker Titramax 1000) and subsequent centrifugation for 1 min at 1500 x g (Eppendorf 5810 R), the supernatant was transferred from the reaction plate (384-well low dead volume microplate, Echo qualified, Labcyte) to the analysis plate (384-well, PP, small volume, deep well, Greiner bio-one) using a Mosquito HV liquid handler. After sealing the plate using a thermal heat sealer (Velocity11´s PlateLoc), 5 µL sample were injected in a Waters Acquity UPLC equipped with a photodiode array (PDA) detector, a 3100 mass spectrometer and an Acquity UPLC BEH C18 column (1.7 µm, 2.1 x 50 mm). To prepare the mobile phases A and B, 4 mL of a basic stock solution (1.625 M NH_4HCO_3 and 11.269 M NH_3 in water) were added to 1 L water (A) and to 1 L 95% acetonitrile in water (B). Method was: 10%B 0.2 min, 10-99%B 1.5 min, and 99-10%B 0.01 min. Flow rate was 1 mL/min.

In the case of 100 µL-scale conversions of 50 mM tolmetin in a 3 mL vial (**Fig. 2**), reactions were stopped by adding 100 µL 3 M HCl and 600 µL dimethyl sulfoxide to ensure complete solubilization before performing the UPLC-MS analyses described above.

S1.10. Work-up and gas chromatography mass-spectrometry (GC-MS) for ethylbenzene-containing reactions

100 µL-scale conversions of 50 mM ethylbenzene in a 3 mL vial (**Fig. 2**) were extracted with 300 µL ethyl acetate containing 3 mM phenylacetylene as an internal standard. The organic extracts were dried over $MgSO_4$. Subsequent GC-MS analyses were performed using a J&W HP-5ms GC column (30 m, 0.25 mm, 0.25 µm, Agilent 19091S-433) and He gas as the carrier gas (Agilent Technologies 7890A GC system and 5975C Inert MSD detector). Column temperature was: 50 °C for 3 min, increased to 150 °C at a rate of 15 °C/min, increased to 250 °C at a rate of 60 °C/min and 250°C for 2 min. Injection volume was 1 µL with a split ratio of 30:1. Plots for standards are shown in **Fig. S1**.

S1.11. Co-solvent stocks preparation for testing their influence on tolmetin conversions

Increasing amounts of various co-solvents (0, 5, 10 and 25%, v/v) were tested in the PaDa-I reactions with either H_2O_2 or HvOXO and oxalate (**Fig. 5** and **S10**). Co-solvent stocks contained 55% (v/v) organic solvent in 45 mM citrate-phosphate buffer at pH 4.0 (final concentrations). Citrate-phosphate buffer was prepared by mixing appropriate volumes of 0.1 M citric acid and 0.2 M disodium hydrogen phosphate as indicated in a previous publication.[4] After mixing the buffer with the organic solvent, the pH was adjusted to 4.0 before adjusting the volume with water. All reactions (5 µL, final volume) contained 0.1 µM PaDa-I, 0.5 mM tolmetin and 100 mM citrate-phosphate buffer pH 4.0. 42 mM tolmetin stock was prepared in dimethyl sulfoxide. Thus, all reactions contained 1% dimethyl sulfoxide in addition to 0-25% of the cosolvent under study.

S1.12. Preparation of cross-linked enzyme aggregates which contain two enzymes (combi-CLEA)

0.4 g ammonium sulfate were weighted out in a 1.5-mL tube. Next, 469 µL purified PaDa-I (13.5 µM stock in 50 mM potassium phosphate pH 7.0) and 96 µL purified HvOXO (66.1 µM stock in 100 mM citrate-phosphate pH 4.0) were added to the same tube (final volume of 740 µL). This mixture was incubated at 1000 rpm and 4 °C for 1 h using a ThermoMixer to achieve protein precipitation. Subsequently, 20 µL glutaraldehyde [25% (w/w) stock in water, Sigma-Aldrich] were added and the resultant mixture (0.7% glutaraldehyde, 8 µM PaDa-I, 8 µM HvOXO in 760 µL) was incubated at 1000 rpm and 4 °C for 2 h to achieve crosslinking of the proteins by glutaraldehyde. Immediately after the incubation, the resultant suspension containing the combi-CLEA was added to a 50-mg tolmetin solution which was prepared as described in **Section S1.13**. Subsequently, the combi-CLEA tube was washed with 1 mL 200 mM citrate-phosphate pH 5.0 and the washing was added to the same reaction. Following this protocol, two control samples were prepared: i) control with PaDa-I without HvOXO; and ii) control without both PaDa-I and HvOXO (*i.e.*, ammonium sulfate, glutaraldehyde and buffer). In these controls, the corresponding buffer was added instead of the enzyme.

In order to compare the performance of the combi-CLEA and soluble enzymes, a solution containing 469 µL purified PaDa-I (13.5 µM stock in 50 mM potassium phosphate pH 7.0) and 96 µL purified HvOXO (66.1 µM stock in 100 mM citrate-phosphate pH 4.0) was prepared in a 1.5-mL tube without ammonium sulfate. This mixture was kept at 4 °C while the combi-CLEA sample was being prepared.

S1.13. Preparation of tolmetin-containing reactions on a 50-mg scale and work-up for product isolation and identification

50.1 mg tolmetin (61.4 mg tolmetin sodium salt dihydrate) were weighted out in a 25-mL flask (Duran). Next, 2.607 mL 200 mM citrate-phosphate pH 5.0 and 3.172 mL potassium oxalate (400 mM stock in 200 mM citrate-phosphate pH 5.0) were added to the flask, which was covered with a rubber stopper (turn-over flange, 30.7 mm, Saint-Gobain Performance Plastics) and incubated for 2 h to ensure maximum solubilization of tolmetin before adding the enzymes (200 rpm, 25 °C, Infors HT Multitron Standard incubator shaker). After the incubation, a solution containing both PaDa-I and HvOXO was added to the flask. Then, 1.196 mL 200 mM citrate-phosphate pH 5.0 were used to wash the enzyme tube and the washing was added to the reaction. Final concentrations in 7.539 mL were: 0.8 µM PaDa-I, 0.8 µM HvOXO, 26 mM tolmetin and 168 mM potassium oxalate. An identical reaction was prepared except for having combi-CLEA (with both PaDa-I and HvOXO) instead of the soluble enzymes, as well as two control reactions. The protocol to prepare the combi-CLEA sample and the controls is detailed in **Section S1.12**. The pH of the reaction with soluble enzymes and combi-CLEA was determined immediately before stopping them after 96 h of incubation (pH 6.24 and 6.58, respectively) to confirm that there was not a major change in pH respect to the initial one (pH 5.0).

Each reaction was stopped by adding 1.5 mL 3 M HCl and 25 mL dimethyl sulfoxide. After centrifugation of the resultant sample (18,210 x g, 22 °C), the supernatant was subjected to reverse phase chromatography (XBridge BEH C18 OBD Prep column, 130 Å, 10 µm, 50 mm x 250 mm, Waters) using a Gilson high-performance liquid chromatography (HPLC) instrument. Mobile phases were 10 mM ammonium bicarbonate (NH_4HCO_3) in H_2O:acetonitrile (95:5, A) and acetonitrile (B). Gradient was: 0-30% B, 20 min (sample with soluble enzymes) and 0-10% B, 30 min (sample with combi-CLEA). Flow rate was 100 mL/min. Resulting fractions with absorbance at 253 nm were analysed by UPLC-QTOF (**Section S1.14**). In view of these analyses, fractions containing the same product were mixed. Fractions containing more than one product or contaminants were again subjected to reverse phase chromatography using a different gradient as indicated in **Fig. S13** and **S16**. Purified compounds were concentrated using a rotary evaporator, transferred to a 30-mL glass vial and dried using a Biotage V-10 Touch instrument. After resuspending in 300-750 µL dimethyl sulfoxide-d6, NMR analyses were performed (**Section S2.2**). In addition, 0.5 mM samples were prepared in 0.1% (v/v) formic acid:acetonitrile (1:2) and injected (4 µL) in the UPLC-QTOF instrument (**Section S1.14**, **Fig. S14-15** and **S17**).

S1.14. Work-up and ultra-performance liquid chromatography coupled with electrospray ionization quadrupole time-of-flight mass spectrometry operating in MS^E mode (UPLC-QTOF/MS^E) for drug-containing reactions

PaDa-I-catalysed 5 µL-scale conversions of a diverse drug panel (final concentration of 0.5 mM, **Fig. S18-19**) were stopped by two sequential 3.5 µL aliquots of acetonitrile using a Mosquito HV liquid handler. After mixing for 15 min at 900 rpm and 25 °C (ThermoMixer with ThermoTop) and subsequent centrifugation for 1 min at 1500 x g (Eppendorf 5810 R), the supernatant was transferred (two sequential 5 µL aliquots) from the reaction plate (384-well low dead volume microplate, Echo qualified, Labcyte) to the analysis plate (384-well, PP, small volume, deep well, Greiner bio-one) using a Mosquito HV liquid handler. Next, the analysis plate was sealed using a thermal heat sealer (Velocity11´s PlateLoc).

Analyses were performed using an Acquity Ultra-Performance Liquid Chromatography (UPLC) system paired with a SYNAPT G2 high definition (HDMS) quadrupole time-of-flight (QTof) mass spectrometer (Waters). Electrospray ionization data were obtained in positive ion mode (ES+). All data were acquired in MS^E mode, which allows the exact mass determination for both the precursor and fragment ions in a single analysis by performing the acquisition at low- and high-energy, respectively. Mobile phases were 0.1% formic acid in water (A) and acetonitrile (B). Samples (1-5 µL) were injected into an Acquity UPLC BEHC18 column (130 Å, 1.7 µm, 2.1 mm x 100 mm, Waters). Gradient was: 10-70% B, 6 min. Flow rate was 0.5 mL/min. Leucine enkephalin was used for lock mass at a concentration of 2 ng/µL in 0.1% (v/v) formic acid:acetonitrile (1:1). Data acquisition and processing were performed using MassLynx v4.2 (Waters) and ACD/Spectrus Processor 2020.2.0 (ACD/Labs).

S2. Results and Discussion

S2.1. Figures

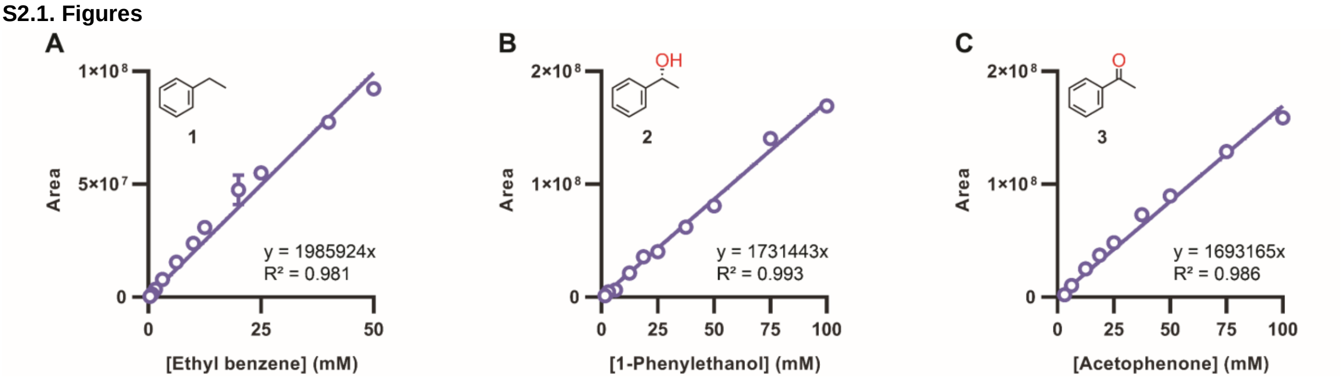

Figure S1. Standard curves obtained by GC-MS. Compounds were prepared in acetonitrile. Values are based on two replicates.

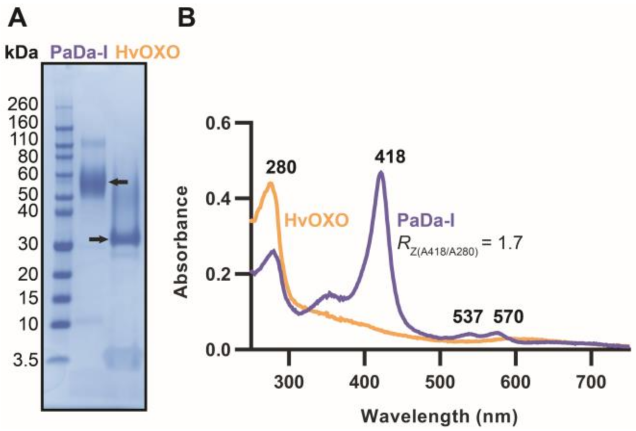

Figure S2. Molecular weight (A) and absorption spectrum (B) of purified PaDa-I and HvOXO. Electrophoresis was performed under denaturing conditions using a NuPAGE Bis-Tris gel (4-12% polyacrylamide, Invitrogen) and Novex Sharp Pre-stained protein standard (Invitrogen). Arrows indicate the protein band corresponding to either PaDa-I or HvOXO. The molecular weight of the glycosylated HvOXO was determined to be 30-33 kDa, based on the electrophoresis performed under denaturing conditions. This analysis indicates that the carbohydrate content of HvOXO is between 29-36%, since a molecular weight of 21.2 kDa was expected for the non-glycosylated monomeric form. Molecular weight, carbohydrate content and spectral properties of our purified PaDa-I are identical to those described in previous studies.[5] Spectra were recorded for 2 µM PaDa-I and 5 µM HvOXO in 50 mM potassium phosphate pH 7.0.

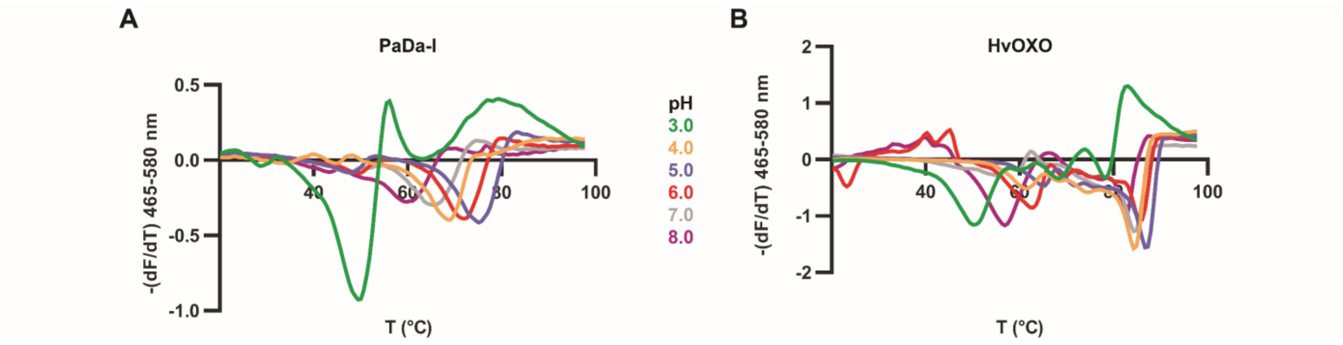

Figure S3. Melting temperature curves of PaDa-I (A) and HvOXO (B) at pH 3.0-8.0. Fluorescence was measured during enzyme denaturation by increasing the temperature from 20 to 98 °C. Light Cycler 480 SW 1.5.1 software was used to calculate the negative first derivatives (-dF/dt), which reveals melting temperatures as peaks. Curves at pH 3.0 were considered an assay artifact and thus the corresponding melting temperature values were not discussed in the manuscript. In the case of HvOXO, we ascribed the first observed increase in fluorescence to hexamer dissociation into inactive subunits.[6] The corresponding T_m values were discussed in the manuscript, while the second observed process was considered not relevant.

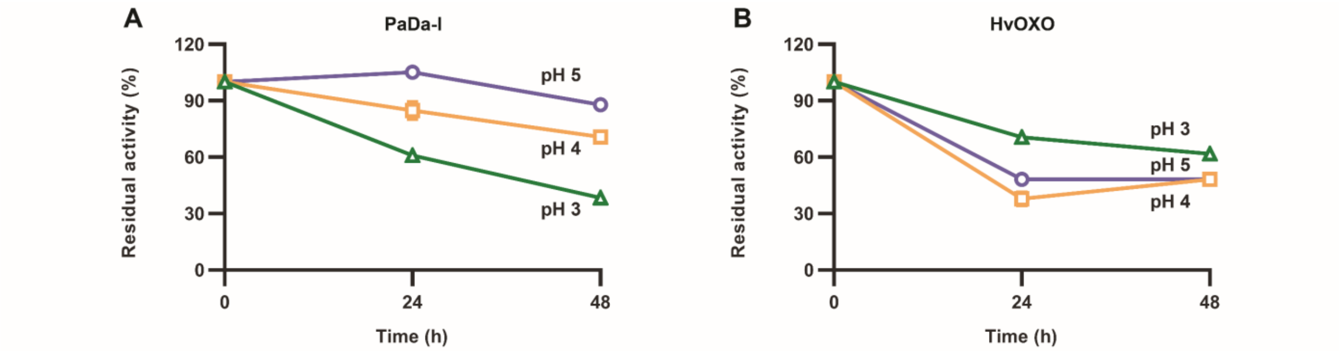

Figure S4. Long-term stability of PaDa-I (A) and HvOXO (B) at pH 3.0 (△), 4.0 (□) and 5.0 (○). Incubation and activity assays were performed at 25 ºC. The substrates 5-nitro-1,3-benzodioxole (PaDa-I) and oxalate (HvOXO) were used to determine the residual enzyme activity.

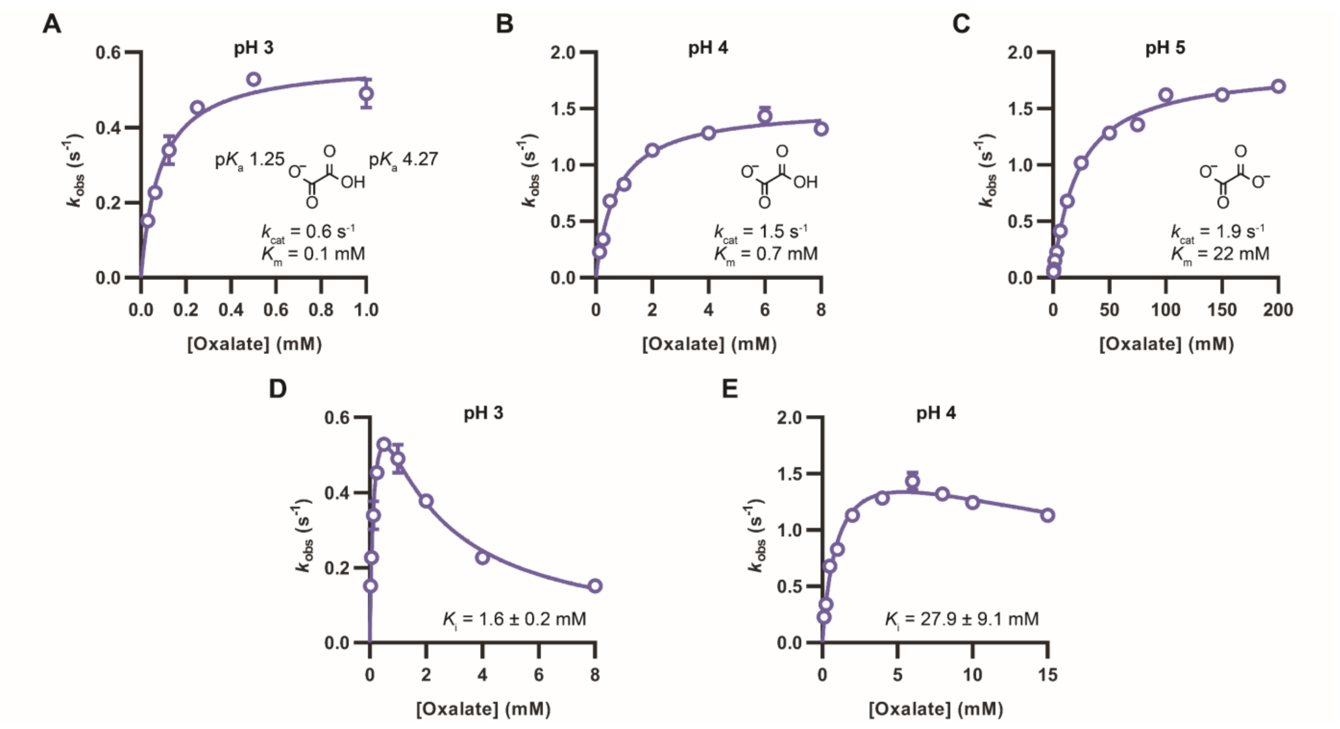

Figure S5. Oxalate oxidation rates in reactions catalysed by HvOXO at various pH values. Reactions contained 0.025 µM HvOXO, 0.125 µM PaDa-I, 0.03-200 mM potassium oxalate, 0.07 mM MBTH and 1 mM DMAB in air-saturated 100 mM citrate-phosphate at 25 ºC. Plots in panel **A-C** were fitted to the Michaelis-Menten equation to determine the steady-state kinetic parameters shown in **Table 1**. Plots in panel **D-E** were fitted to the substrate inhibition equation to determine the inhibition constant (K_i).

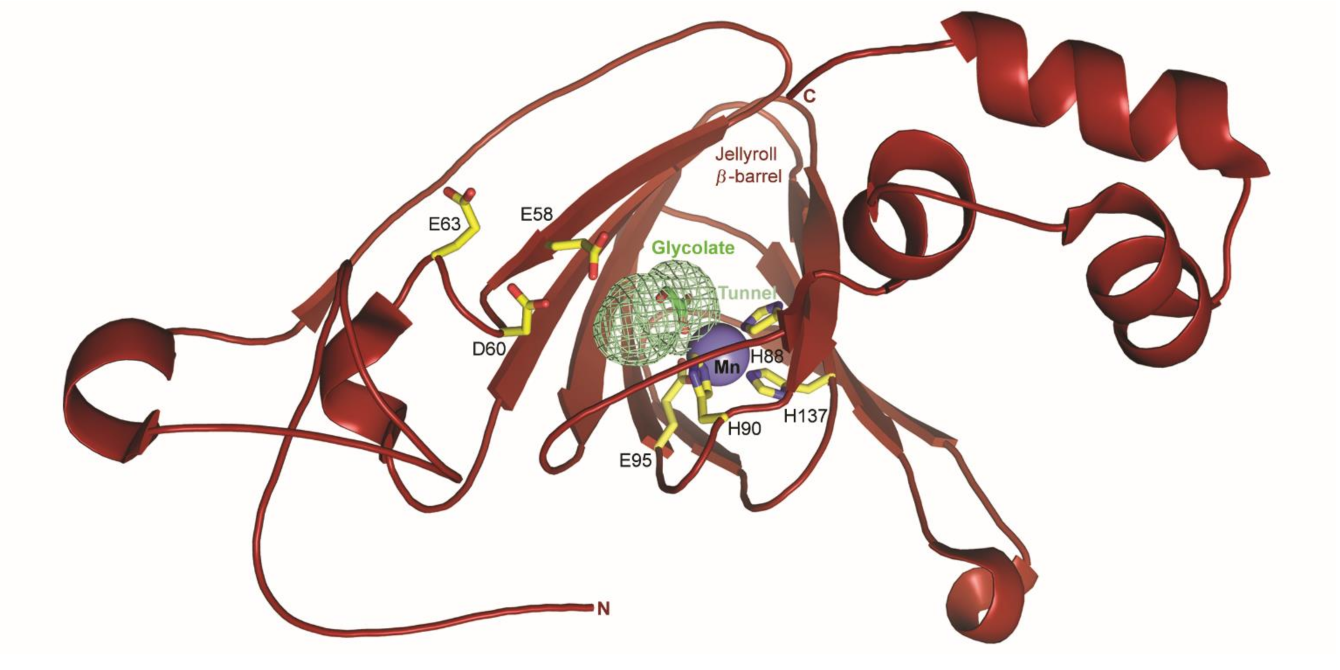

Figure S6. Crystal structure of recombinant HvOXO monomer in complex with the substrate analogue glycolate (PDB ID: 2ETE).[7] The tunnel, which leads from the Mn atom to the surrounding solvent, was calculated using CAVER 3.0.3 PyMol plugin. E58, D60 and E63 lie at the entrance of the tunnel. Interaction between these protonated residues and the oxalate monoanion may facilitate substrate access to the HvOXO active site.[8] The deprotonated carboxylate oxygen atom of oxalate likely binds to the Mn ion, resembling the HvOXO-glycolate complex shown in this figure.[7] Mn atom is bound to the side chains of conserved E95, H90, H137 and H88. When using the stick representation, carbon atoms corresponding to residues and glycolate are colored yellow and green, respectively. Mn atom is depicted as a sphere. Surface mesh of the tunnel volume is colored light green.

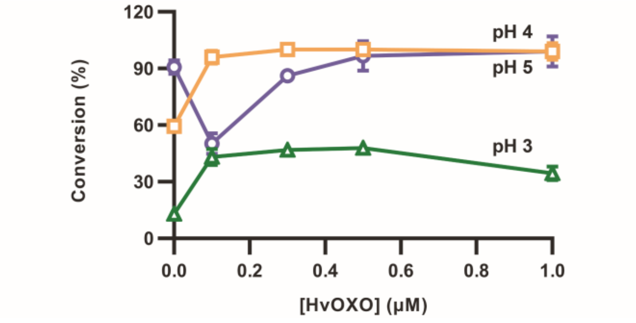

Figure S7. Influence of HvOXO:PaDa-I ratio on tolmetin (4) conversion at pH 3.0 (△), 4.0 (□) and 5.0 (○). 10 mM H_2O_2 was included only in the reactions without HvOXO, while all reactions with HvOXO contained 10 mM oxalate. All reactions (5 µL) contained 0.1 µM PaDa-I, 0.5 mM tolmetin and 1% dimethyl sulfoxide. Analyses were performed after 20.5 h of incubation.

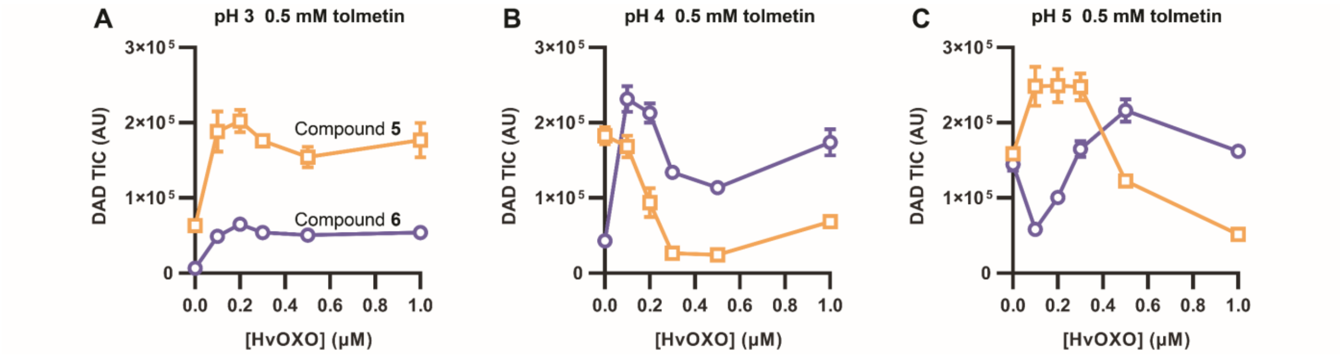

Figure S8. Peak areas corresponding to compound 5 (□) and 6 (○) which result from the reaction of PaDa-I with tolmetin (4) in the absence or the presence of an increasing HvOXO concentration at pH 3.0 (A), 4.0 (B) and 5.0 (C). Tolmetin conversion values calculated for these experiments are shown in **Fig. S7**. 10 mM H_2O_2 was included only in the reactions without HvOXO, while all reactions with HvOXO (0.1-1 µM) contained 10 mM oxalate. All reactions (5 µL) contained 0.1 µM PaDa-I, 0.5 mM tolmetin, buffer and 1% dimethyl sulfoxide. Analyses were performed after 20.5 h of incubation.

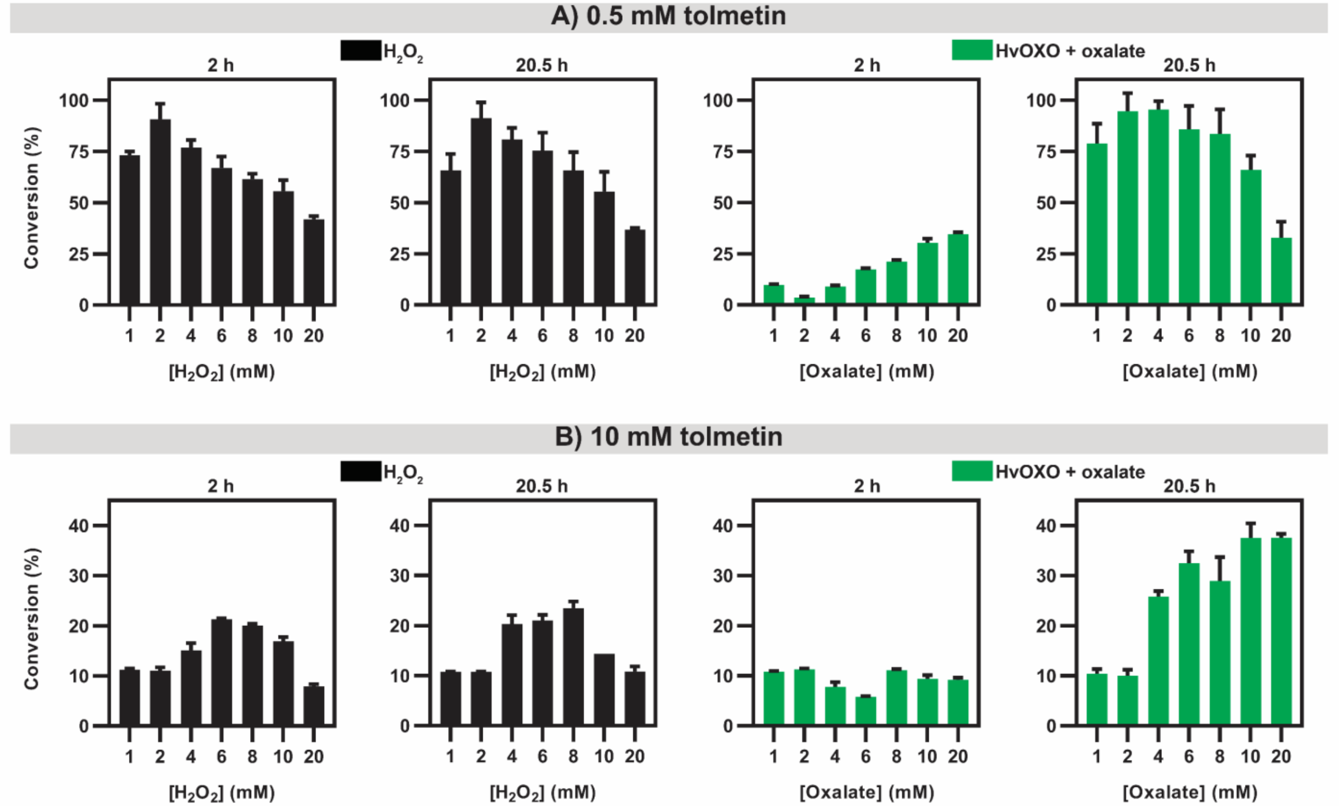

Figure S9. PaDa-I-catalysed conversion of 0.5 (A) and 10 (B) mM tolmetin with H_2O_2 or HvOXO. Reactions (5 µL) contained 0.1 µM PaDa-I, 0 or 0.1 µM HvOXO, 1-20 mM H_2O_2 or oxalate, buffer at pH 4.0. Reactions with 0.5 or 10 mM tolmetin contained 1 and 24% dimethyl sulfoxide, respectively.

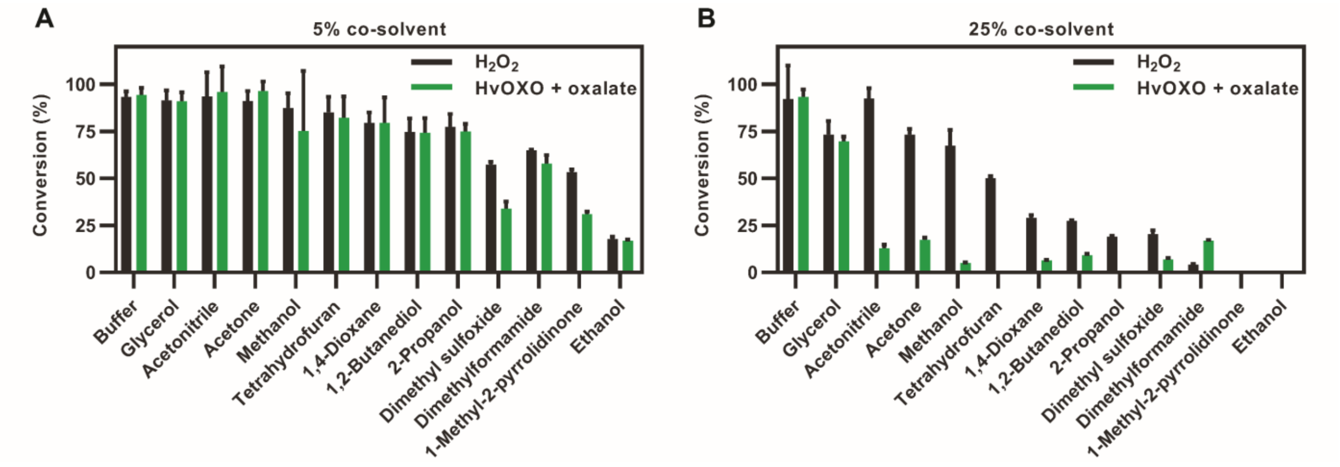

Figure S10. Influence of co-solvents on PaDa-I-catalysed tolmetin conversion with either H_2O_2 or HvOXO. Reactions (5 µL) contained 0.1 µM PaDa-I, 0 or 0.1 µM HvOXO, 0.5 mM tolmetin, 2 mM either oxalate or H_2O_2, buffer at pH 4.0. 42 mM tolmetin stock was prepared in dimethyl sulfoxide. Thus, all reactions contained 1% dimethyl sulfoxide in addition to 0-25% of the co-solvent under study. Analyses were performed after 20.5 h.

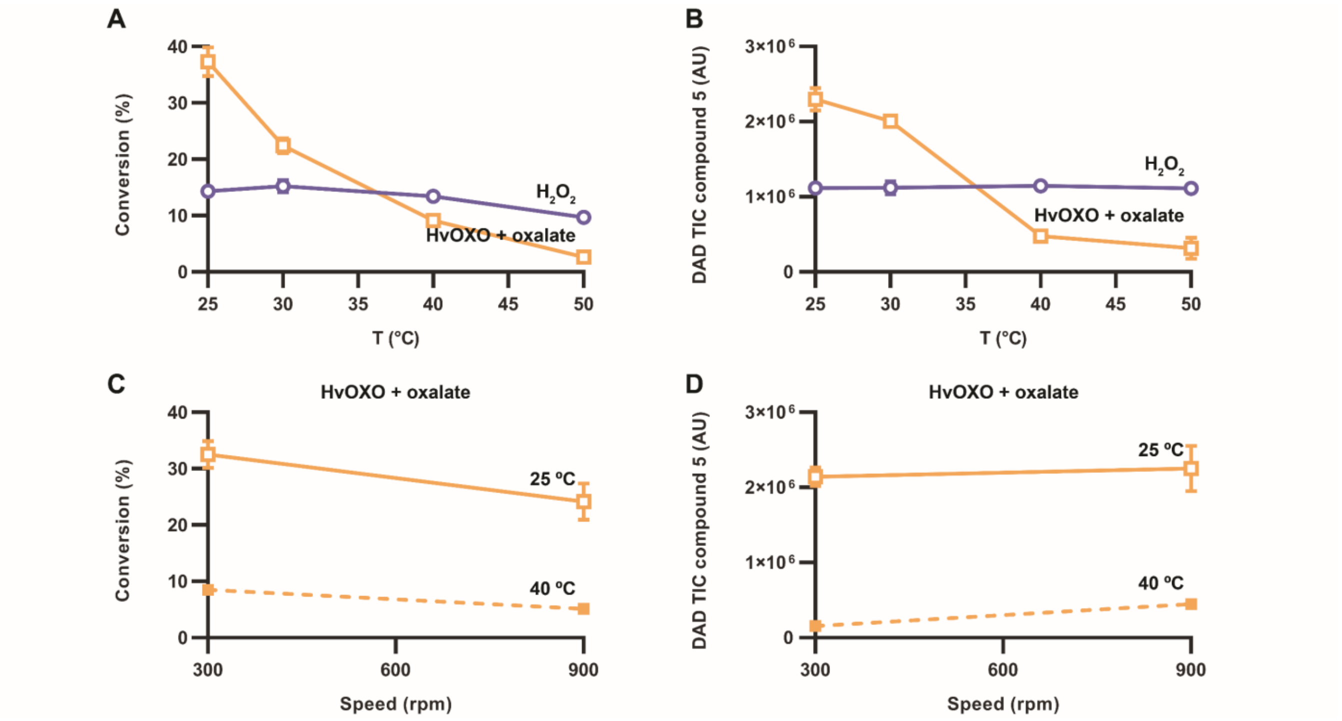

Figure S11. Influence of temperature (A-D) and mixing frequency (C-D) on the conversion of tolmetin catalysed by PaDa-I in the presence (A-D, □, ■) and the absence (A-B, O) of HvOXO. All reactions (6 µL, final volume) contained 0.1 µM PaDa-I, 8 mM tolmetin, buffer at pH 4.0 and 19% dimethyl sulfoxide. Reactions with and without 0.1 µM HvOXO contained 8 mM oxalate and H_2O_2, respectively. Analyses were performed after 20.5 h of incubation at 300 rpm (**A**, **B**) or at either 300 or 900 rpm (**C**, **D**). Peak areas (DAD TIC, UPLC-MS) corresponding to reaction product **5** (0.32 min, $[M+H]^+$ 274 m/z) are shown in **panel-B** and **D**. Peak areas (DAD TIC, UPLC-MS) for reaction product **6** (0.53 min, $[M+H]^+$ 272 m/z) were only 4-14% respect to product **5**, independent on both temperature and mixing frequency and are not shown for the sake of clarity. 10-14% conversions were observed for PaDa-I-catalysed reactions containing 8 mM H_2O_2 at 25-50 °C. In the presence of HvOXO and 8 mM oxalate (instead of H_2O_2), a 2.6- and 1.5-fold higher conversion yield was observed at 25 and 30 °C, respectively. A significantly reduced conversion was observed using the H_2O_2-generation system at 40 and 50 °C (9 and 3%). Thus, a temperature of 25 °C is optimal for the PaDa-I reactions containing HvOXO. Similar results were obtained for these experiments at 300 and 900 rpm (despite of having a higher dioxygen transfer rate at 900 rpm), which suggests that the lower solubility of dioxygen at the highest assayed temperatures was not the main reason for the decreased conversions.

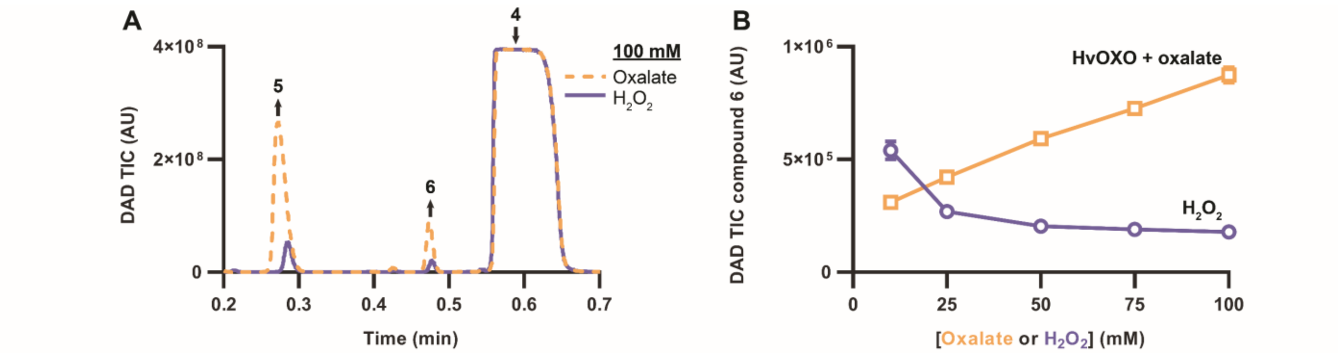

Figure S12. PaDa-I-catalysed conversion of 100 mM tolmetin with H_2O_2 or HvOXO. Reactions (5 µL) contained 0.1 µM PaDa-I, 0 or 0.1 µM HvOXO, buffer at pH 4.0 and 11% acetonitrile. Reactions with HvOXO contained 10-100 mM oxalate while reactions without HvOXO contained 10-100 mM H_2O_2. **Panel-A** shows reactions with100 mM either oxalate or H_2O_2. Peak areas for product **6** are plot in **panel-B**. Those for product **5** are shown in **Fig. 6**. UPLC-MS analyses were performed after 20.5 h.

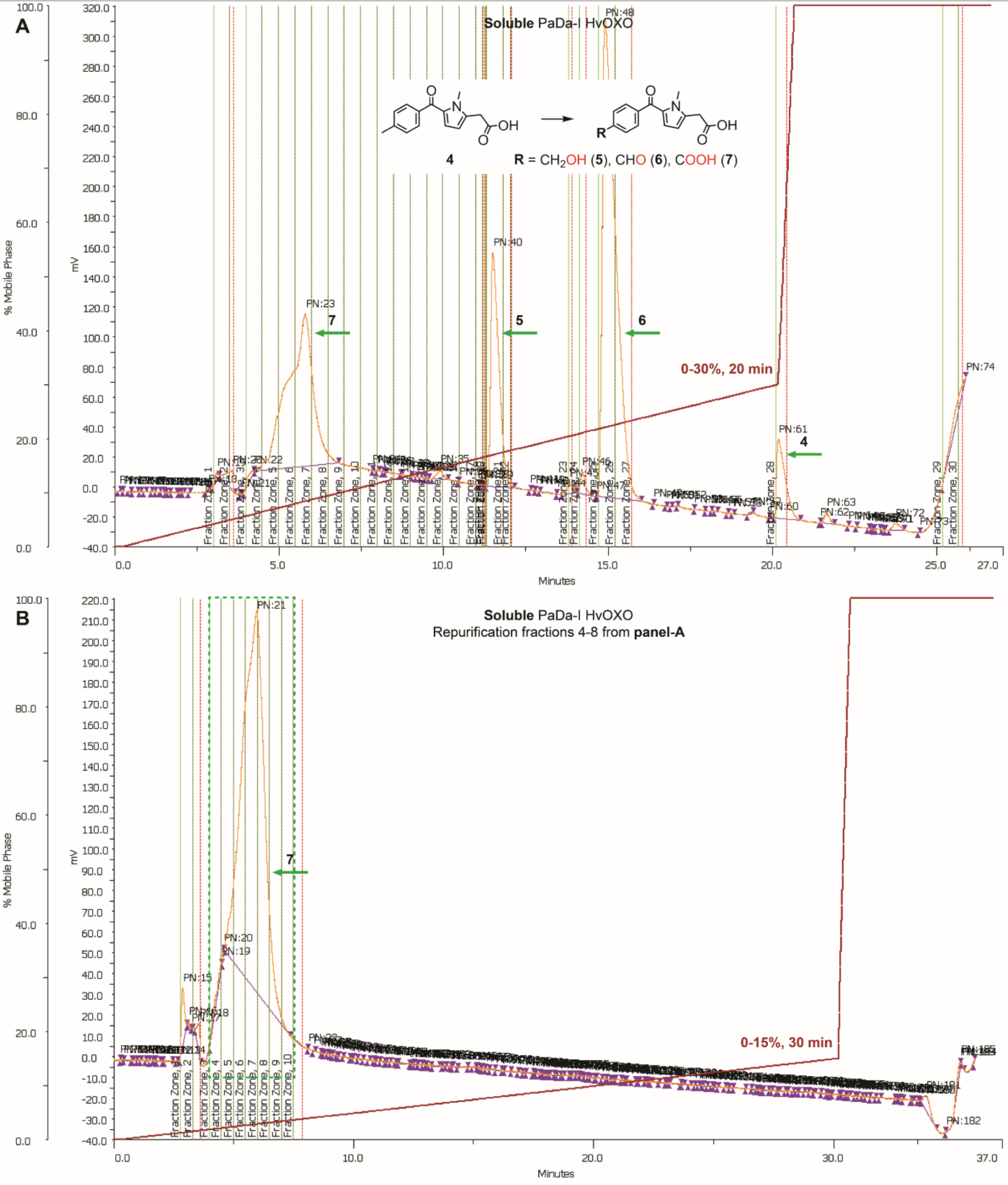

Figure S13. HPLC chromatograms of the reaction containing 50 mg tolmetin and soluble PaDa-I and HvOXO. Experimental details are indicated in **Section S1.13**. Corresponding UPLC-QTOF analyses are shown in **Fig. S14-15**.

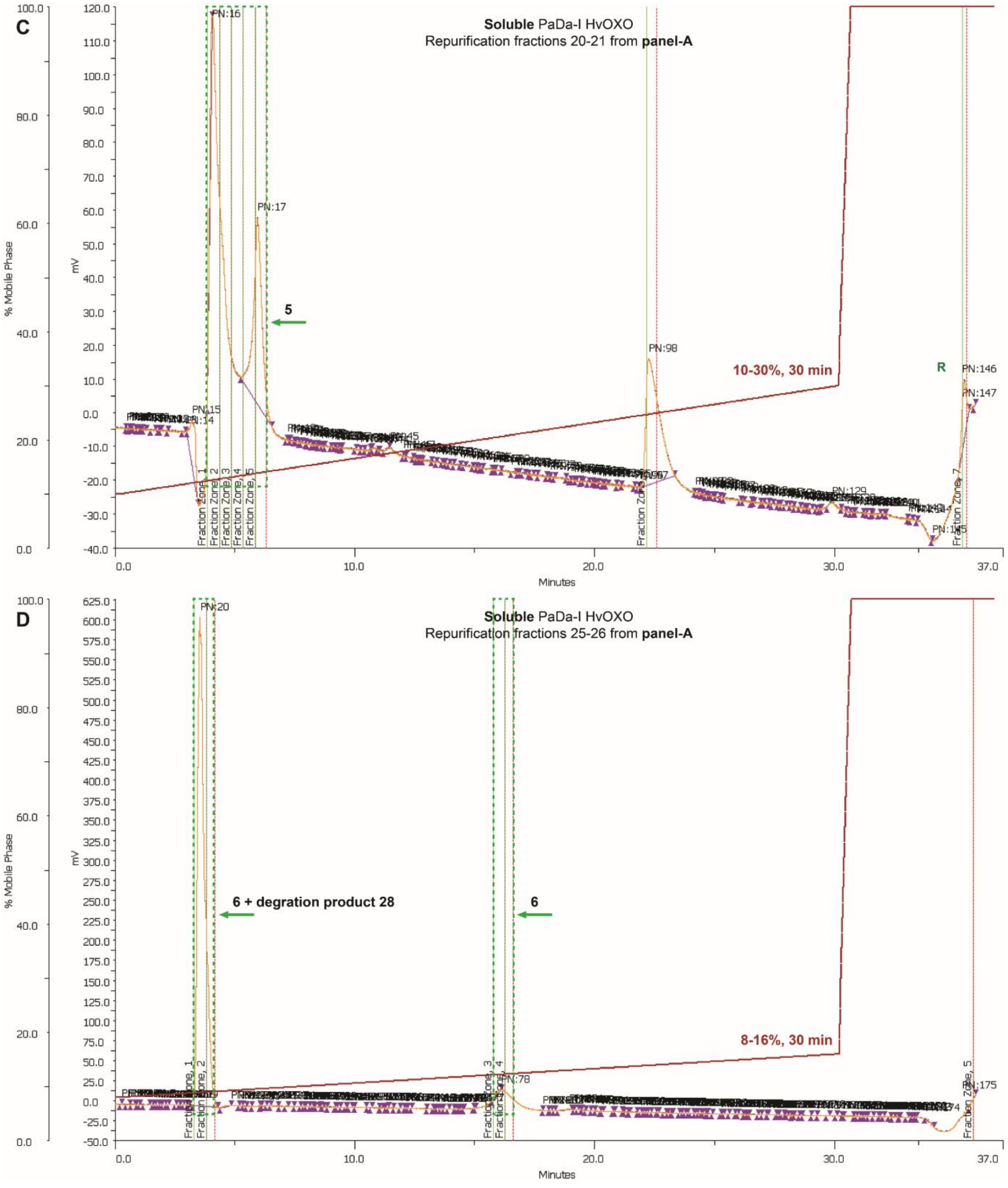

Figure S13. Continued.

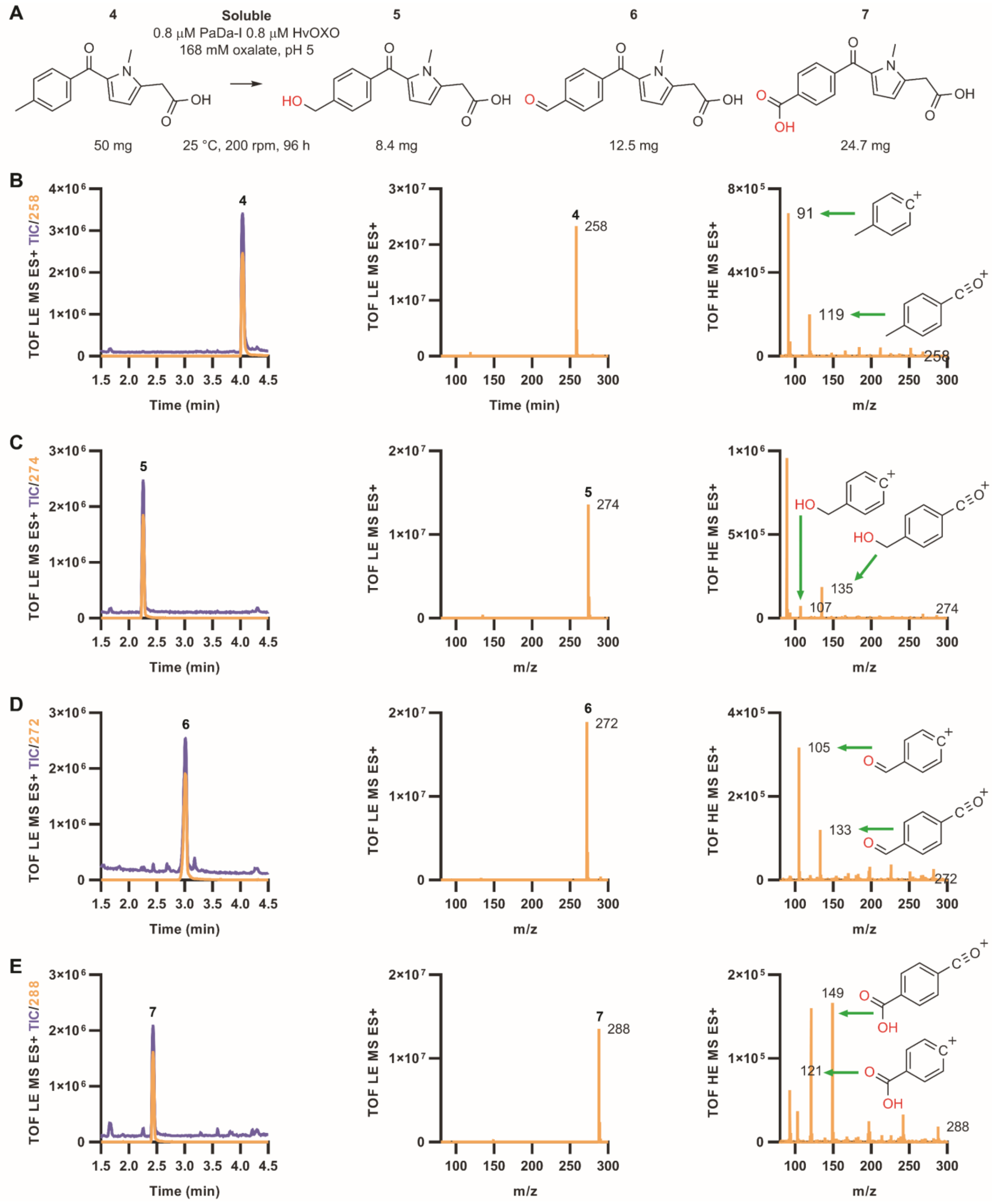

Figure S14. UPLC-QTOF analyses of the products purified from the reaction containing 50 mg tolmetin and soluble PaDa-I and HvOXO. Reaction conditions and structures are shown in **panel A**. **Panel B-D** show the low and high energy (LE and HE, respectively, as indicated in each y-axis label) UPLC-QTOF data for tolmetin (**B**) and three purified reaction products (**5**, **6** and **7** in **C-E,** respectively). The total ion current (TIC, purple) and the ion current resulting from a specified mass (orange) are shown as a function of time. Additional experimental details are described in **Section S1.13-S1.14** and **Fig. S13**. **Panel D** shows fractions 3-4 which were obtained during the HPLC analyses shown in **Fig. S13D** (see **Fig. S15** for fractions 1-2).

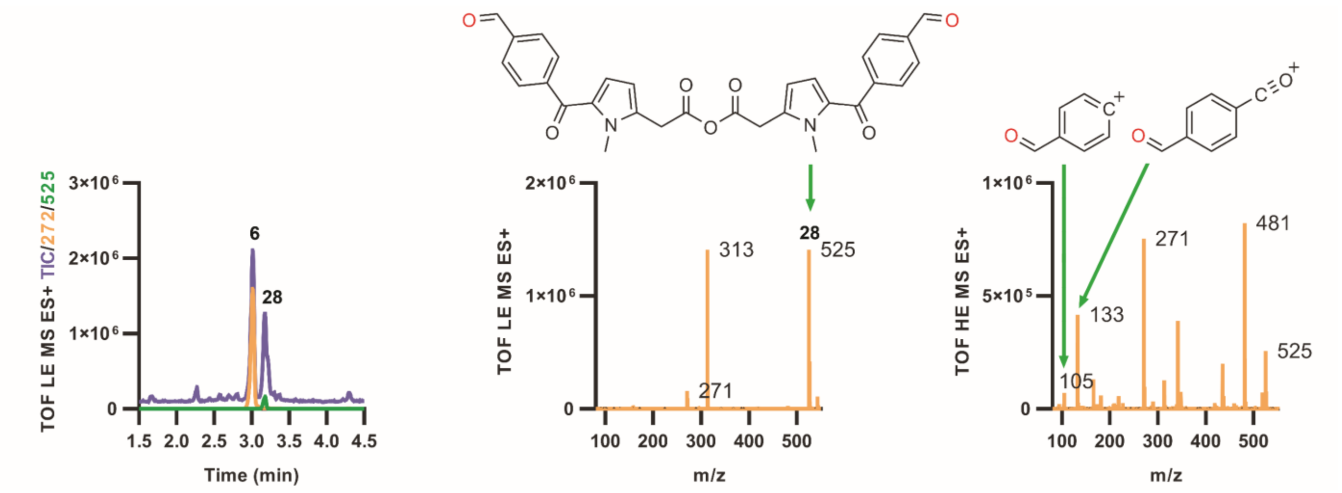

Figure S15. UPLC-QTOF analyses of the fractions 1-2 which were obtained during the HPLC analyses shown in Fig. S13D. During the purification and concentration processes, product **6** partially converted into compound **28**. A tentative structure of compound **28** and corresponding mass analyses are shown. UPLC-QTOF data for purified product **6** are shown in **Fig. S14D**.

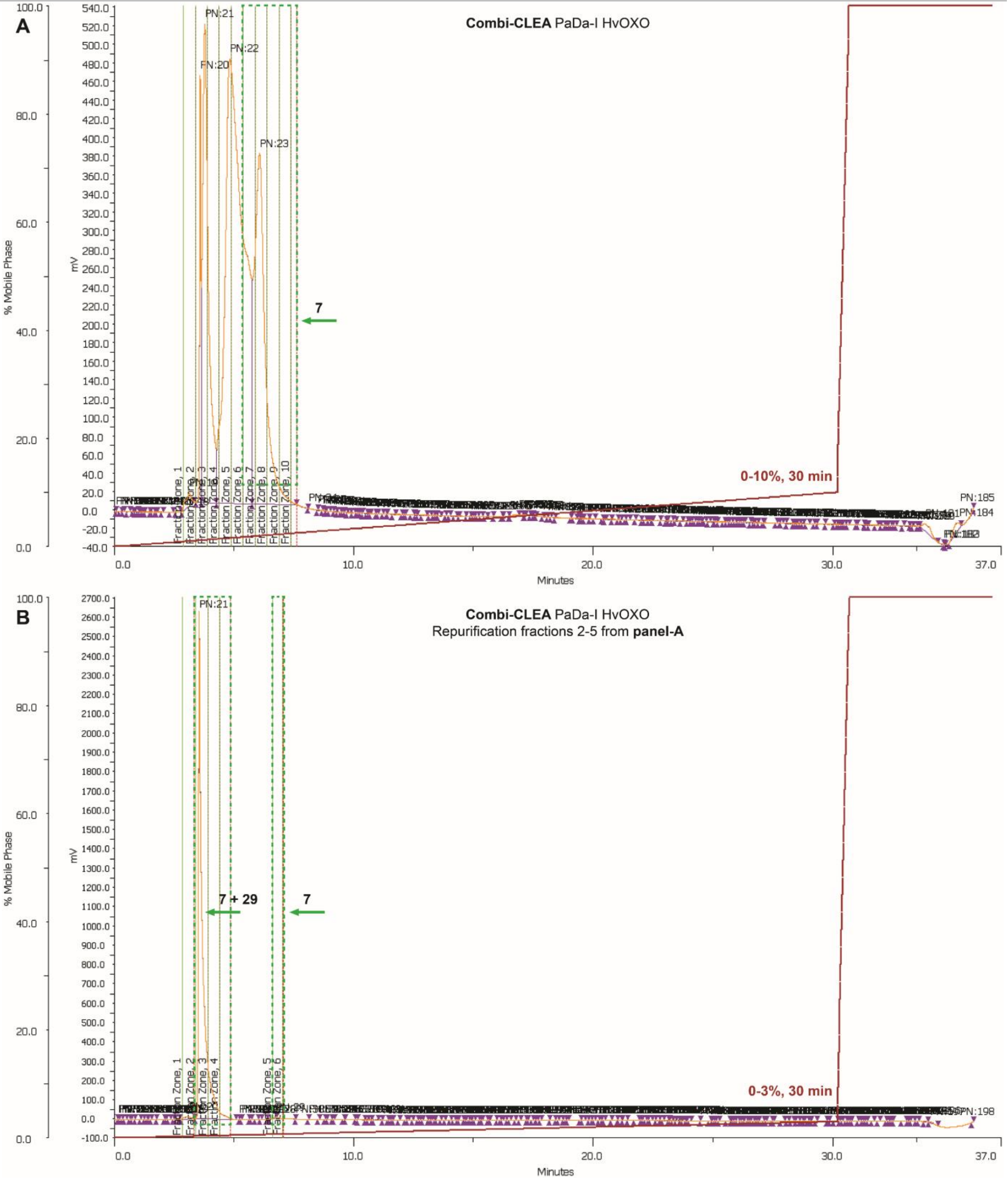

Figure S16. HPLC chromatograms of the reaction containing 50 mg tolmetin and combi-CLEA PaDa-I and HvOXO. Experimental details are indicated in **Section S1.13**. Corresponding UPLC-QTOF analyses are shown in **Fig. S17**.

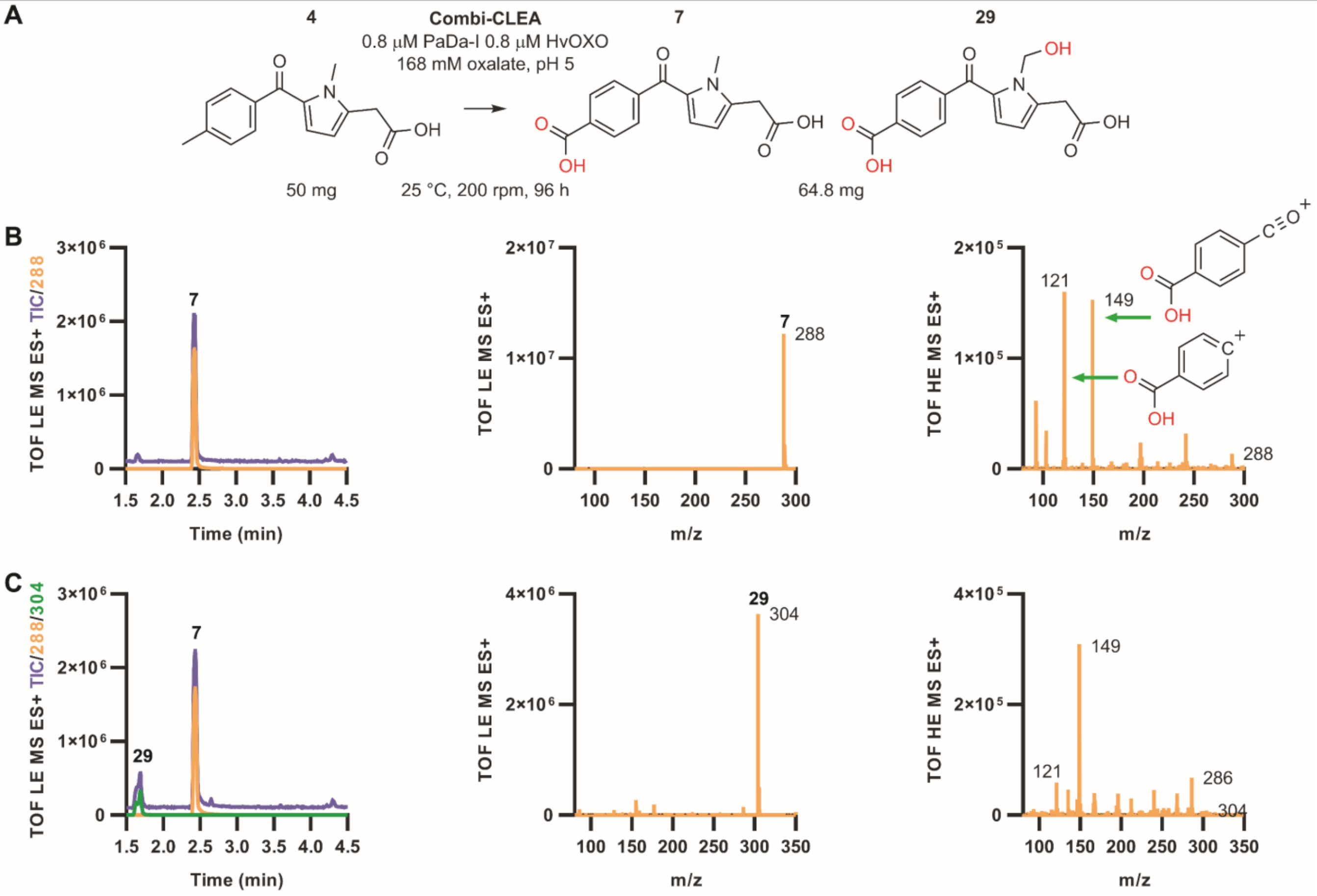

Figure S17. UPLC-QTOF analyses of the products purified from the reaction containing 50 mg tolmetin and combi-CLEA PaDa-I and HvOXO. Reaction conditions and structures are shown in **panel A**. **Panel B** shows the LE and HE data as a function of m/z for product **7** (fractions 5-6 in **Fig. S16**). **Panel C** shows the same for the minor product **29** (fractions 2-4 in **Fig. S16**, $[M+H]^+$ 304 m/z). The total ion current (TIC, purple) and the ion current resulting from a specified mass (orange and green) are shown as a function of time for a mixture of fractions 5-6 (**B**) or fractions 2-4 (**C**). A tentative structure of compound **29** is shown in **panel A**, although the position of the hydroxyl group on the pyrrole ring was not univocally assigned. Additional experimental details are described in **Section S1.13-S1.14** and **Fig. S16**.

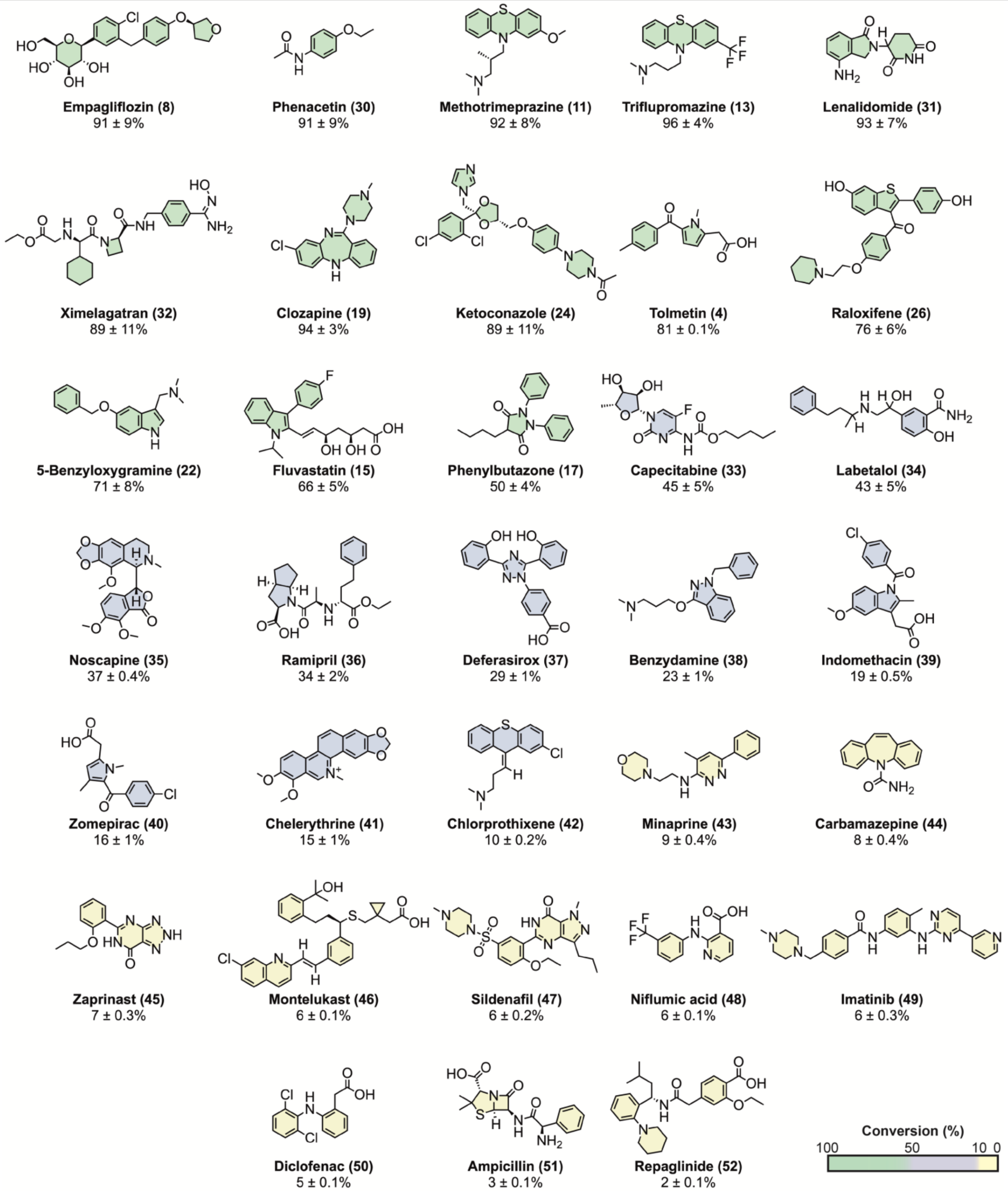

Figure S18. Drugs converted by PaDa-I. All reactions (5 µL) contained 0.8 µM PaDa-I, 0.8 µM HvOXO, 0.5 mM drug, 10 mM oxalate, buffer at pH 4.0, 2.5% either acetonitrile or tetrahydrofuran (**Table S2**). Conversions (%) after 24 h are indicated**.**

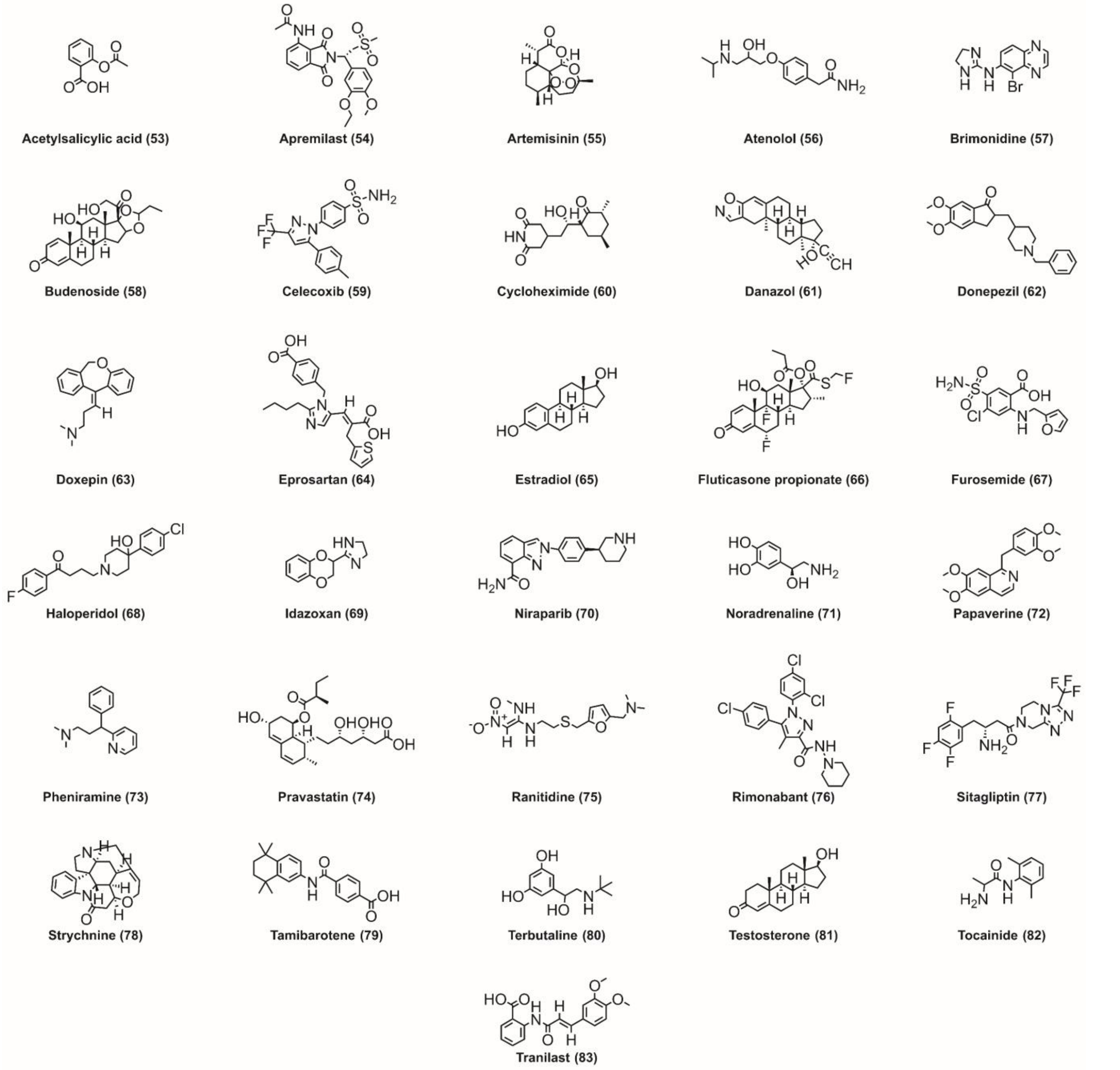

Figure S19. Drugs not converted by PaDa-I. All reactions (5 µL) contained 0.8 µM PaDa-I, 0.8 µM HvOXO, 0.5 mM drug, 10 mM oxalate, buffer at pH 4.0, 2.5% either acetonitrile or tetrahydrofuran (**Table S2**). Incubation time was 24 h.

S2.2. NMR spectral data

S2.2.1. Tolmetin (**4**), 2-(1-methyl-5-(4-methylbenzoyl)-1*H*-pyrrol-2-yl)acetic acid

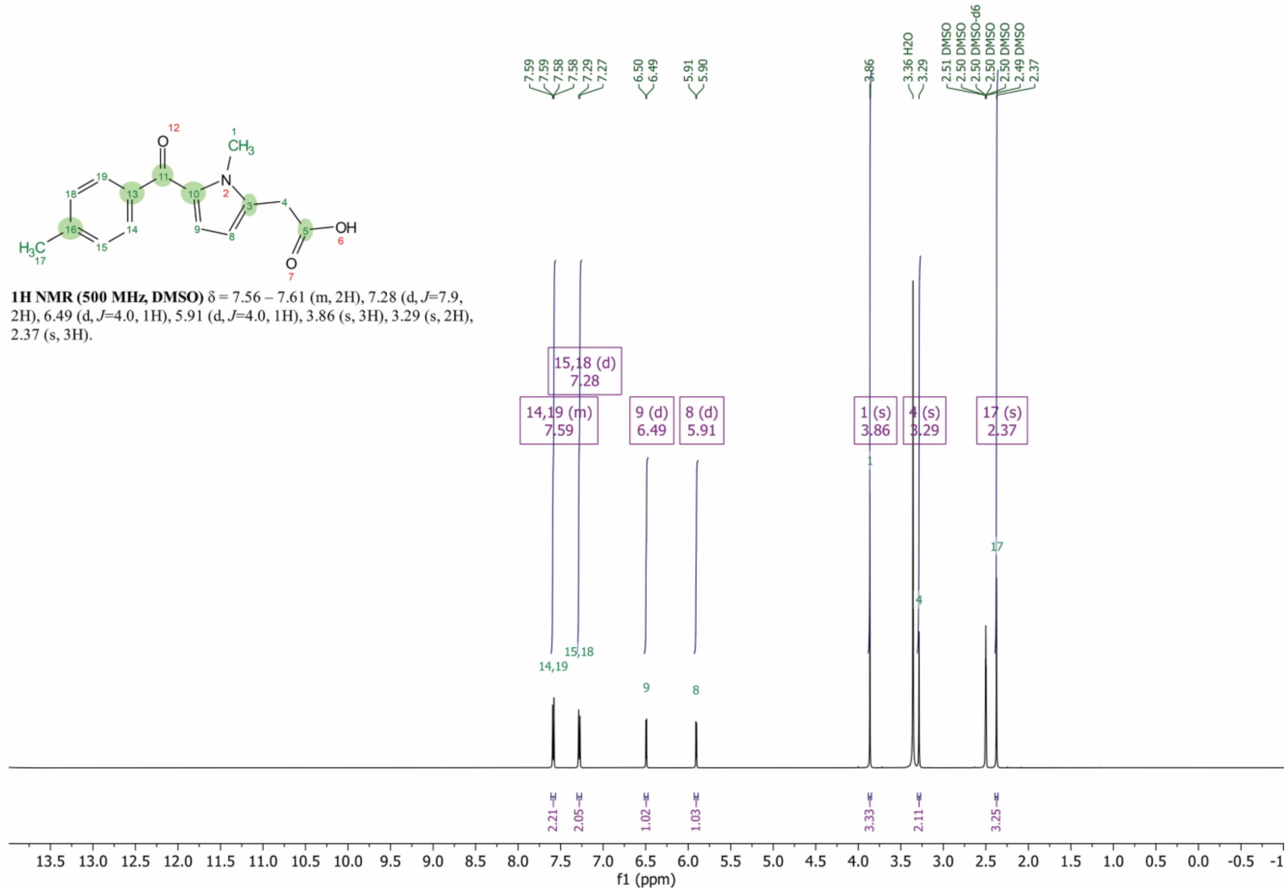

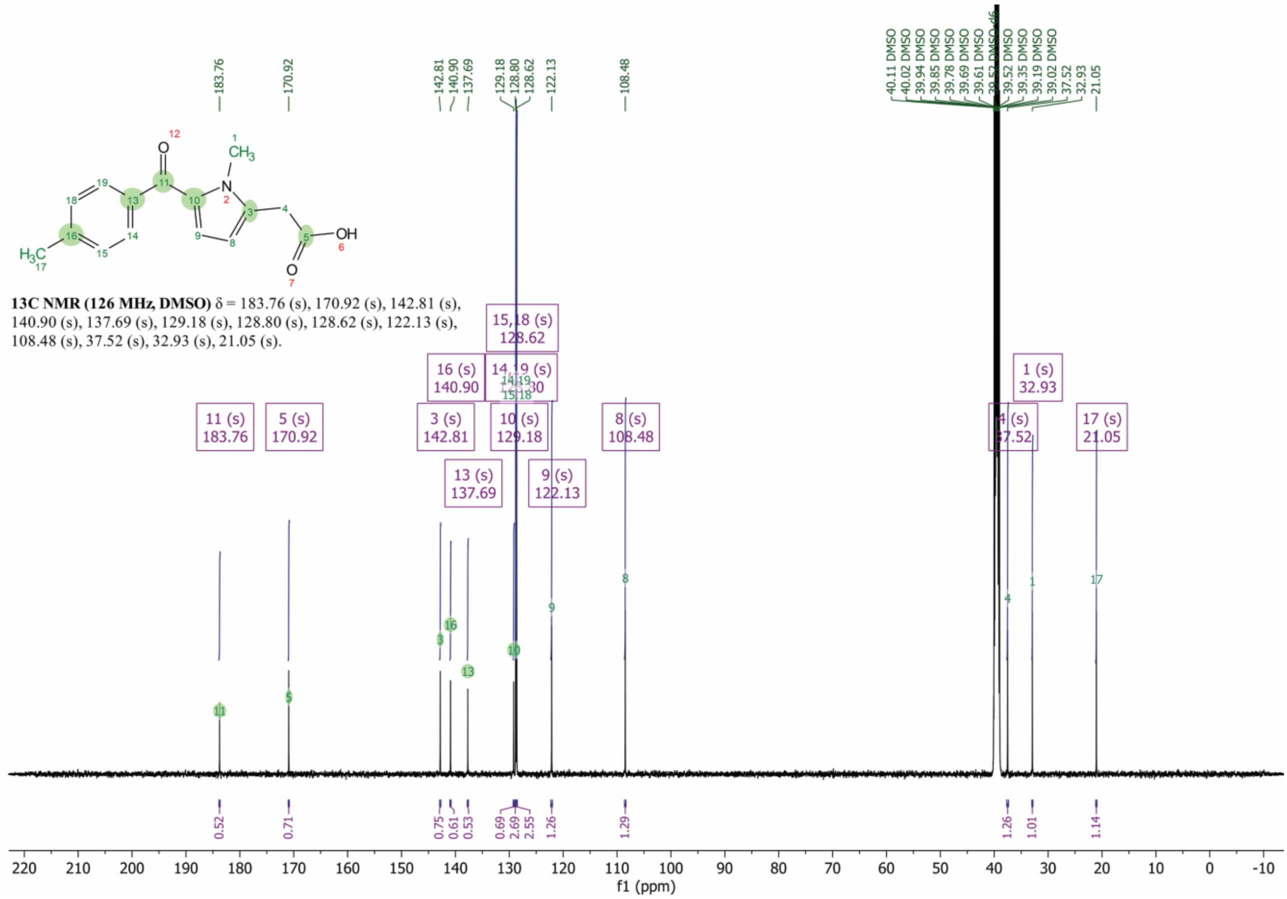

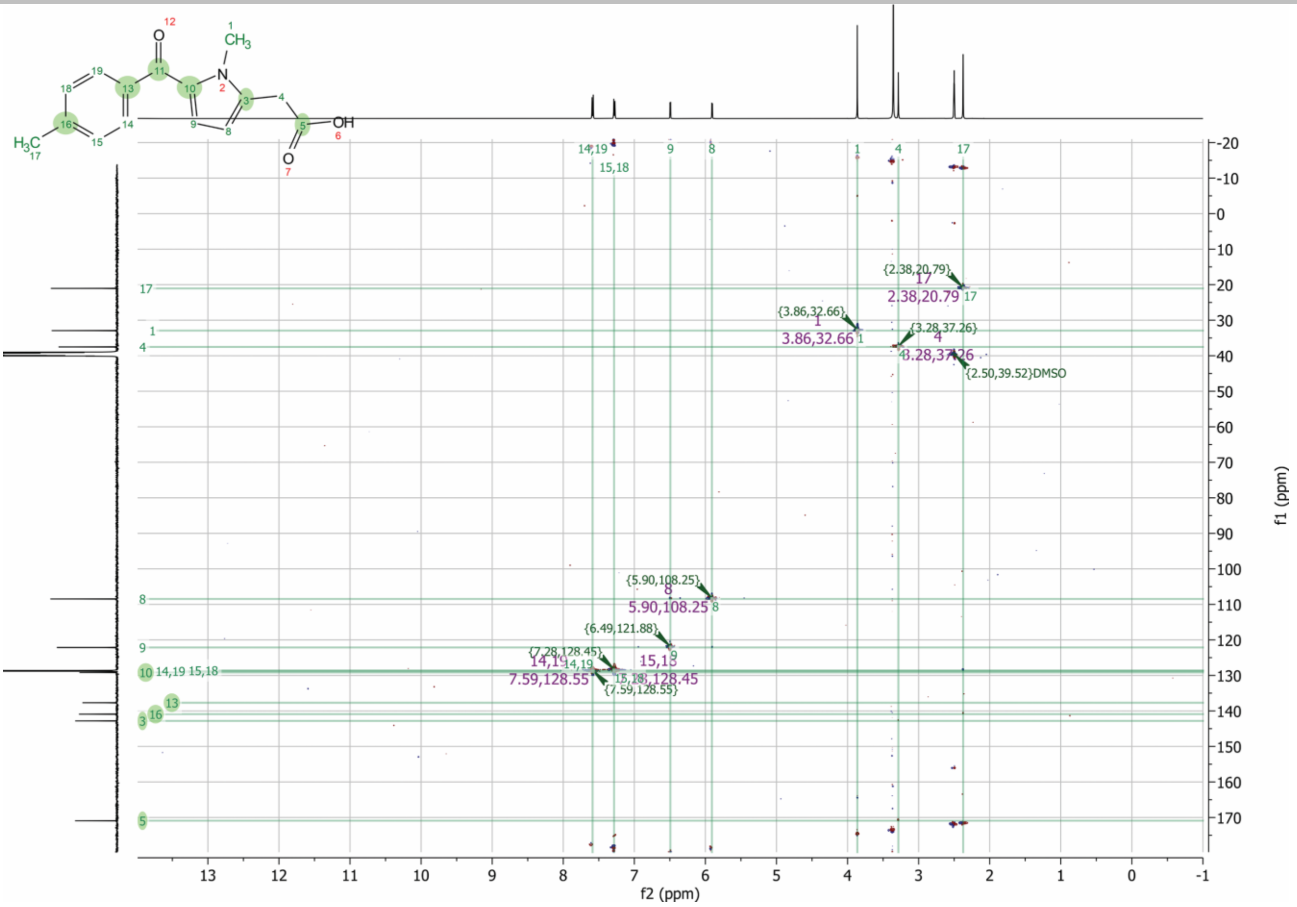

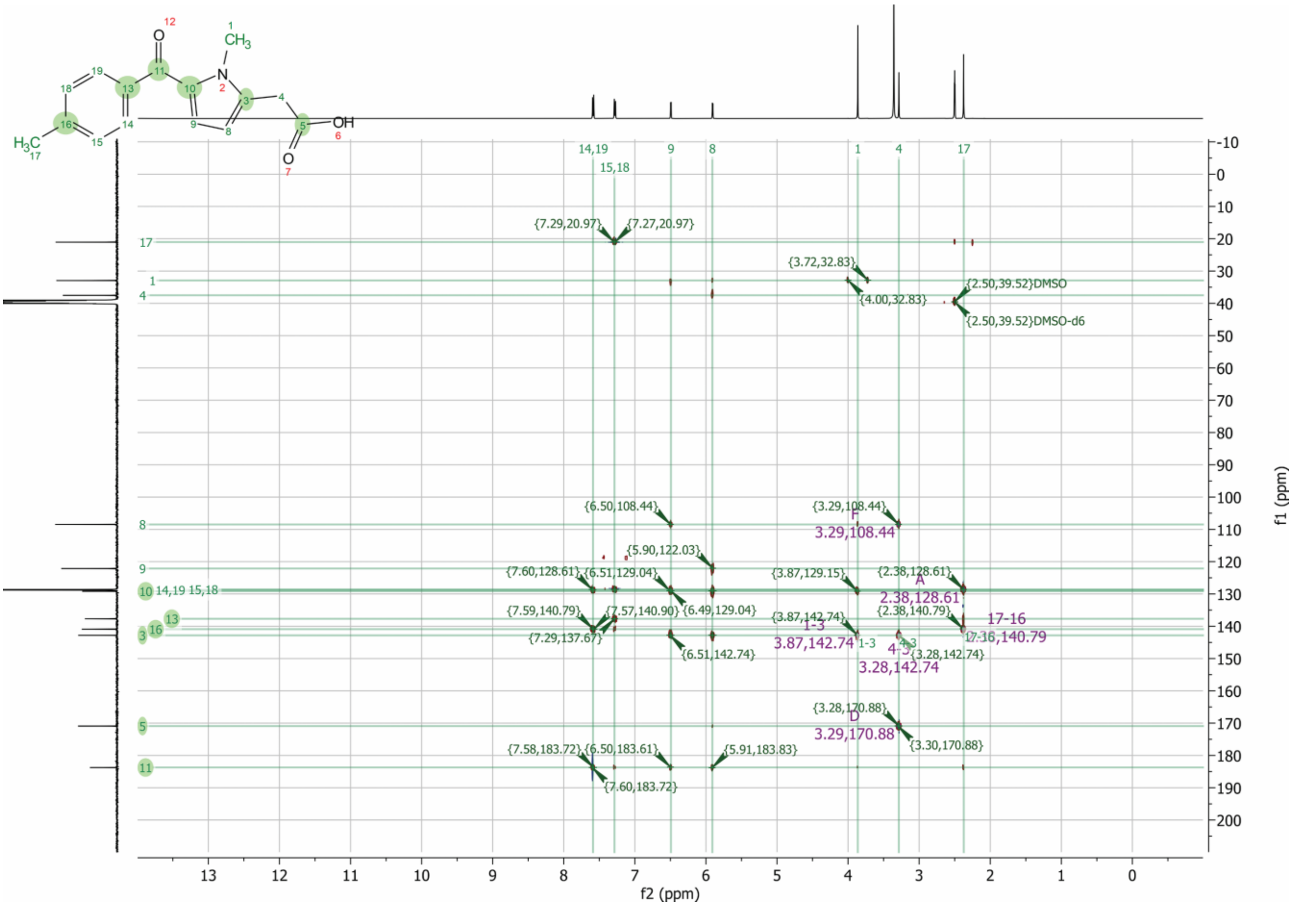

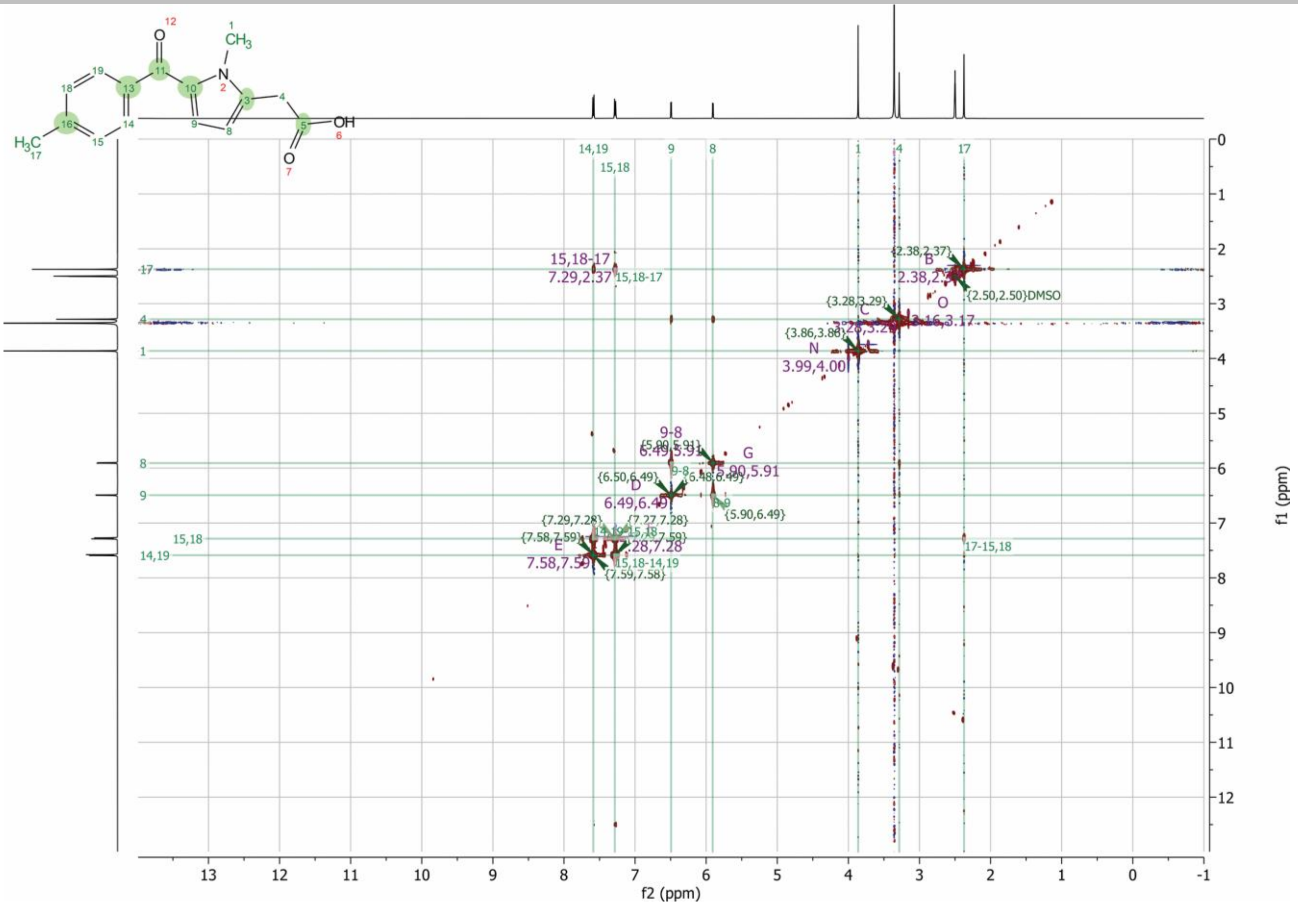

S2.2.2. 2-(5-(4-(hydroxymethyl)benzoyl)-1-methyl-1*H*-pyrrol-2-yl)acetic acid (**5**)

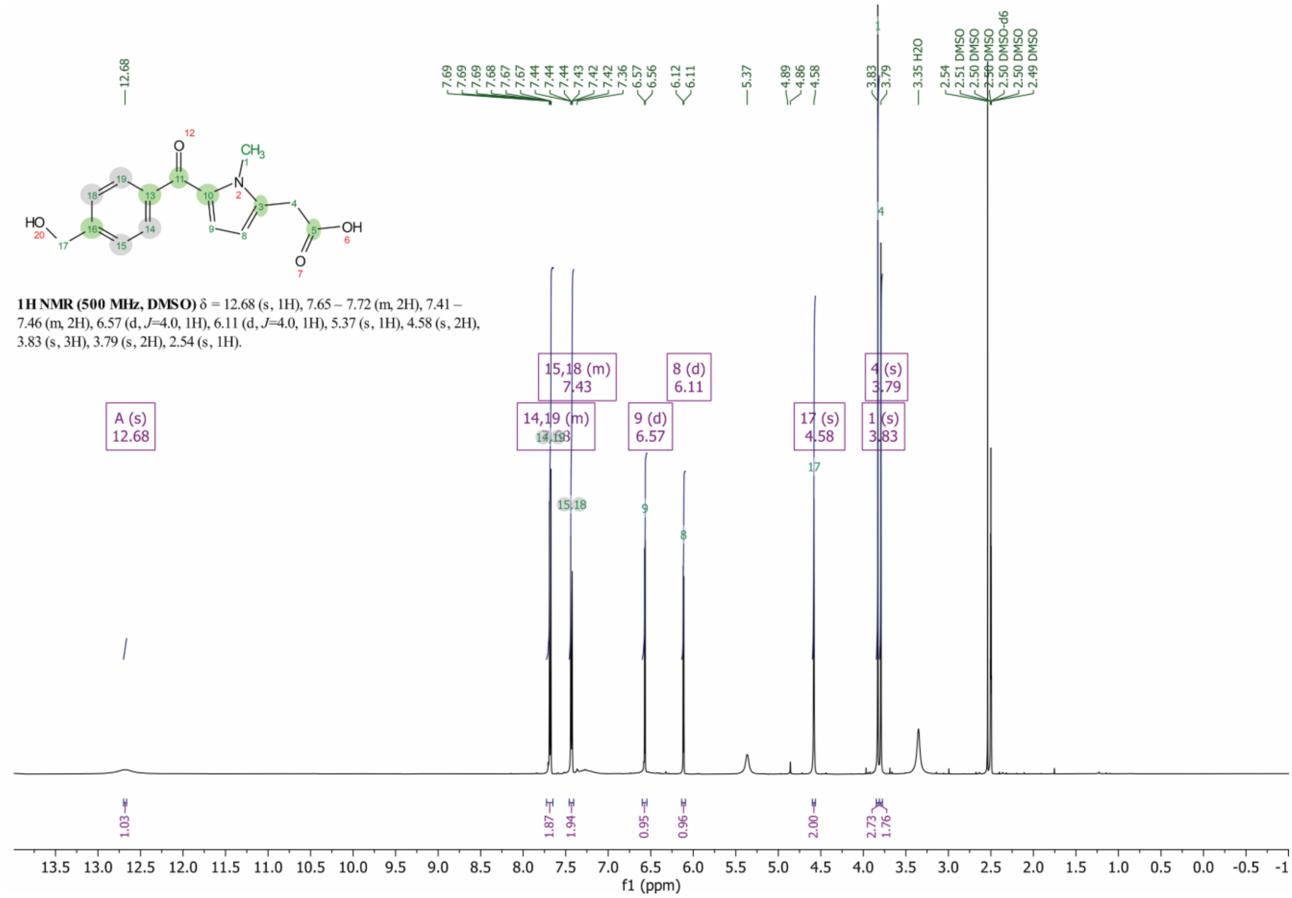

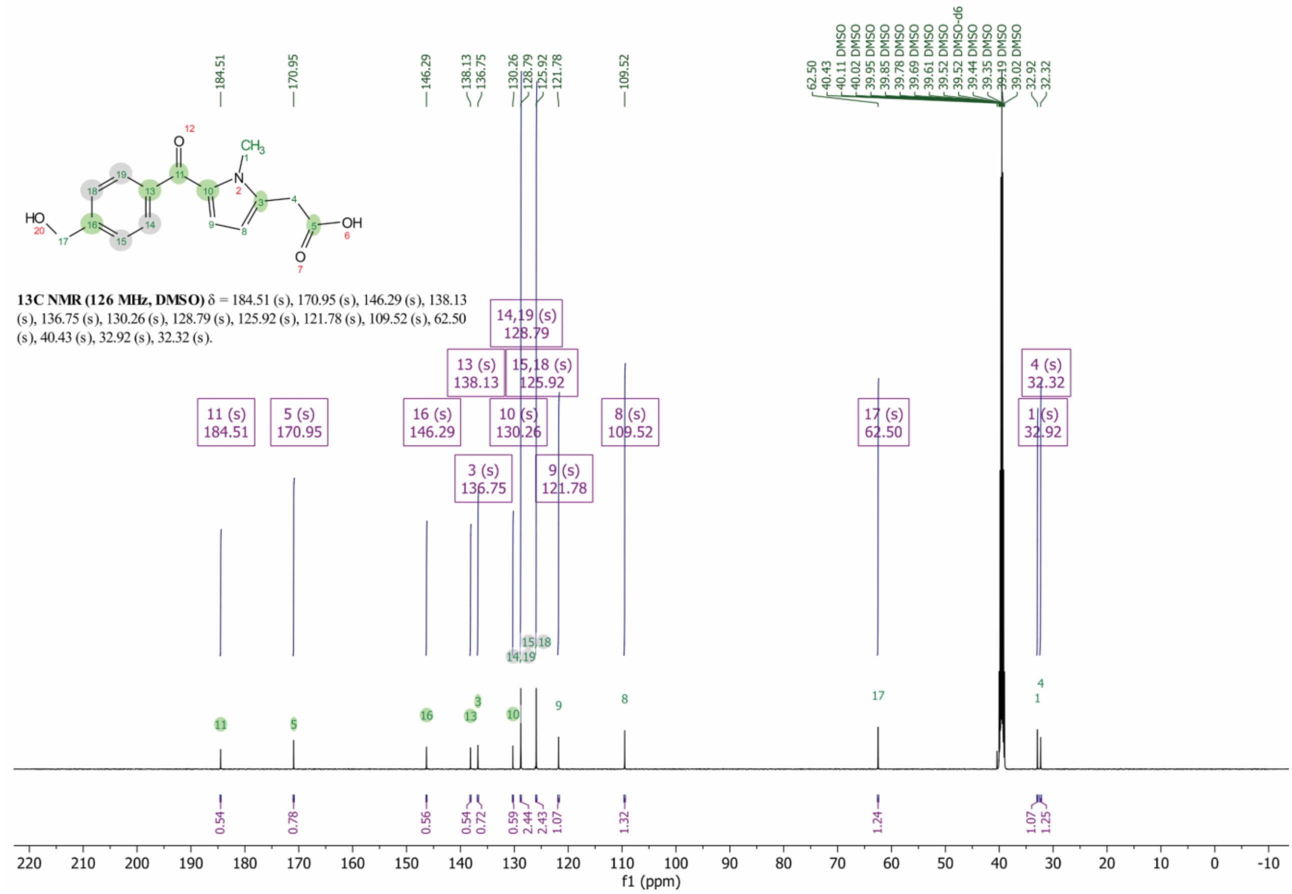

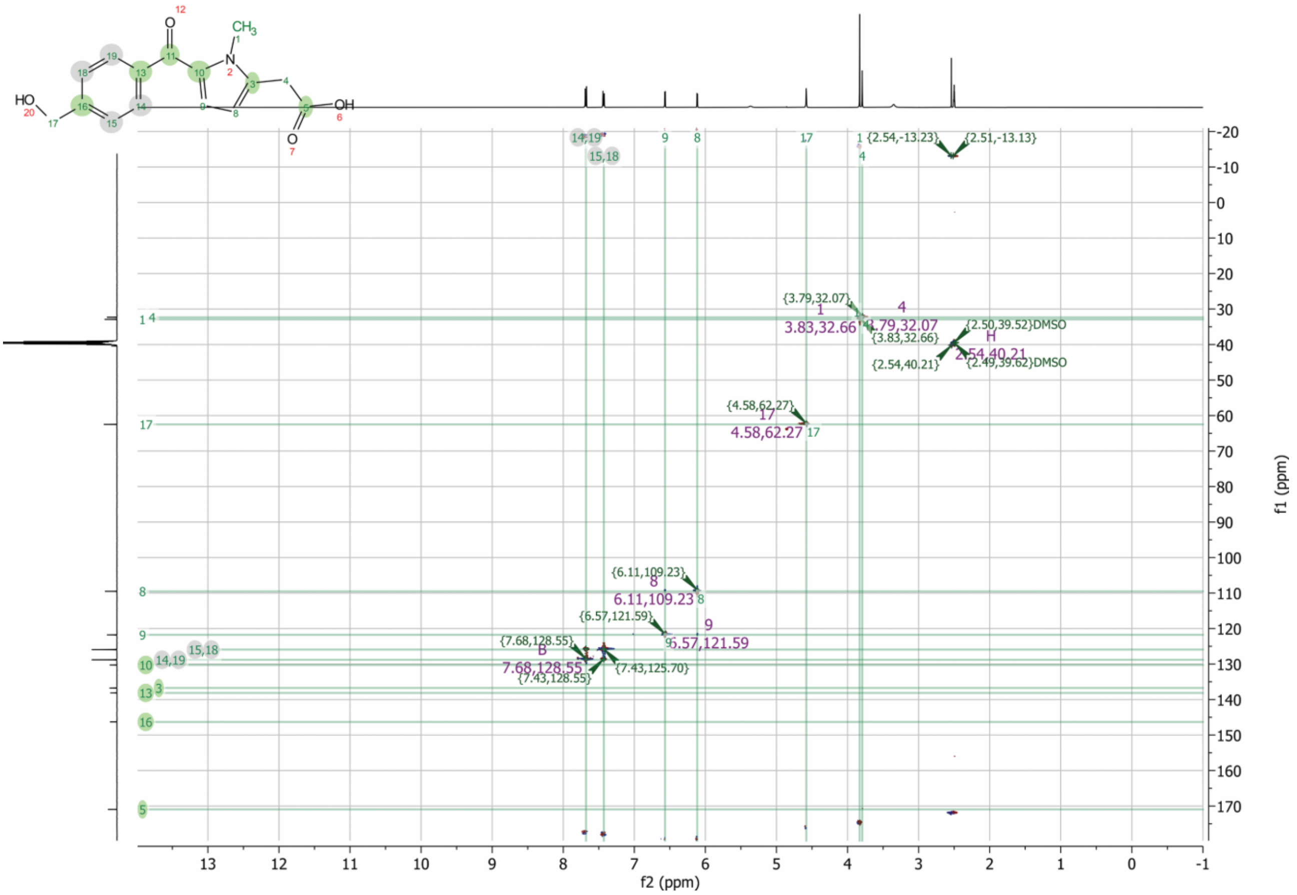
f2 (ppm)
f1 (ppm)

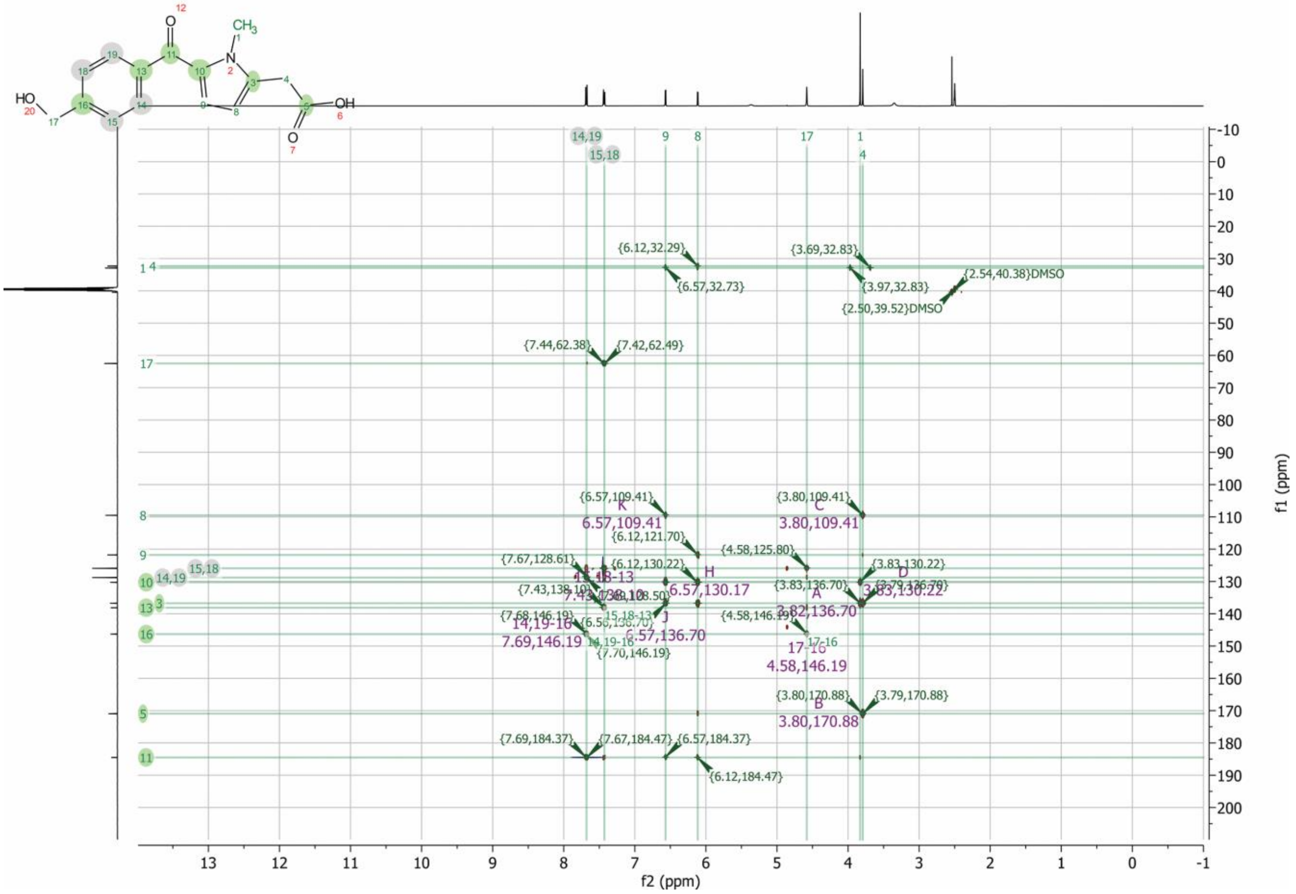
f2 (ppm)
f1 (ppm)

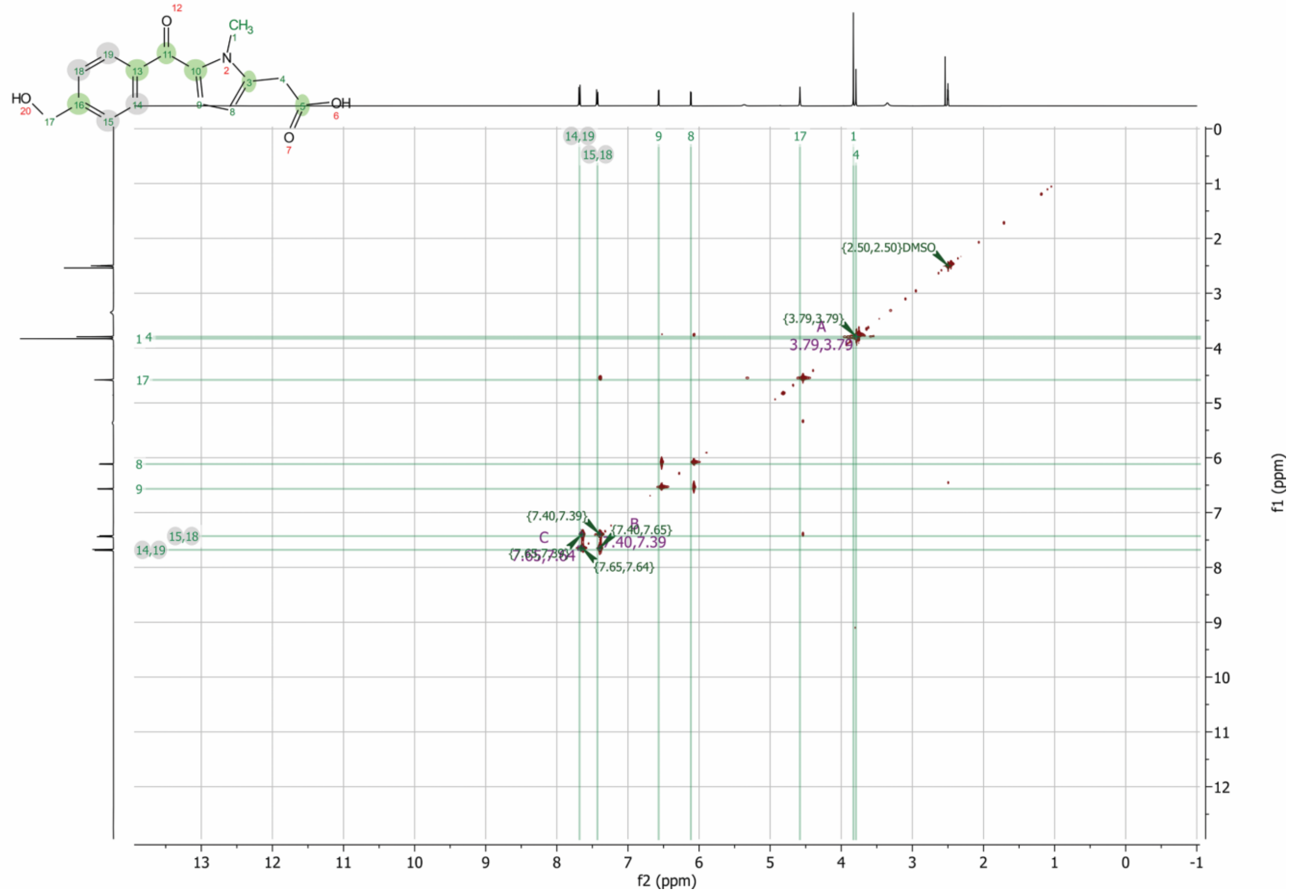
{2.50,2.50}DMSO
{3.79,3.79}
A
3.79,3.79
{7.40,7.39}
B
{7.40,7.65}
7.40,7.39
C
{7.65,7.39}
7.65,7.64
{7.65,7.64}
f2 (ppm)
f1 (ppm)

S2.2.3. 2-(5-(4-formylbenzoyl)-1-methyl-1*H*-pyrrol-2-yl)acetic acid (**6**)

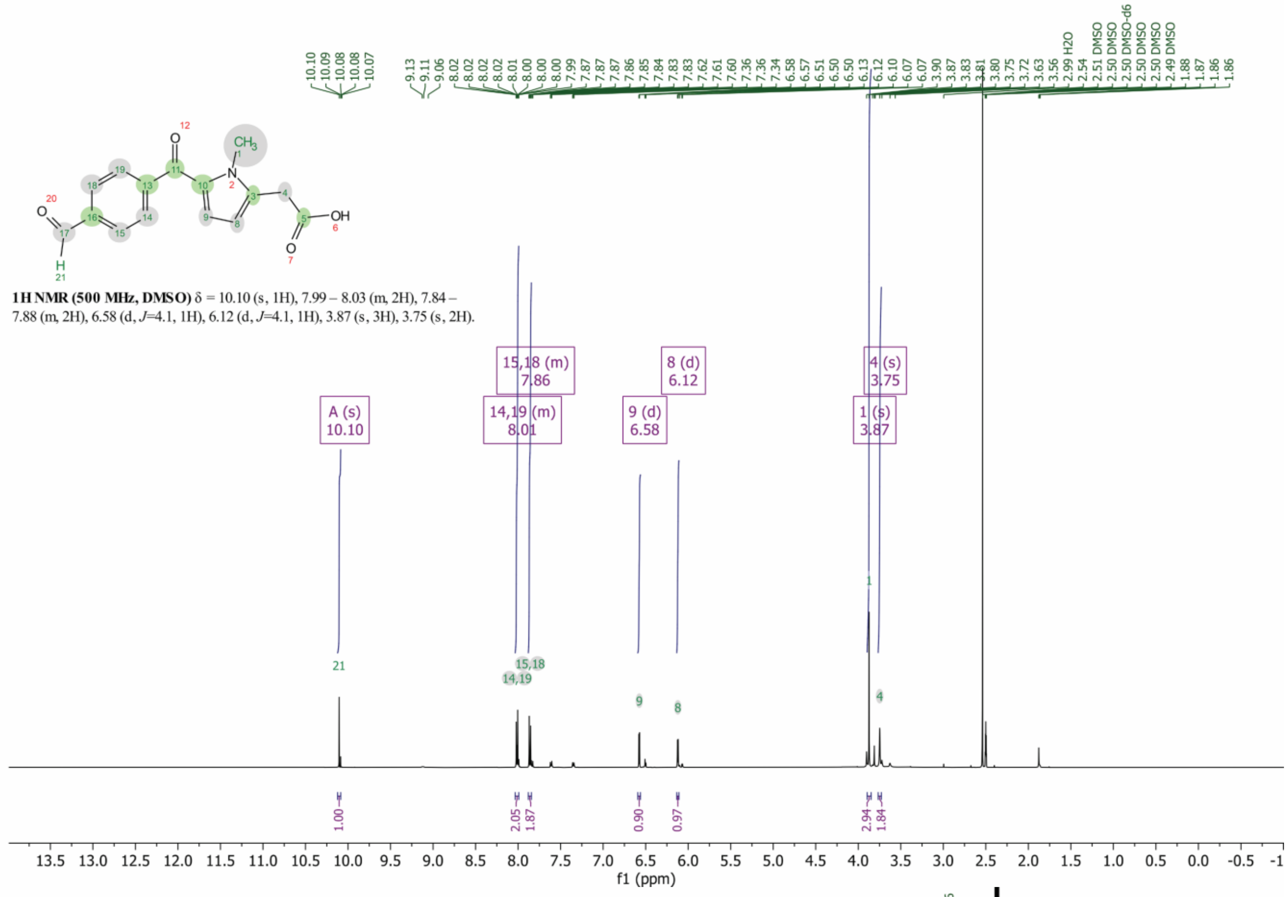

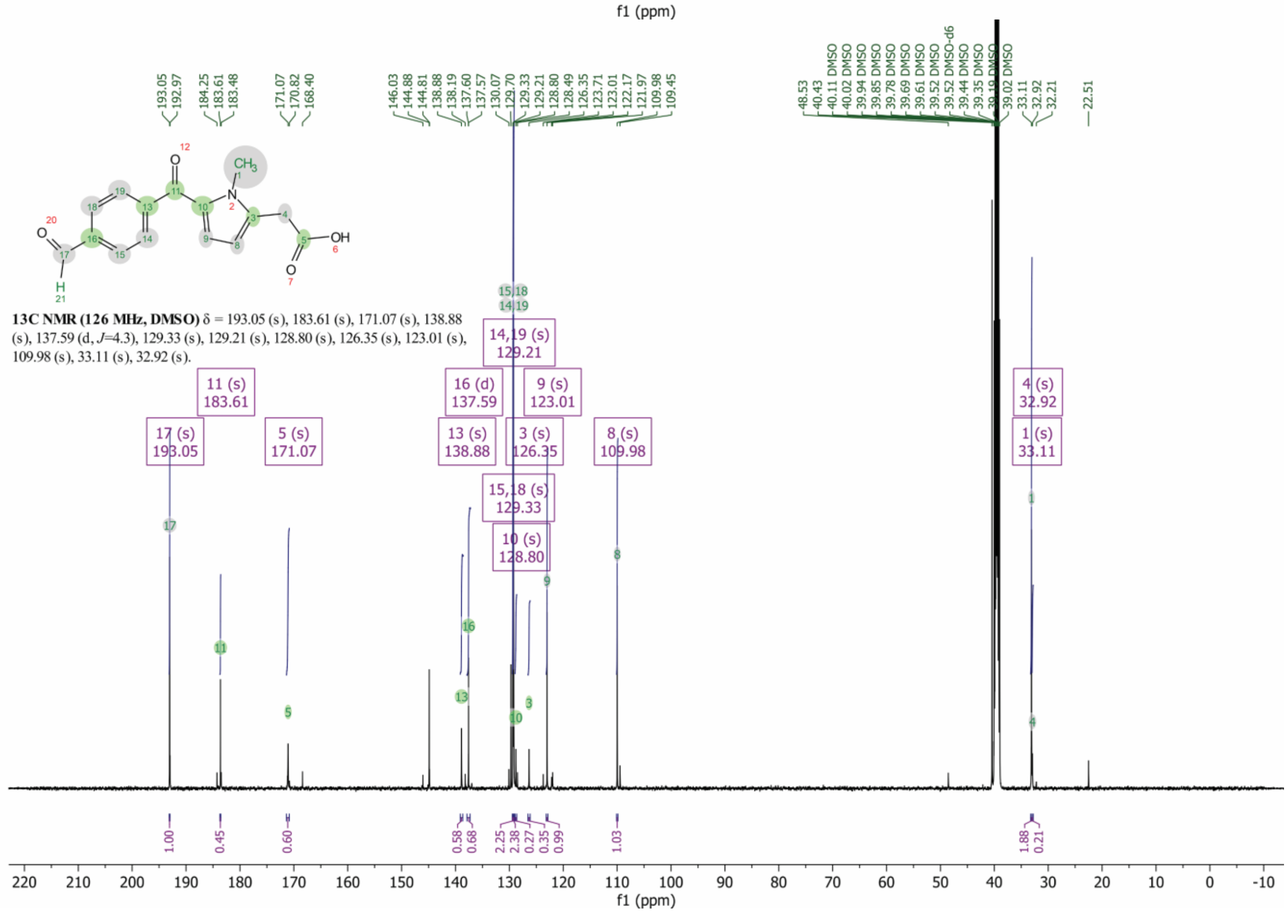

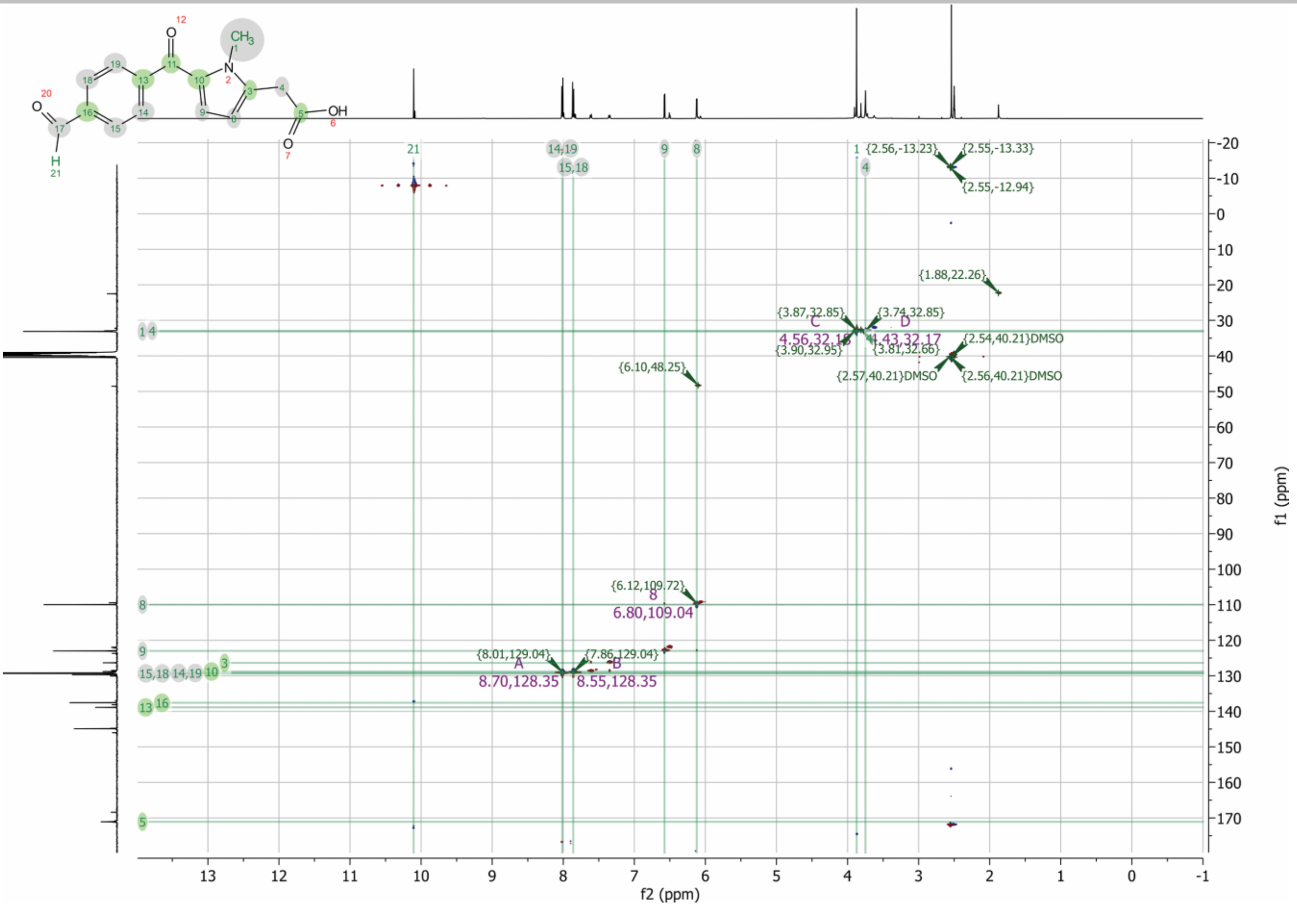
f2 (ppm)
f1 (ppm)
6.80,109.04
8.70,128.35
8.55,128.35
4.56,32.18
1.43,32.17

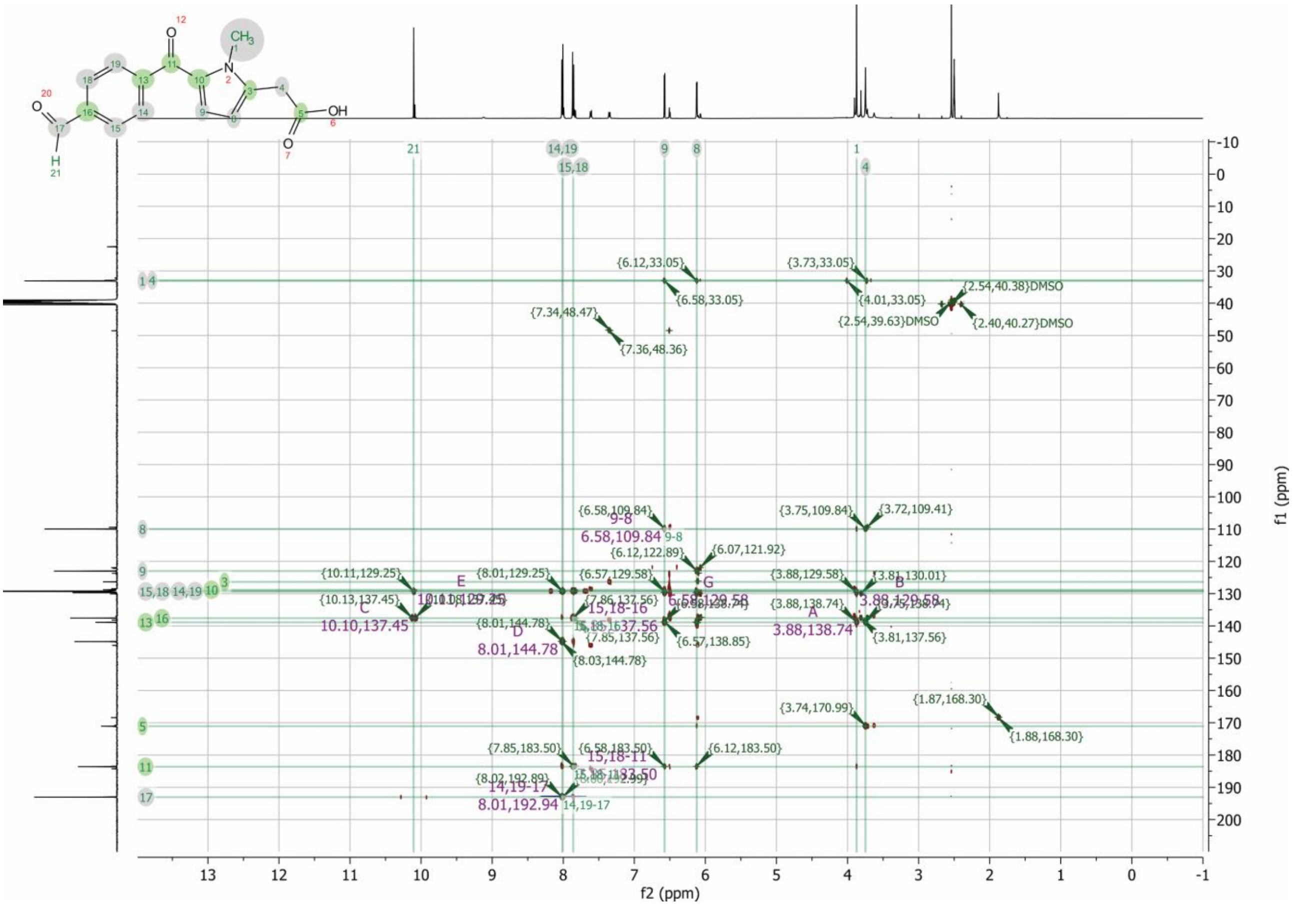
f2 (ppm)
f1 (ppm)
6.58,109.84
10.10,137.45
8.01,144.78
3.88,138.74
8.01,192.94

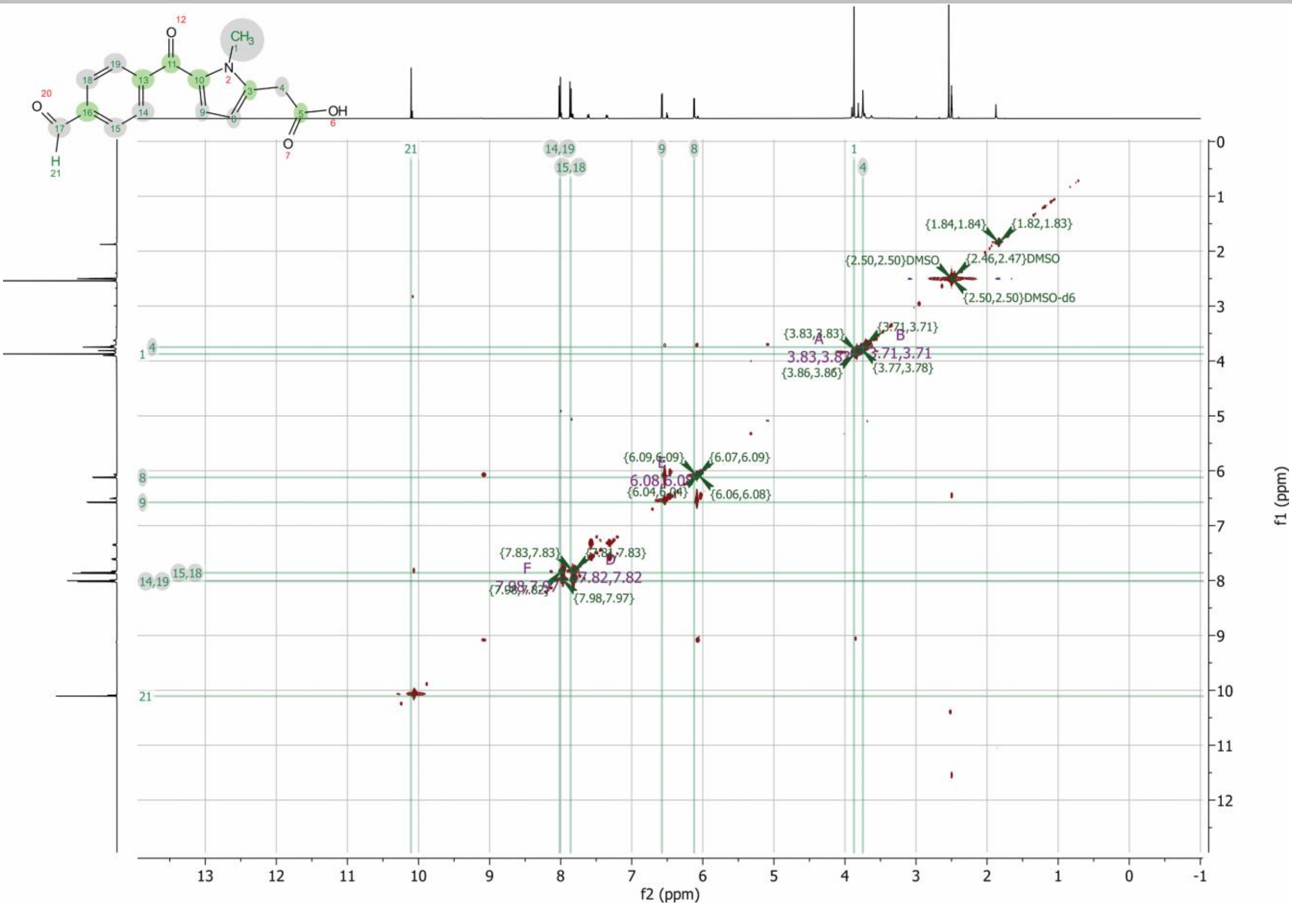

S2.2.4. 4-(5-(carboxymethyl)-1-methyl-1*H*-pyrrole-2-carbonyl)benzoic acid (**7**)

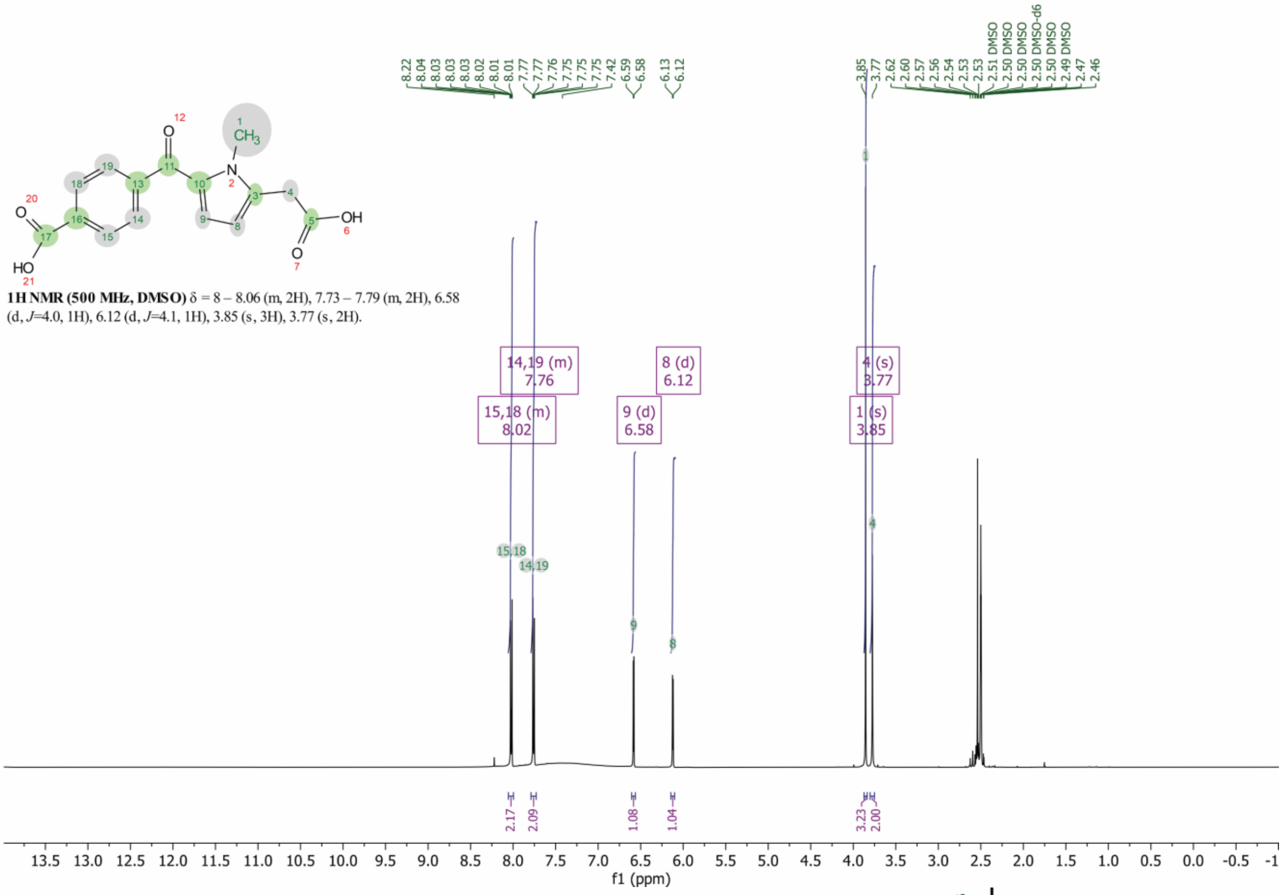

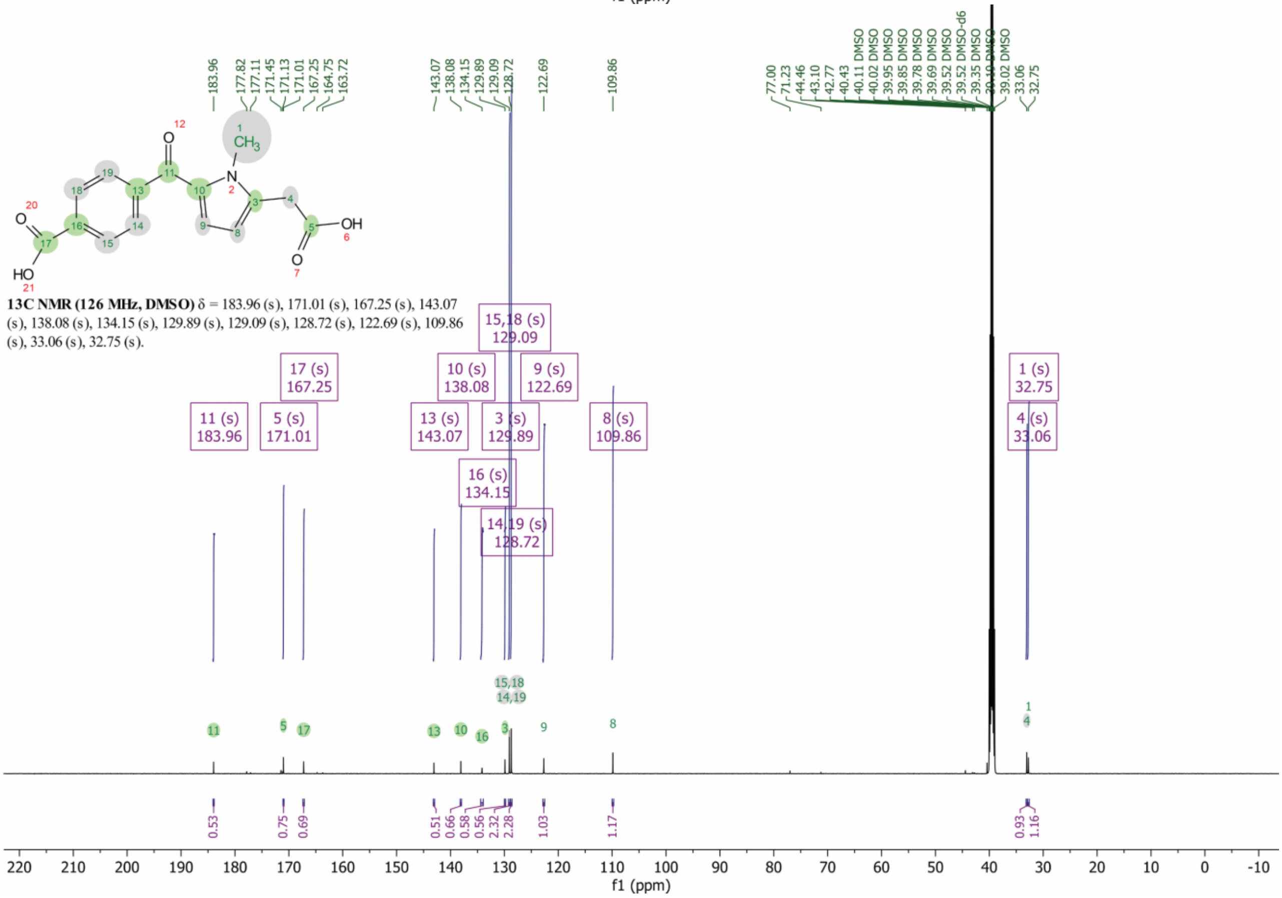

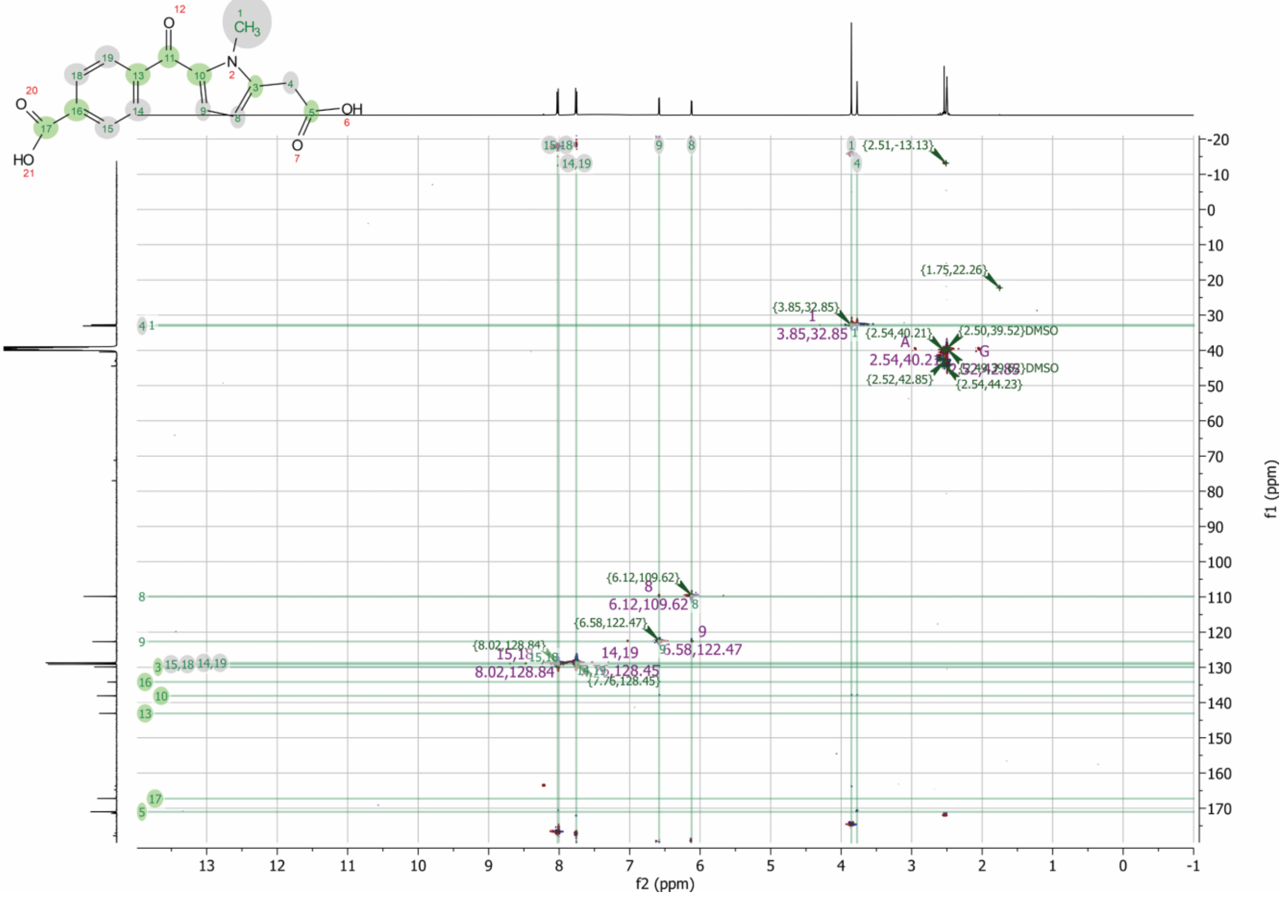
CH_3
N
O
OH
HO
f2 (ppm)
f1 (ppm)

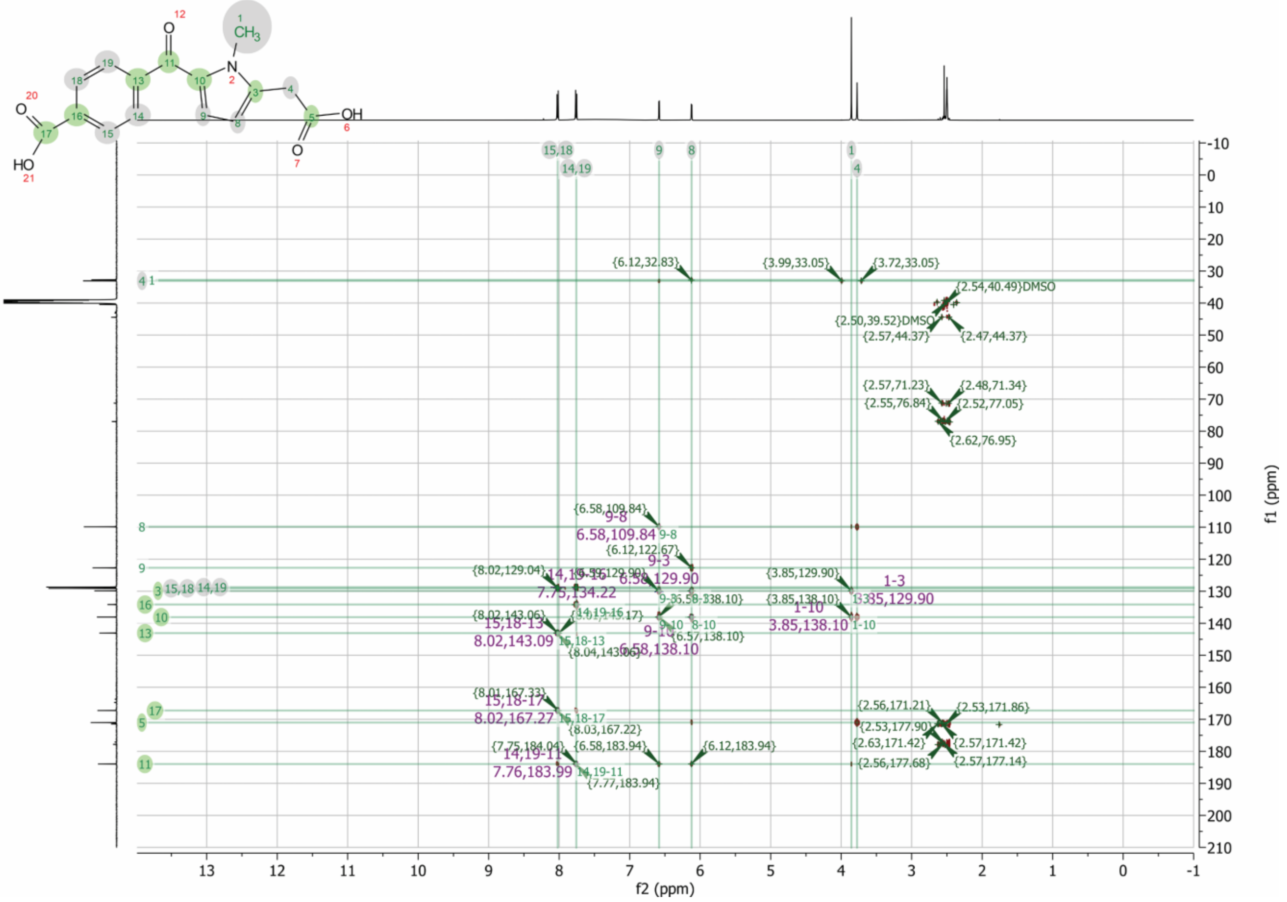
CH_3
N
O
OH
HO
f2 (ppm)
f1 (ppm)

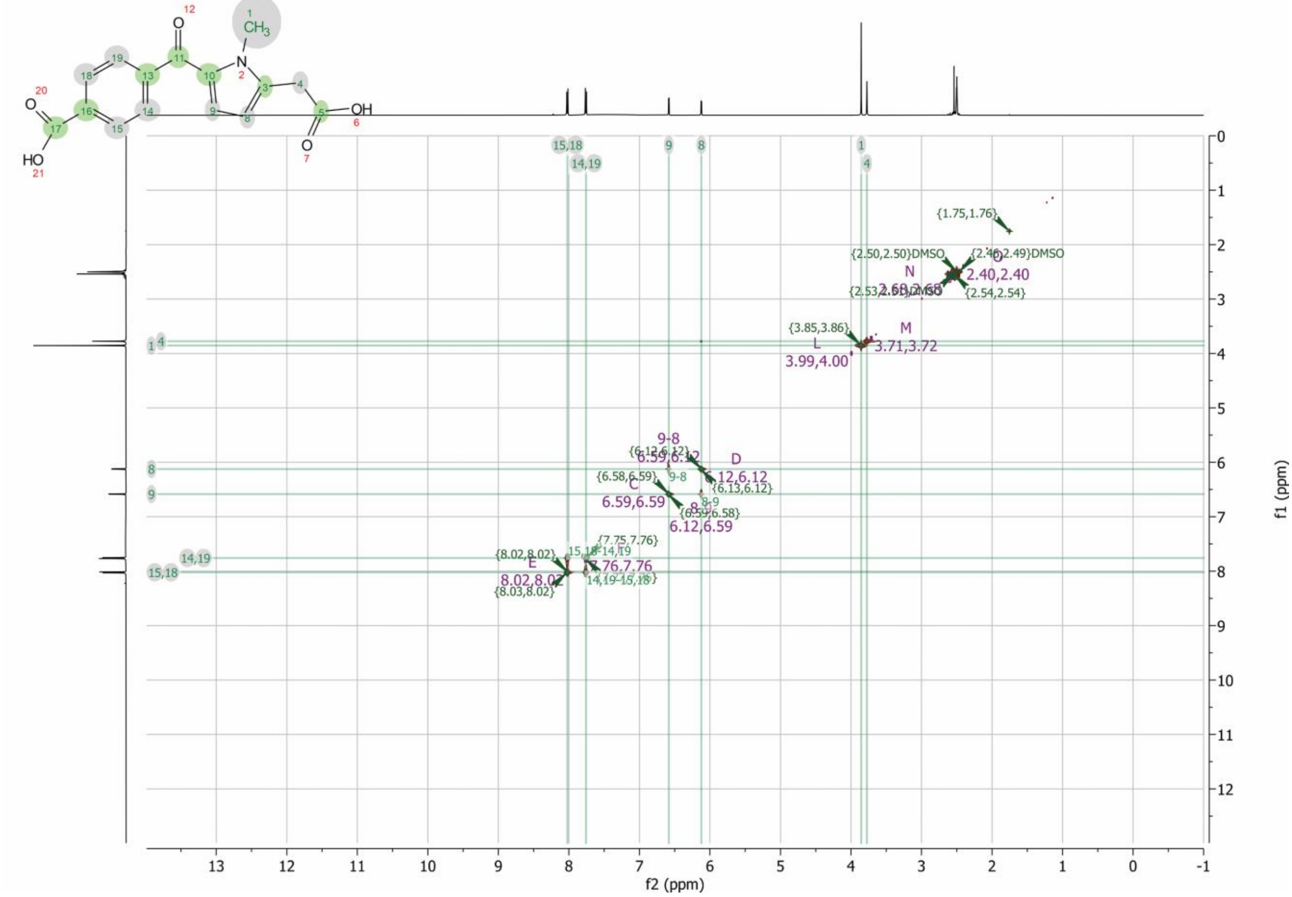
CH3
N
OH
O
HO
f2 (ppm)
f1 (ppm)
9-8
D
C
E
L
M
N
6.59,6.59
6.12,6.59
8.02,8.02
3.71,3.72
3.99,4.00
2.40,2.40
{1.75,1.76}
{2.50,2.50}DMSO
{3.85,3.86}
{7.75,7.76}
{8.02,8.02}
{8.03,8.02}
{6.58,6.59}
{6.13,6.12}
{2.54,2.54}

S2.3. UPLC-QTOF data obtained from the 64-panel drug high-throughput screening (Fig. S18)

All structures were tentatively assigned based on our UPLC-QTOF/MS^E data and available literature. They should be confirmed using standards or NMR analyses. Conversions (%) after 24 h are indicated.

S2.3.1. Empagliflozin (8).

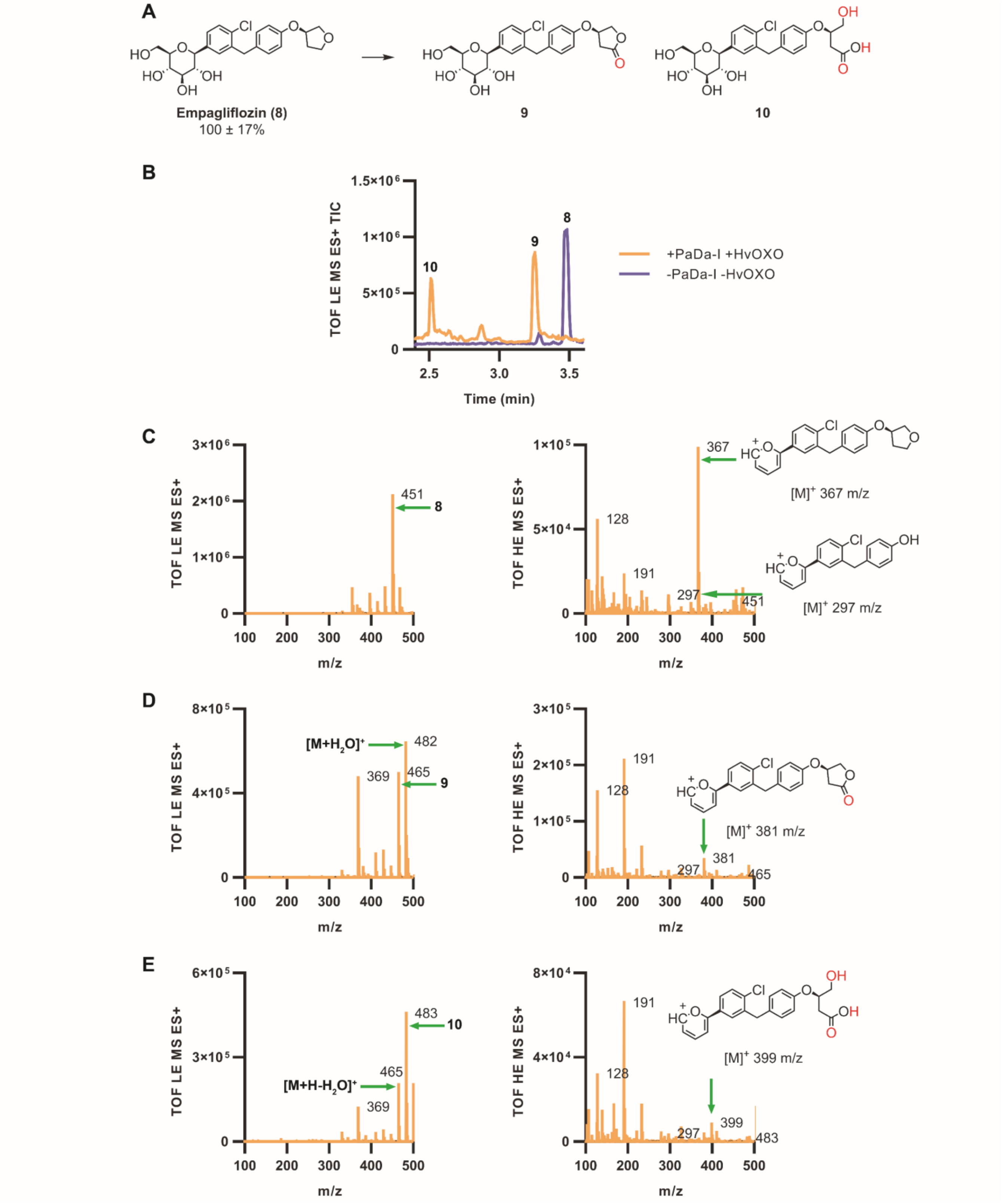

S2.3.2. Methotrimeprazine (11). The position of the hydroxyl group on the phenothiazine nucleus was not unequivocally determined.

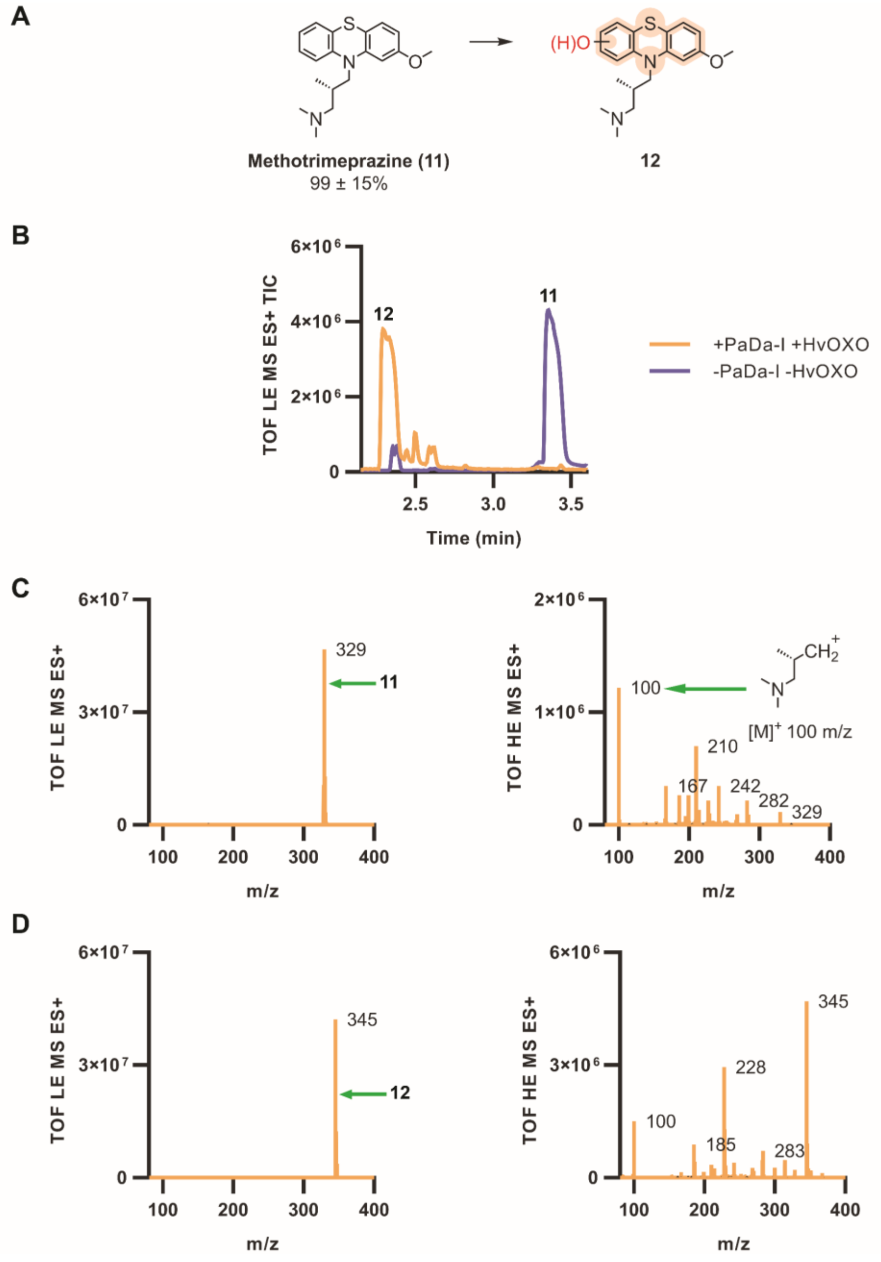

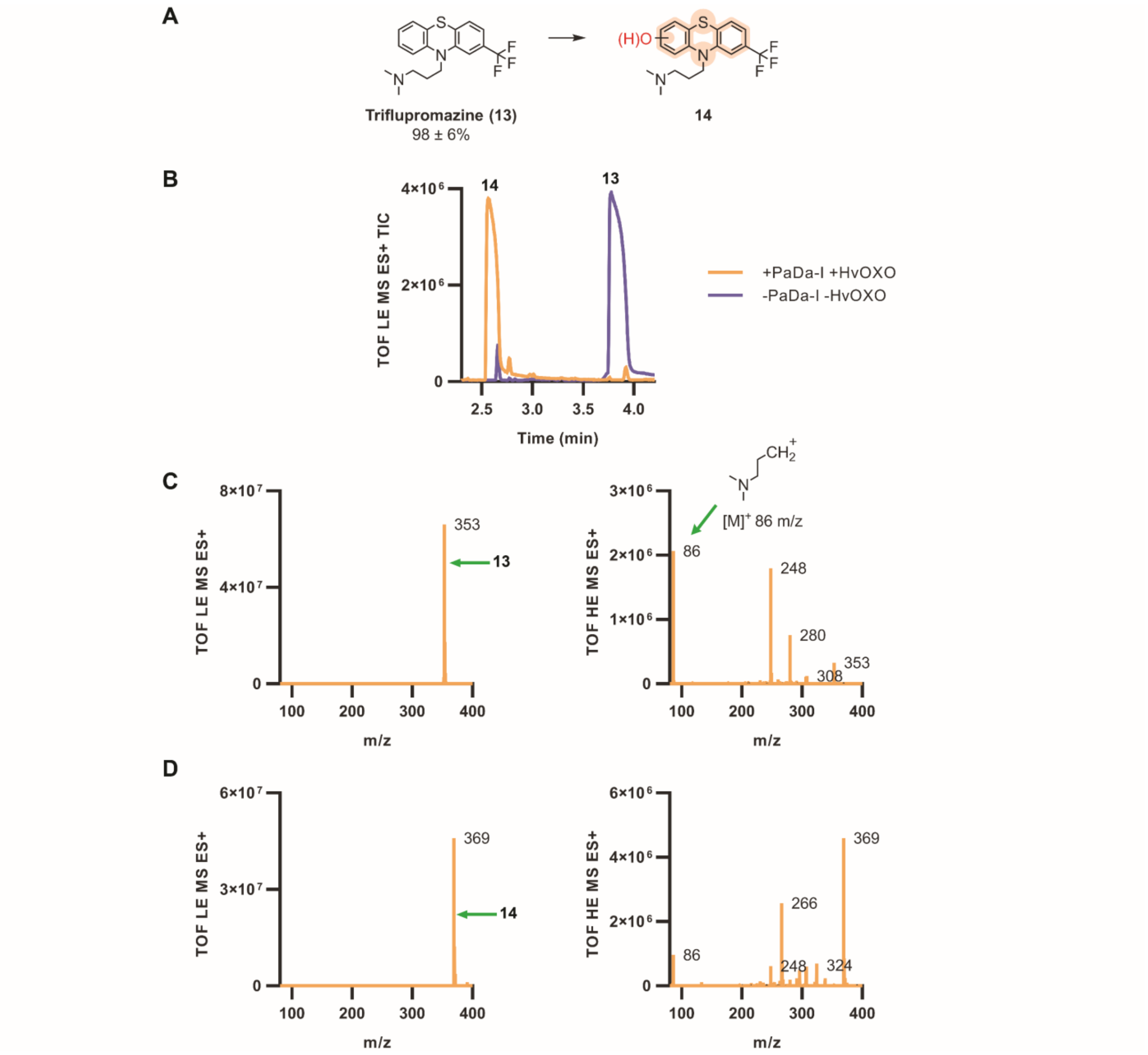

S2.3.3. Triflupromazine (13). The position of the hydroxyl group on the phenothiazine nucleus was not unequivocally determined.

S2.3.4. Fluvastatin (15). Functionalization sites on the indole ring of fluvastatin were not unequivocally identified. 5-Hydroxy- and 6-hydroxy-fluvastatin are main products in human liver microsomes.[9]

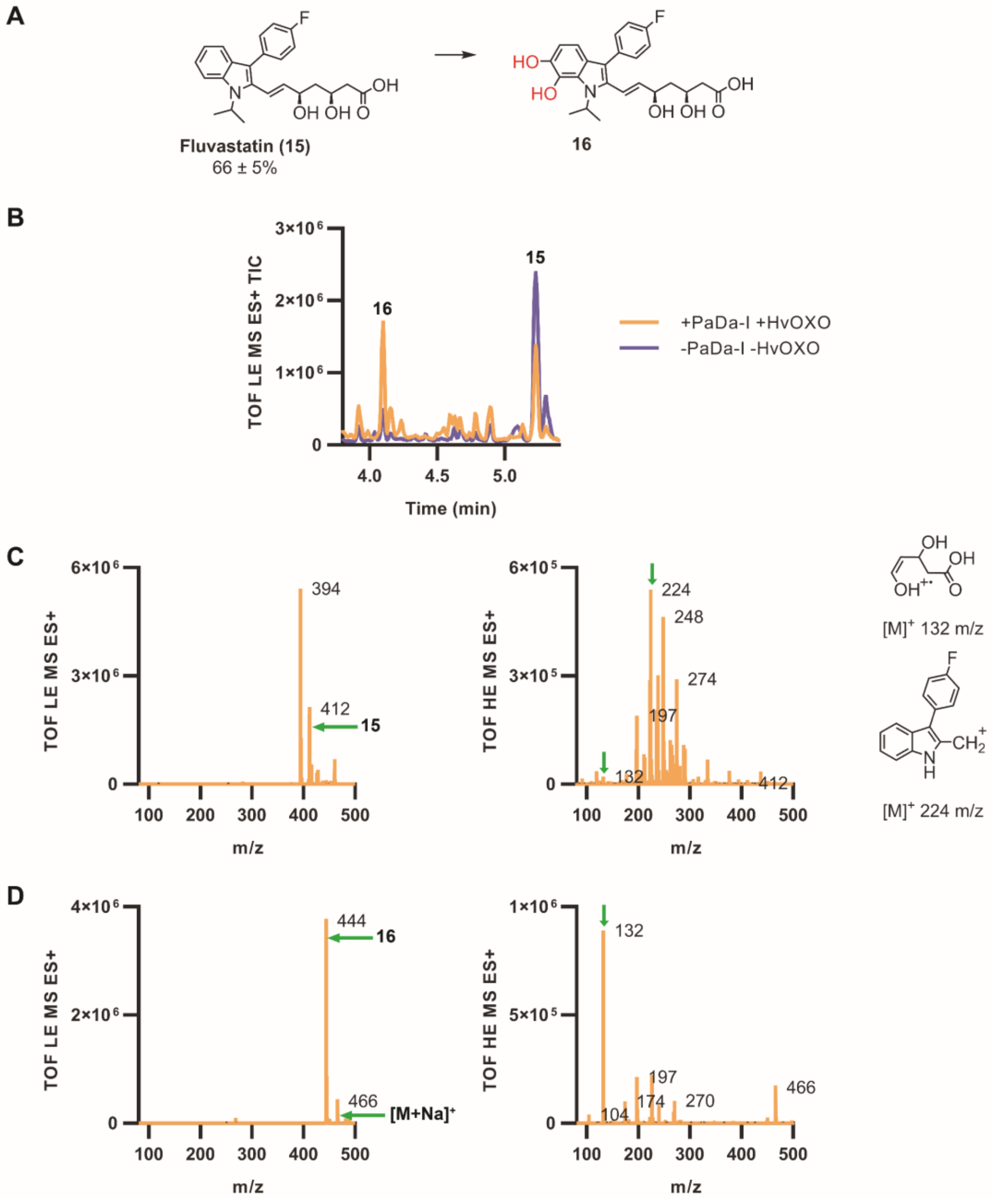

S2.3.5. Phenylbutazone (17). It was not unequivocally determined which carbon was hydroxylated by PaDa-I on the phenylbutazone butyl side chain. We are depicting below a major phenylbutazone derivative resulting from human metabolism. It exhibits the hydroxyl group on the *β*-position from the end of the aliphatic chain which was concluded based on a comparison with a synthesized standard.[10]

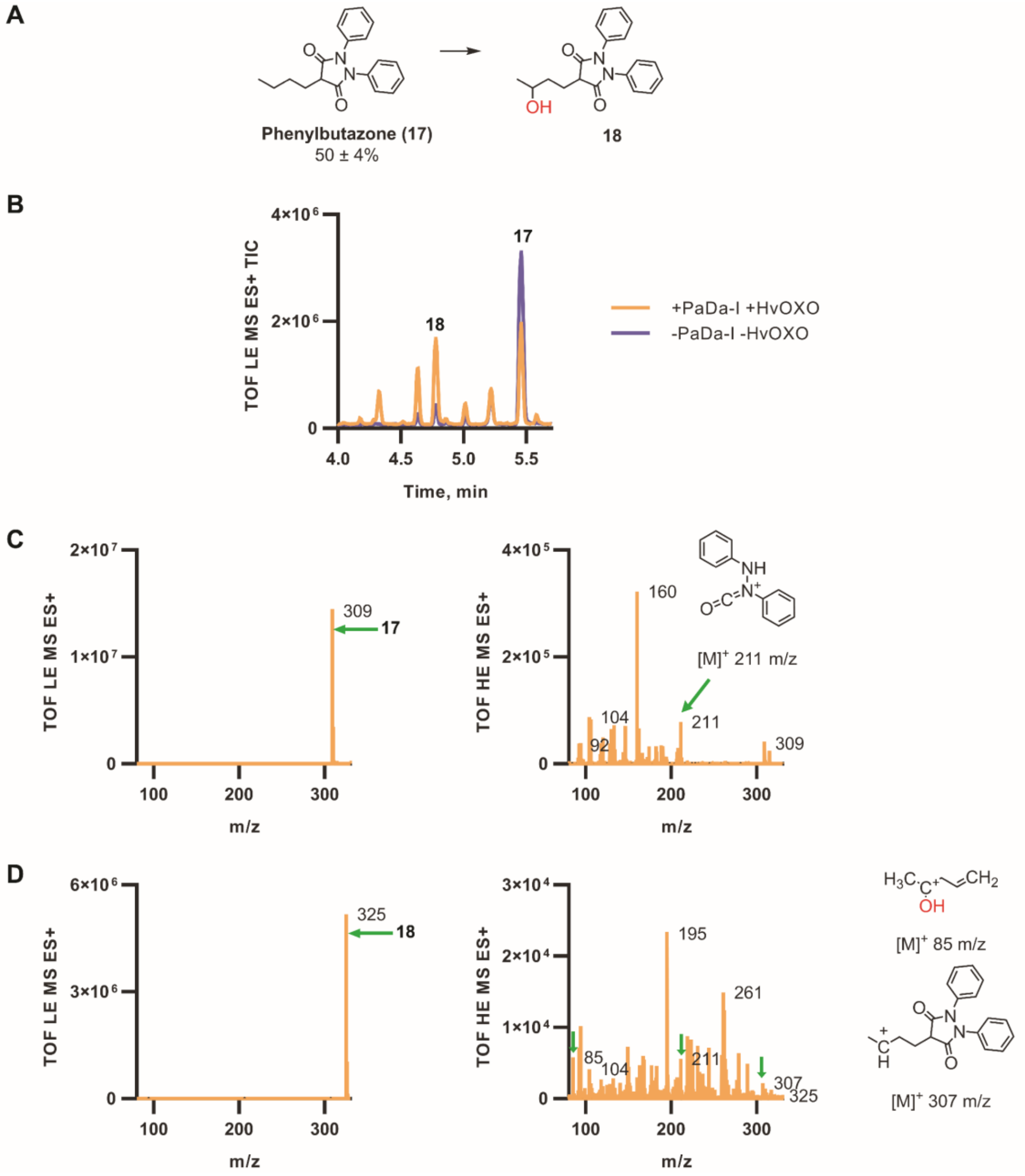

S2.3.6. Clozapine (19).

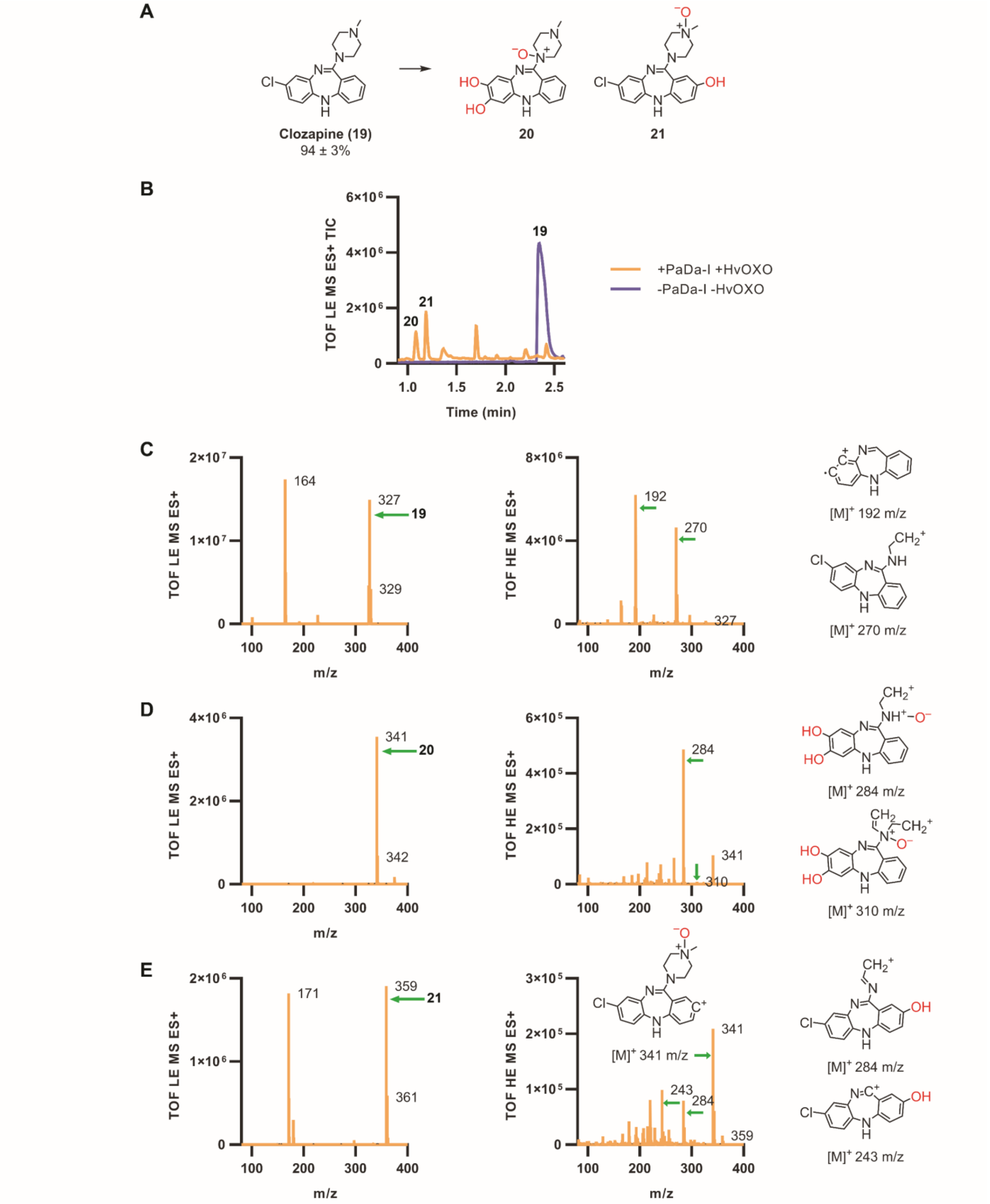

S2.3.7. 5-Benzyloxygramine (22).

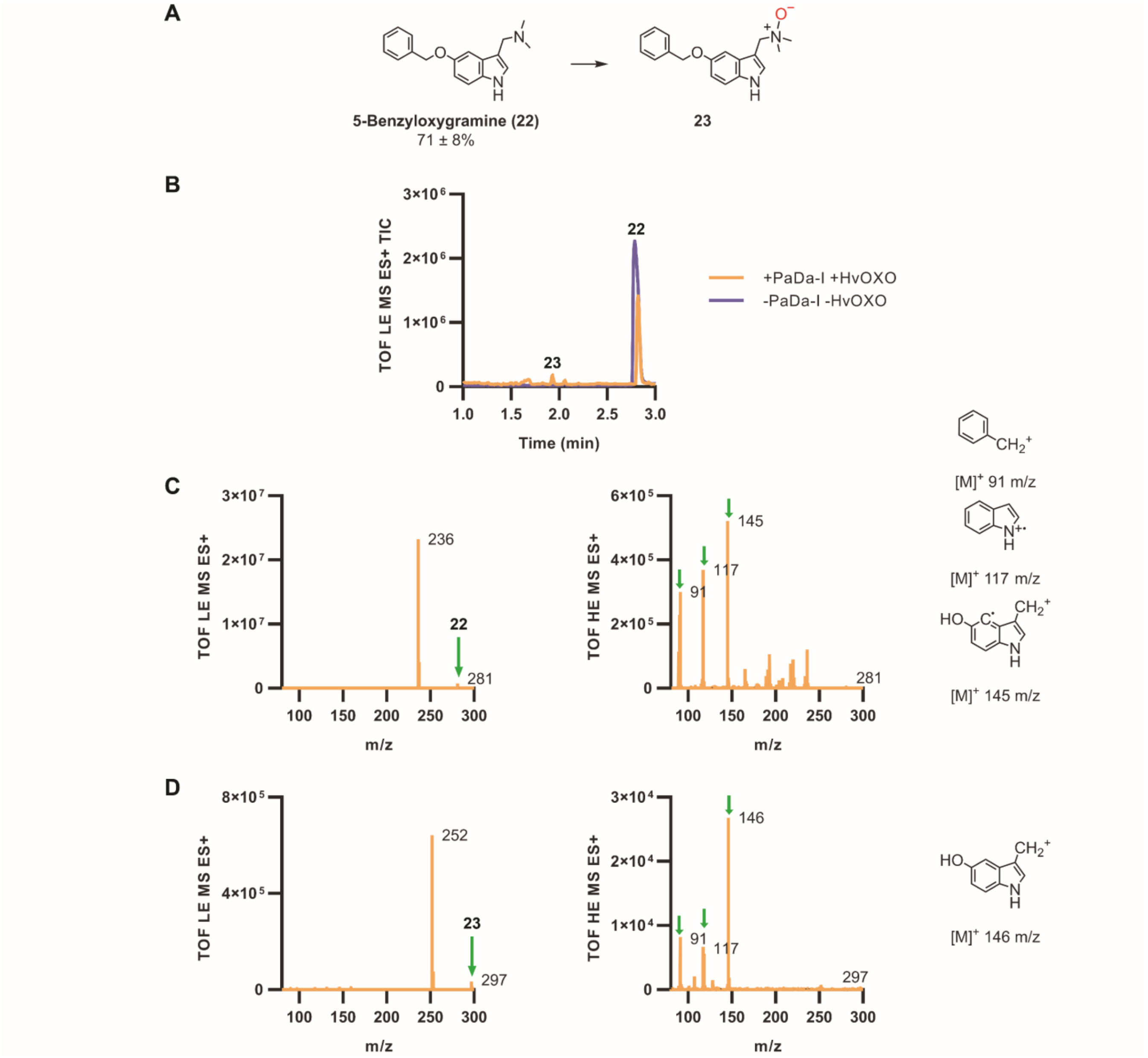

S2.3.8. Ketoconazole (24).

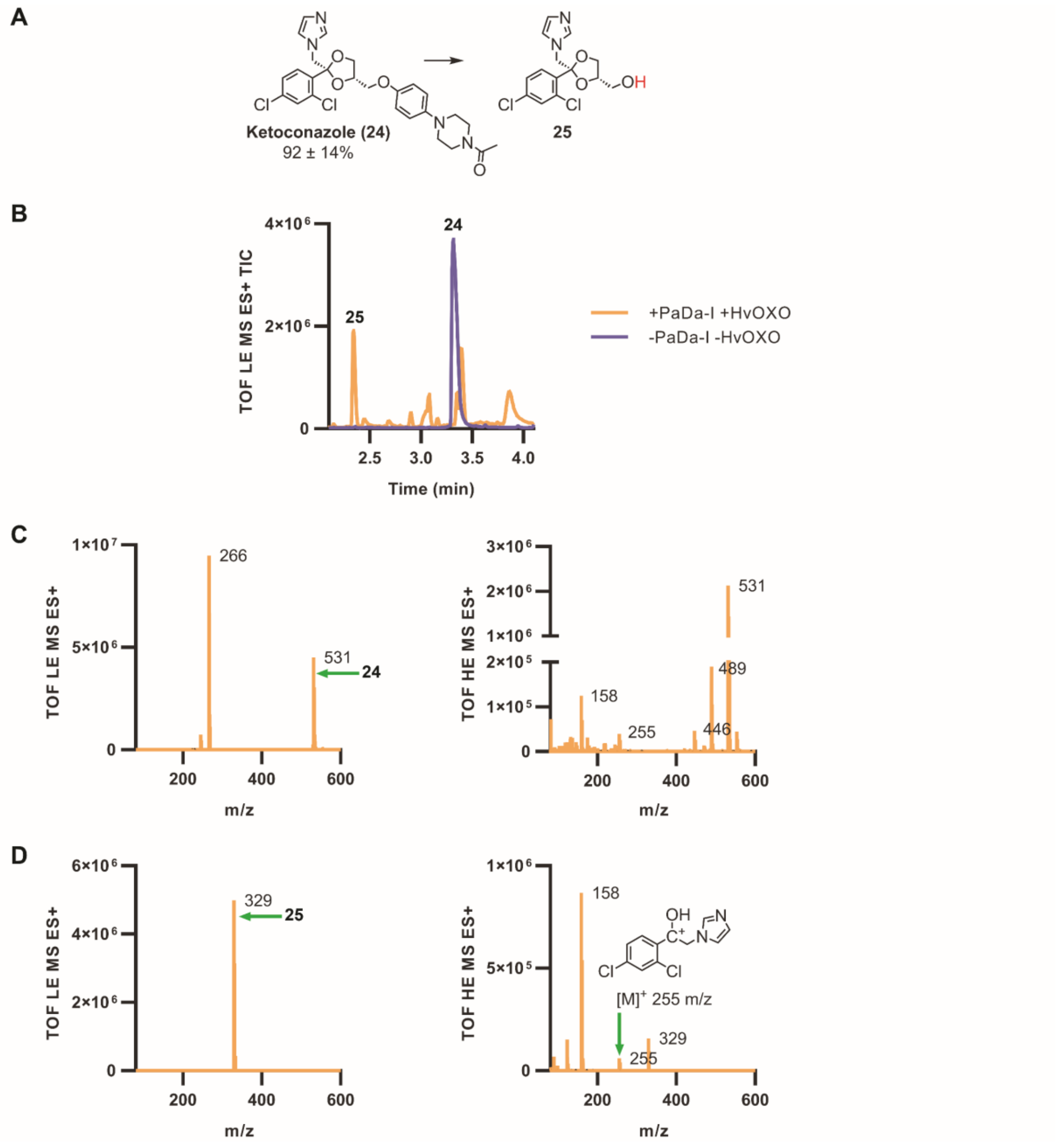

S2.3.9. Raloxifene (26). Dimerization site on raloxifene was not unequivocally identified. Herein, the major raloxifene dimer produced by CYP3A4, which was characterized by NMR, is shown as an example. In this case, a 1-electron oxidation of the raloxifene 4-hydroxyphenyl moiety took place to form an oxygen-centered radical. Another raloxifene molecule was oxidized on the benzo[*b*]thiophen-6-ol moiety to form an oxygen-centered radical which converted into the position 7 carbon-centered radical after delocalization. Non-enzymatic coupling of these radicals yielded the dimer.[11]

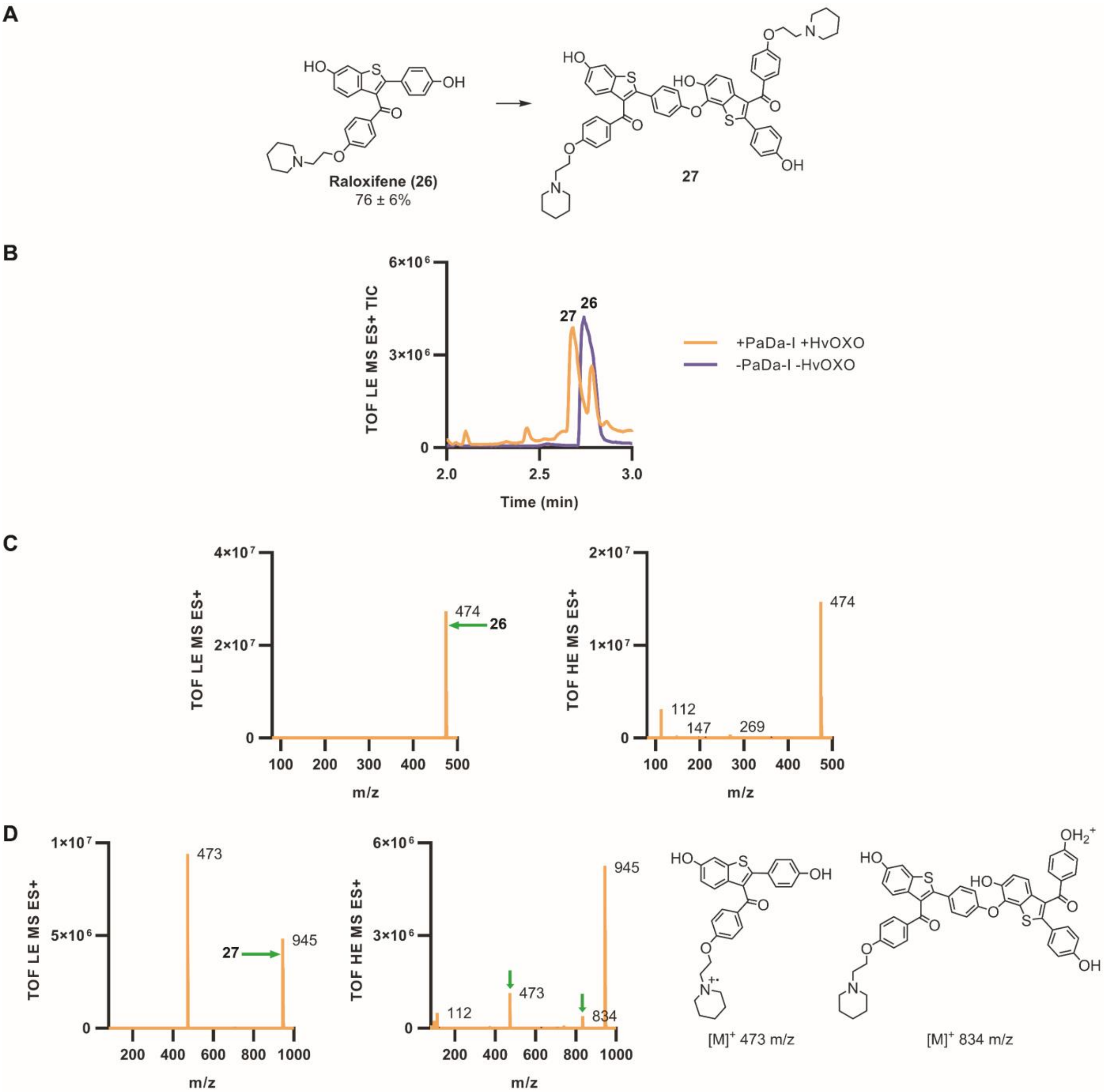

S2.3. Scheme

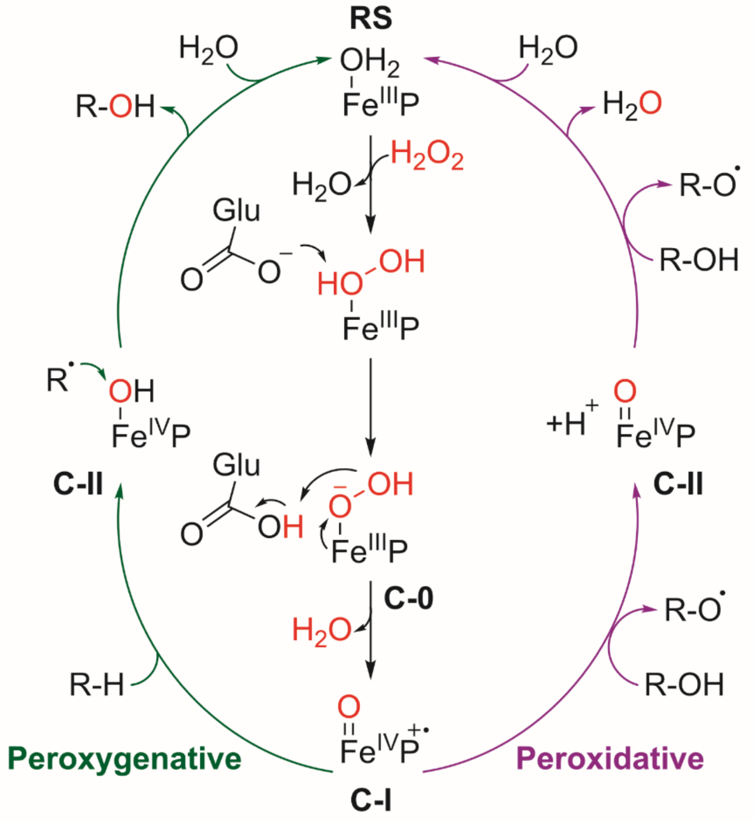

Scheme S1. Catalytic cycle of UPOs. UPOs contain iron protoporphyrin IX (**P**) in the active site. Ferric resting state (**RS**) of UPO reacts with H_2O_2 to form compound-0 (**C-0**). A glutamate residue (E196 in AaeUPO) facilitates both formation of C-0 and subsequent heterolytic peroxide cleavage to form oxoiron(IV) porphyrin radical cation known as compound-I (**C-I**). Depending on the UPO, substrate and reaction conditions, C-I participates in either peroxygenative (**left**) or peroxidative (**right**) reactions. In the peroxygenative pathway, C-I abstracts one electron and one proton from the substrate (**R-H**) to generate a substrate radical and the ferryl hydroxide form of UPO (**C-II**). Next, the substrate radical is hydroxylated by C-II (rebound mechanism). In the peroxidative route, both C-I and deprotonated C-II (in equilibrium with its protonated form) abstract one electron and one proton from the hydroxyl group of a substrate (**R-OH**). The resulting two substrate radicals are released from the enzyme active site. Modified based on Hofrichter *et al.*[12] Thus, UPOs combine reaction mechanisms similar to those observed for “classic” peroxidases[13] and P450 enzymes using the “peroxide shunt” or belonging to the peroxygenase family.[14]

S2.4. Tables

Table S1. Comparation of H_2O_2-generation systems for ethylbenzene hydroxylation catalysed by AaeUPO.

Type	Reactants/Devices	TON_{UPO}	By-product	Drawbacks	Concept	Ref.
**Chemical**	Au-Pd/TiO_2, H_2, O_2	51,400	H_2O, acetophenone	Requires precious metal catalyst	Au-Pd/TiO_2 converts H_2 and O_2 to H_2O_2	[15]
**Electrochemical**	Reactor with carbon-based gas diffusion electrode, O_2	400,000	Acetophenone	HO• formation, UPO inactivation	O_2 electrochemical reduction	[16]
	Dielectric barrier discharge plasma, O_2	13,787		UPO inactivation, low TON	Plasma induces H_2O_2 formation. Buffer treated with plasma is added to UPO sample	[17]
**Photochemical**	Rutile Au-TiO_2, H_2O, O_2, hv	21,000	Acetophenone	HO• formation, requires precious metal catalyst	Au-TiO_2 oxidizes H_2O to 1/2 O_2, $2H^+$ and 2 e^-, which form H_2O_2	[18]
	g-C_3N_4, formate, O_2, hv	60,000	Acetophenone	HO• formation	Formate is e^- donor	[19]
	FMN, EDTA, O_2, hv	11,470	Oxidized EDTA, acetophenone	Low TON	Photoexcited FMN oxidizes EDTA. Reduced FMN converts O_2 to H_2O_2	[20]
**Mechanical (piezobiocatalysis)**	BiOCl, H_2O, O_2, ultrasound	2,002	Acetophenone	HO• formation, rapid UPO inactivation, low TON	BiOCl and ultrasound produce e^- and holes, which react with O_2 and H_2O, respectively	[21]
**Photoelectrochemical**	Photovoltaic-photoelectrochemical tandem cell, H_2O, O_2, hv	43,300		HO• formation	Cell reduces O_2 using H_2O as an e^- donor and light	[22]
	Reactor with lumichrome tethered on single-walled carbon nanotubes as an electrode, O_2, hv	123,900	Acetophenone		Photoexcited lumichrome accepts e^- from cathode and use them to reduce O_2 to H_2O_2	[23]
	Mo-doped $BiVO_4$ photoanode and an inverse opal ITO cathode, H_2O, hv	10,000		HO• formation, low TON	$BiVO_4$ under illumination performs H_2O oxidation to generate H_2O_2	[24]
**Photoenzymatic**	Phenosafranine/methylene blue/FMN, formate dehydrogenase, formate, NAD^+, O_2, hv	25,000/ 39,920/ 6,490	CO_2	Rapid formate dehydrogenase inactivation, photocatalyst photobleaching	Formate dehydrogenase produces NADH, which is used by photocatalyst to reduce O_2 to H_2O_2	[25]
**Enzymatic**	Alcohol oxidase, formaldehyde dismutase, formate dehydrogenase, 3-hydroxybenzoate-6-hydroxylase, methanol, 2,5-dihydroxybenzoic acid, NAD^+, O_2	294,700	Acetophenone	Formate dehydrogenase has high $K_{m(formaldehyde)}$	Enzyme cascade converts methanol into CO_2 and H_2O_2	[26]
	Formate oxidase, formate, O_2	31,800	CO_2	Oxidase inactivation, high $K_{m(formate)}$	Oxidase converts formate into CO_2 and H_2O_2	[27]
	Formate oxidase, methanol, O_2	30,000	CO_2	Oxidase inactivation, high $K_{m(methanol)}$	Oxidase converts methanol into CO_2 and H_2O_2	[28]
	Sulfite oxidase, $CaSO_3$, O_2	30,800	Acetophenone, SO_4^{2-}	SO_3^{2-} reaction with H_2O_2 or O_2, oxidase inactivation	Oxidase converts SO_3^{2-} to SO_4^{2-} and H_2O_2	[29]

TON_{UPO} are µmoles of product divided by µmoles of AaeUPO. EDTA, ethylenediaminetetraacetic acid.

Table S2. Drug stocks prepared for 5 µL-scale reactions (**Fig. S18-19**).

[Stock] mM	Drug[a]	Solvent
10	Tolmetin (**4**), empagliflozin (**8**), phenacetin (**30**), methotrimeprazine (**11**), lenalidomide (**31**), ximelagatran (**32**), clozapine (**19**), ketoconazole (**24**), raloxifene (**26**), 5-benzyloxygramine (**22**), fluvastatin (**15**), phenylbutazone (**17**), capecitabine (**33**), labetalol (**34**), ramipril (**36**), indomethacin (**39**), zomepirac (**40**), chelerythrine (**41**), minaprine (**43**), sildenafil (**47**), imatinib (**49**), diclofenac (**50**), ampicillin (**51**), atenolol (**56**), brimonidine (**57**), haloperidol (**68**), idazoxan (**69**), niraparib (**70**), noradrenaline (**71**), papaverine (**72**), pravastatin (**74**), ranitidine (**75**), rimonabant (**76**), strychnine (**78**), terbutaline (**80**), tocainide (**82**)	50% acetonitrile buffer[b]
	Eprosartan (**55**)	50% tetrahydrofuran buffer[b]
20	Triflupromazine (**13**), noscapine (**35**), benzydamine (**38**), chlorprothixene (**42**), carbamazepine (**44**), niflumic acid (**48**), repaglinide (**52**), acetylsalicylic acid (**53**), apremilast (**54**), artemisinin (**55**), budenoside (**58**), celecoxib (**59**), cycloheximide (**60**), danazol (**61**), donepezil (**62**), doxepin (**63**), fluticasone propionate (**66**), furosemide (**67**), pheniramine (**73**), sitagliptin (**77**), tamibarotene (**79**), testosterone (**81**)	Acetonitrile
	Deferasirox (**37**), zaprinast (**45**) montelukast (**46**), estradiol (**65**), tranilast (**83**)	Tetrahydrofuran

[a] Drug structures are shown in **Fig. S18-19**.
[b] 100 mM citrate-phosphate pH 4.0.
First, acetonitrile was tested as a cosolvent for all drugs. Drugs which were not soluble in acetonitrile, were dissolved in tetrahydrofuran or 50% organic solvent. Final drug concentration in the PaDa-I reactions was 0.5 mM with 2.5% cosolvent in all cases. Thus, either 0.125 or 0.250 µL (from the 20 and 10 mM drug stock, respectively) were transferred to the 5 µL reaction (final volume).

S3. References

[1] P. Molina-Espeja, E. Garcia-Ruiz, D. Gonzalez-Perez, R. Ullrich, M. Hofrichter, M. Alcalde, *Appl. Environ. Microbiol.* **2014**, *80*, 3496-3507.
[2] M. D. Castillo, J. Stenstrom, P. Ander, *Anal. Biochem.* **1994**, *218*, 399-404.
[3] K. Huynh, C. L. Partch, *Curr. Protoc. Protein Sci.* **2015**, *79*, 28-29.
[4] T. C. McIlvaine, *J. Biol. Chem.* **1921**, *49*, 183-186.
[5] P. Molina-Espeja, S. Ma, D. M. Mate, R. Ludwig, M. Alcalde, *Enzyme Microb. Technol.* **2015**, *73*, 29-33.
[6] B. G. Lane, J. M. Dunwell, J. A. Ray, M. R. Schmitt, A. C. Cuming, *J. Biol. Chem.* **1993**, *268*, 12239-12242.
[7] O. Opaleye, R.-S. Rose, M. M. Whittaker, E.-J. Woo, J. W. Whittaker, R. W. Pickersgill, *J. Biol. Chem.* **2006**, *281*, 6428-6433.
[8] E. J. Woo, J. M. Dunwell, P. W. Goodenough, A. C. Marvier, R. W. Pickersgill, *Nat. Struct. Biol.* **2000**, *7*, 1036-1040.
[9] V. Fischer, L. Johanson, F. Heitz, R. Tullman, E. Graham, J.-P. Baldeck, W. T. Robinson, *Drug Metab. Dispos.* **1999**, *27*, 410-416.
[10] J. J. Burns, R. K. Rose, S. Goodwin, J. Reichenthal, E. C. Horning, B. B. Brodie, *J. Pharmacol. Exp. Ther.* **1955**, *113*, 481-489.
[11] H.-K. Lim, M. Yang, W. Lam, F. Xu, J. Chen, Y. Xu, H. U. Shetty, K. Yang, J. Silva, D. C. Evans, *Xenobiotica* **2012**, *42*, 737-747.
[12] M. Hofrichter, H. Kellner, R. Herzog, A. Karich, C. Liers, K. Scheibner, V. W. Kimani, R. Ullrich in *Grand challenges in fungal biotechnology* (Ed.: H. Nevalainen), Springer International Publishing, Cham, **2020**, pp. 369-403.
[13] N. C. Veitch, *Phytochemistry* **2004**, *65*, 249-259.
[14] a) O. Shoji, Y. Watanabe, *J. Biol. Inorg. Chem.* **2014**, *19*, 529-539; b) A. W. Munro, K. J. McLean, J. L. Grant, T. M. Makris, *Biochem. Soc. Trans.* **2018**, *46*, 183-196.
[15] S. J. Freakley, S. Kochius, J. Van Marwijk, C. Fenner, R. J. Lewis, K. Baldenius, S. S. Marais, D. J. Opperman, S. T. L. Harrison, M. Alcalde, M. S. S., G. J. H., *Nat. Commun.* **2019**, *10*, 4178.
[16] A. E. W. Horst, S. Bormann, J. Meyer, M. Steinhagen, R. Ludwig, A. Drews, M. Ansorge-Schumacher, D. Holtmann, *J. Mol. Catal. B Enzym.* **2016**, *133*, S137-S142.
[17] A. Yayci, Á. G. Baraibar, M. Krewing, E. F. Fueyo, F. Hollmann, M. Alcalde, R. Kourist, J. E. Bandow, *ChemSusChem* **2020**, *13* 2072-2079.
[18] W. Zhang, E. Fernández-Fueyo, Y. Ni, M. van Schie, J. Gacs, R. Renirie, R. Wever, F. G. Mutti, D. Rother, M. Alcalde, *Nat. Catal.* **2018**, *1*, 55.
[19] M. M. C. H. van Schie, W. Zhang, F. Tieves, D. S. Choi, C. B. Park, B. O. Burek, J. Z. Bloh, I. W. C. E. Arends, C. E. Paul, M. Alcalde, *ACS Catal.* **2019**, *9*, 7409-7417.
[20] E. Churakova, M. Kluge, R. Ullrich, I. Arends, M. Hofrichter, F. Hollmann, *Angew. Chem. Int. Ed.* **2011**, *50*, 10716-10719.
[21] J. Yoon, J. Kim, F. Tieves, W. Zhang, M. Alcalde, F. Hollmann, C. B. Park, *ACS Catal.* **2020**, *10*, 5236-5242.
[22] D. S. Choi, H. Lee, F. Tieves, Y. W. Lee, E. J. Son, W. Zhang, B. Shin, F. Hollmann, C. B. Park, *ACS Catal.* **2019**, *9*, 10562-10566.
[23] D. S. Choi, Y. Ni, E. Fernández-Fueyo, M. Lee, F. Hollmann, C. B. Park, *ACS Catal.* **2017**, *7*, 1563-1567.
[24] D. S. Choi, J. Kim, F. Hollmann, C. B. Park, **2020**, *132*, 16020-16024.
[25] S. J.-P. Willot, E. Fernandez-Fueyo, F. Tieves, M. Pesic, M. Alcalde, I. W. C. E. Arends, C. B. Park, F. Hollmann, *ACS Catal.* **2019**, *9*, 890-894.
[26] Y. Ni, E. Fernández‐Fueyo, A. G. Baraibar, R. Ullrich, M. Hofrichter, H. Yanase, M. Alcalde, W. J. H. van Berkel, F. Hollmann, *Angew. Chem. Int. Ed.* **2016**, *55*, 798-801.
[27] F. Tieves, S. J. P. Willot, M. M. C. H. van Schie, M. C. R. Rauch, S. H. H. Younes, W. Zhang, J. Dong, P. Gomez de Santos, J. M. Robbins, B. Bommarius, M. Alcalde, A. S. Bommarius, F. Hollmann, *Angew. Chem. Int. Ed.* **2019**, *58*, 7873-7877.
[28] S. J.-P. Willot, M. D. Hoang, C. E. Paul, M. Alcalde, I. W. C. E. Arends, A. S. Bommarius, B. Bommarius, F. Hollmann, *ChemCatChem* **2020**, *12*, 2713-2716.
[29] M. M. C. H. van Schie, A. T. Kaczmarek, F. Tieves, P. Gomez de Santos, C. E. Paul, I. W. C. E. Arends, M. Alcalde, G. Schwarz, F. Hollmann, *ChemCatChem* **2020**, *12*, 3186-3189.

S4. Author Contributions

E.R. performed the experimental work, which was supervised by the rest of the authors. All authors contributed to experiment design and manuscript preparation.
